# BAP1 deficiency-induced SKA3 promotes clear cell renal cell carcinoma malignant progression by regulating chromosomal instability

**DOI:** 10.1038/s41419-026-09076-3

**Published:** 2026-07-15

**Authors:** Yueyang Wang, Chong Lai, Lingling Wang, Jingwen Deng, Zhou Tian, Zhiyong Liang, Honghe Zhang

**Affiliations:** 1 Department of Pathology, Peking Union Medical College Hospital, Chinese Academy of Medical Science & Peking Union Medical College, Beijing, China; 2 Research Unit of Intelligence Classification of Tumor Pathology and Precision Therapy, Chinese Academy of Medical Sciences (2019RU042) and Zhejiang University School of Medicine, Hangzhou, China; 3 Department of Pathology, Zhejiang University School of Medicine, Hangzhou, China; 4 Department of Urology, the First Affiliated Hospital, Zhejiang University School of Medicine, Hangzhou, China; 5 Department of Medical Oncology, Sir Run Run Shaw Hospital, School of Medicine, Zhejiang University, Hangzhou, China; 6 Department of Surgery, The Fourth Affiliated Hospital, International Institutes of Medicine, Zhejiang University School of Medicine, Yiwu, China

**Keywords:** Oncogenes, Chromosome segregation, Renal cell carcinoma

## Abstract

Chromosomal instability (CIN), a hallmark of malignancy, remains poorly understood in clear cell renal cell carcinoma (ccRCC). Here, we identify spindle and kinetochore-associated protein 3 (SKA3) as a critical regulator of CIN in ccRCC. BAP1 loss-of-function mutations, prevalent in ccRCC, drive aberrant SKA3 overexpression. Mechanistically, BAP1 deubiquitinates nuclear receptor co-repressor 1 (NCOR1) to enhance its recruitment to the *SKA3* promoter, thereby repressing *SKA3* transcription. Additionally, BAP1-mediated histone H2AK119 deubiquitination at the *TRIM25* promoter activates the expression of *TRIM25*, facilitating ubiquitin-proteasome-mediated degradation of SKA3. BAP1 deficiency disrupts both regulatory pathways, leading to aberrant accumulation of SKA3, which fuels CIN and correlates with metastasis progression and poor prognosis. In conclusion, our findings establish dysfunction of the BAP1-SKA3 axis as a molecular driver of CIN in ccRCC and suggest SKA3 as a potential therapeutic target for BAP1-mutant ccRCC.

## Introduction

Chromosomal instability (CIN), a pivotal characteristic of malignancy, arises from the failure to faithfully segregate chromosomes during mitosis. CIN results in numerical and structural chromosomal aberrations, driving extensive genomic rearrangements and copy number variations that fuel tumor evolution [1, 2]. Beyond genomic diversity, CIN activates the cyclic GMP-AMP synthase (cGAS) – stimulator of interferon genes (STING) signaling cascade, which remodels both tumor cell–intrinsic properties and microenvironmental crosstalk to drive distant metastasis [3, 4]. Key indicators of CIN include lagging chromosomes, chromosomal bridges, micronuclei, and aneuploidy. CIN is also linked to therapeutic resistance [5] and immunosuppression across multiple cancer types [6]. Notably, over 60% of malignancies exhibit CIN, which has been implicated as a driver of tumor progression in cancers such as prostate cancer [7] and osteosarcoma [8] et al. Despite these insights, the role of CIN in clear cell renal cell carcinoma (ccRCC) remains poorly defined.

Current understanding attributes CIN primarily to dysregulated expression or functional defects in mitosis-associated genes [9]. Importantly, the genetic background of a tumor varies across tissue types, and tissue-specific drivers shape these processes. For example, human papillomavirus (HPV) promotes CIN by inducing degradation of Centromere-associated protein E (CENP-E) in cervical, anal, and head and neck cancers [10], whereas FAT1 loss functions as a primary CIN driver in non-small cell lung cancer (NSCLC) [11]. Nevertheless, the specific molecular mechanisms by which genetic abnormalities drive CIN in the context of ccRCC have yet to be elucidated.

Metastatic ccRCC (m-ccRCC) is characterized by significant CIN and frequent abnormalities in mitosis-related genes [12, 13]. Additionally, several recurrent somatic mutations in ccRCC have been reported to be associated with processes such as replicative stress and mitotic abnormality [14, 15]. Notably, mutations in SETD2 and BAP1 are associated with more aggressive tumor phenotypes [16, 17], although the specific mechanisms underlying these features remain unclear.

As a core component of the spindle and kinetochore-associated (SKA) complex, SKA3 is essential for ensuring accurate mitotic progression [18, 19]. Although aberrant upregulation of SKA family members has been reported in hepatocellular carcinoma [20], laryngeal cancer [21], and cholangiocarcinoma [22], their expression pattern and functional roles in ccRCC have yet to be investigated [23]. In this study, we demonstrated that SKA3 is significantly overexpressed in ccRCC compared to normal kidney tissue and is associated with poor prognosis. Specifically, aberrant SKA3 upregulation drives CIN in ccRCC, which is regulated by BAP1 deficiency caused by somatic mutations.

## Materials and methods

### Clinical materials

Human samples were obtained from patients who underwent radical or partial Nephrectomy at the First Affiliated Hospital, Zhejiang University School of Medicine. All specimens were collected after resection and fixed in 10% neutral buffered formalin within 30 min for 24–48 h at room temperature. Finally, tissues were embedded in paraffin blocks for subsequent histological sectioning and examination. This study was approved by the Ethics Committee of Zhejiang University, and all patients have signed the informed consent form. This study was approved by the Institutional Ethics Committee of the First Affiliated Hospital, Zhejiang University School of Medicine (2021IIT923).

### Mouse models

All animal experiments were conducted in accordance with protocols approved by the Institutional Animal Care and Use Committee (IACUC) of Zhejiang University (Ethics Approval No. ZJU20240940) and followed the operational standards of the Zhejiang University Experimental Animal Center. Mice were purchased from GemPharmatech Co., Ltd. (Jiangsu, China) and fed five per cage under 22 ± 2 °C, humidity-controlled specific pathogen-free (SPF) conditions under a 12-h light/dark cycle at the Zhejiang University Experimental Animal Center. Sample sizes were chosen based on previous experience with similar experimental models in our laboratory. Exclusion criteria were established prior to the study. Specifically, mice were euthanized and excluded from further analysis if they showed signs of severe distress or reached the humane endpoints before the completion of the treatment. A blinded study design was used in the mouse tumor experiment to prevent bias during data collection and assessment.

For subcutaneous tumor models, 5 × 10^6^ of each group of OS-RC-2 cells suspended in 100 µL of PBS were subcutaneously injected into the right flanks of 4-week-old male BALB/c nude mice (*n* = 3 or 8 per group). Tumor growth was monitored every 5 day or weekly using caliper measurements. Mice bearing established tumors (approximately 100–150 mm³) were randomly allocated into groups including a vehicle control and two treatment groups, using a computer-generated random number sequence. Treatment is administered via oral gavage at a predetermined dose and schedule.

For orthotopic kidney implantation, 5 × 10^5^ EV or SKA3-OE group (*n* = 9) of cells in 40 µL PBS were injected beneath the left renal capsule of anesthetized BALB/c nude mice. Following inoculation, the mouse body weight was measured every other day, and the clinical conditions were monitored regularly. Tumor volume was calculated using the following formula: V = length × width × height × 0.523. On the 60th day after model establishment, the mice were humanely euthanized in accordance with the IACUC protocols.

### Cell proliferation, migration, and invasion assays

For cell proliferation assays, the same number of cells (approximately 1000–3000) were incubated in each well of a 96-well plate and cultured for different time periods (0, 24, 48, and 72 h). 10 μL CCK-8 reagent (Boster, AR1199, China) was added to each well, and the absorbance value was measured at 450 nm (OD450) by a microplate reader (Thermo Scientific 5250040, USA) after incubation in a constant temperature incubator at 37 °C for 2 h. For invasion and migration assays, 2 × 10^4^ cells were seeded onto an 8 μm Transwell insert and cultured in medium containing 1% FBS, which was then placed into a 24-well plate. The lower chamber of the plate was filled with medium containing 10% FBS. For the invasion assay, Matrigel (Corning, 354248, USA) was added to the upper chamber before seeding the cells. After 24–48 h of incubation, the upper chamber was removed, rinsed with PBS, and then fixed with 4% paraformaldehyde. Following crystal violet staining, cells inside the chamber were wiped off. Cells attached to the bottom of the chamber were photographed under a microscope. The number of cells in three 10× fields of view was counted using ImageJ software.

### Chromatin immunoprecipitation (ChIP) assay

ChIP assays were performed using the SimpleChIP^®^ Plus Sonication Chromatin IP Kit (56383, Cell Signaling Technology) according to the manufacturer’s instructions. Briefly, cells were cross-linked with 1% formaldehyde and quenched with glycine. The nuclear lysates were subjected to sonication to shear chromatin into fragments ranging from 200 to 1000 bp. The sheared chromatin was then incubated with NCOR1 Rabbit mAb (Cell Signaling Technology, #34271), Histone H3 Rabbit mAb (Cell Signaling Technology, #4620), H2AK119Ub antibody (Cell Signaling Technology, #8240), or normal Rabbit IgG (CST, #2729) at 4 °C for at least 4 h with rotation, and incubated with Protein G magnetic beads. After washing and elution, the chromatin DNA was purified and detected by specific SKA3 or TRIM25 promoter region primers by qPCR. The results were calculated as the percentage of input DNA.

### Luciferase activity assay

To evaluate the effect of BAP1 and NCOR1 on SKA3 transcriptional activity, we constructed a 2.5-kb fragment of the human *SKA3* promoter region into a pGL3-basic vector plasmid (Promega). HEK293T or ccRCC cells were seeded in 24-well plates and co-transfected with pGL3-basic or pGL3-promoter and pRL-TK vectors (as an internal control) using Lipofectamine LTX (Invitrogen, USA). After 48 h of transfection, Firefly and Renilla luciferase activities were detected using Dual-Luciferase Assay Reagent (Vazyme) according to the manufacturer’s instructions. The Firefly luciferase activity was normalized to the Renilla luciferase activity for each sample.

### Fluorescence lifetime imaging-microscopy-based fluorescence resonance energy transfer (FLIM-FRET)

FLIM experiments were performed using a Leica Stellaris 8 confocal microscope (×64 Oil, NA = 1.4, Leica Microsystems) with a HyD X detector and a pulsed white light laser. FLIM was performed on EGFP (donor) and mCherry (acceptor) co-transfected HEK293T cells with comparable intensity. For lifetime acquisition, EGFP was excited at 473 nm with a frame size of 512 × 512 pixels. Acquisition continued until a threshold of at least 500 photons per pixel or 5 × 10^6^ photons per field of view was reached. Data analysis was conducted using the Leica LAS X FLIM/FCS software. The fluorescence decay curves were fitted using a mono-exponential model (or multi-exponential when justified), and the goodness of fit was evaluated by the reduced chi-squared (*χ* ^2^) values (typically 0.8 < *χ* ^2^ < 1.3) and the residuals distribution. FRET efficiency (E) was calculated using the following formula:

$$E=(1-{\tau }_{{DA}}/{\tau }_{D})$$

where *τ*_D_ represented the unquenched donor lifetime of the donor in the donor-only group, and *τ*_DA_ represented the quenched donor lifetime of the donor in the presence of the FRET acceptor, and was calculated by fixing *τ*_D_.

### Living cell imaging

For live-cell imaging of mitosis, the SKA3-(GGGGS)3-GFP construct was cloned into the PCDH-CMV-EF1a-PURO vector. H2B-DPPVAT-mCherry was cloned into the pLVX vector. After packaging the lentiviruses with the two vectors and infecting 769-P and 786-O cells, stable ccRCC cells expressing SKA3-GFP and H2B-mCherry were obtained through drug selection. Cells were seeded in 35-mm glass-bottom dishes (Thermo), and imaging was performed using a Lattice LightSheet7 (Zeiss, Germany) equipped with a 40× water-immersion objective. The image size was set to 2048 × 2048 pixels. For mitosis, images were captured every 5 min for a total duration of 12 h. Laser lines at 488 nm and 561 nm were used to excite GFP and mCherry, respectively. During imaging, cells were maintained at 37 °C in a humidified atmosphere with 5% CO2.

### Quantification and statistical analysis

Unless otherwise specified, all quantitative data are expressed as the mean ± standard deviation (SD). Differences between groups were analyzed as follows: for continuous variables, paired samples were analyzed using Student’s paired *t*-test (normally distributed data) or Wilcoxon’s signed-rank test (non-normal data), while unpaired samples were assessed using independent Student’s *t*-test (parametric) or Mann–Whitney *U*-test (non-parametric). For experiments involving two independent factors (e.g., genotype and CIN phenotypes), two-way analysis of variance (ANOVA) was performed, followed by Sidak’s multiple comparisons test. In cases where multiple independent phenotypes were compared between two groups, multiple unpaired *t*-tests were used, with *p*-values adjusted for multiple comparisons using the Holm–Sidak method. For comparisons involving three or more groups with a single factor, one-way analysis of variance (ANOVA) was applied to normally distributed data with equal variances, and Dunnett’s test was used for pairwise comparisons. If assumptions were violated, the Kruskal–Wallis *H*-test was used, followed by post hoc analysis (Dunn’s test). All analyses were performed using the R software (version 4.3.2; R Foundation for Statistical Computing).

## Results

### SKA3 upregulation correlates with CIN in ccRCC

**Fig. 1:**
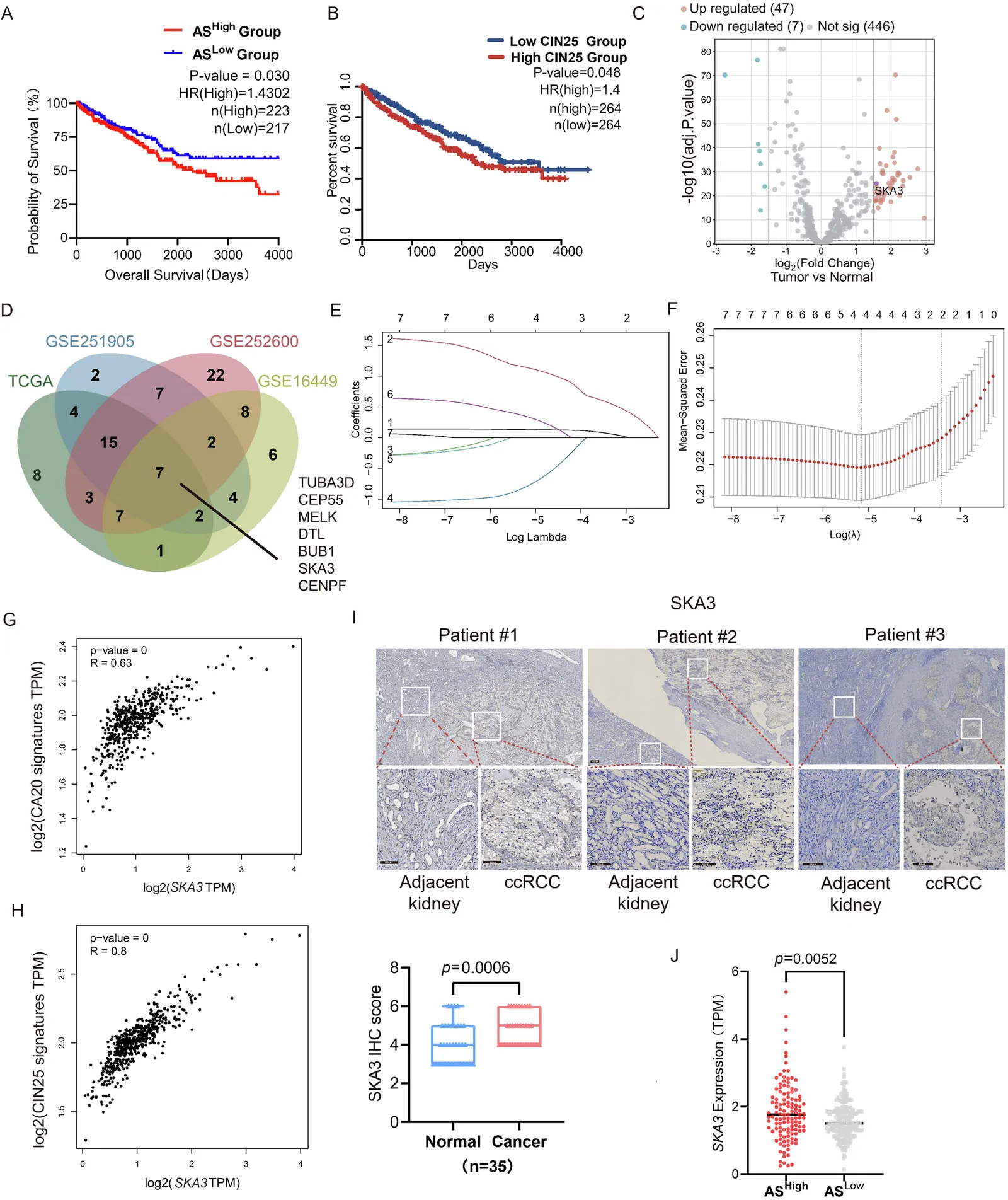
SKA3 upregulation correlates with CIN in ccRCC. **A** Kaplan–Meier (K–M) analysis of overall survival (OS) in ccRCC patients stratified by AS levels in the TCGA-KIRC cohort (*n* = 223 for the AS^High^ group, AS > 4, *n* = 217 for the AS^Low^ group, AS ≤ 3, log-rank test). **B** K–M analysis of OS in ccRCC patients stratified by CIN25 signature expression levels (*n* = 264 for CIN25^High^ group, *n* = 264 for CIN25^Low^ group, log-rank test). **C** Volcano plot showing differentially expressed mitosis-related genes between ccRCC tissues and normal kidney tissues in the TCGA-KIRC cohort (adjusted *p* < 0.05, |log_2_(FC)| > 1.5). **D** Venn diagram illustrating overlapping differentially expressed genes across four independent ccRCC datasets (TCGA-KIRC and 3 GEO datasets). **E** LASSO coefficient profiles of the seven mitosis-related genes. The horizontal axis represents the log of the penalty parameter (logλ), and the vertical axis represents the feature coefficients. Each colored line tracks the trajectory of an individual gene’s coefficient as the penalty varies; variables with weaker predictive power for metastasis are shrunk to zero; **F** Tuning parameter (λ) selection in the LASSO model via 10-fold cross-validation. The horizontal axis tracks logλ, and the vertical axis tracks the mean-squared error (MSE). Red dots indicate the mean cross-validation error, with error bars representing the standard errors. The two vertical dashed lines indicate the minimum criteria (λ_min_, left) and the one standard error criteria (λ_1se_, right); **G** Correlation analysis between *SKA3* mRNA expression and the CA20 gene signature. **H** Correlation analysis between *SKA3* mRNA expression and the CIN25 gene signature. **I** Representative immunohistochemical (IHC) staining images (up) and quantification (down) of SKA3 expression in 35 paired ccRCC and adjacent normal kidney tissues (*n* = 35). Scale bar = 100 μm. **J** Comparison of *SKA3* mRNA levels in AS^High^ and AS^Low^ samples of the TCGA-KIRC cohort. Data are presented as mean ± standard deviation (SD) or min to max whiskers. Statistical analysis: Log-rank test (**A**, **B**), Pearson’s correlation (**G**, **H**), Wilcoxon signed-rank test (**I**), Mann–Whitney *U*-test (**J**).

CIN is a hallmark of malignancy and a primary determinant of aggressive disease progression. To evaluate the clinical significance of CIN in ccRCC, we analyzed the aneuploidy score (AS) [24] using the TCGA-KIRC cohort, which showed that high AS was associated with poor overall survival (OS) (Fig. 1A). In addition, the elevated expression of a CIN-related gene signature (CIN25) [25] was associated with poor prognosis (Fig. 1B). These results reinforce the pivotal role of CIN in driving ccRCC malignancy.

Given that CIN frequently stems from compromised mitotic fidelity, we systematically interrogated the mitotic regulatory network. By integrating literature and Gene Ontology databases (GO: 0000278) [26], we defined a comprehensive set of 591 mitosis-related genes (Table S1). By analyzing gene expression data across four independent ccRCC datasets (TCGA-KIRC, GSE251905, GSE16449, and GSE252600), principal component analysis (PCA) revealed a profound transcriptomic divergence between tumor and normal tissues (Supplementary Fig. S1A), indicating a systemic dysregulation of the mitotic machinery. Differential expression analysis of these 591 mitosis-related genes in ccRCC vs. normal samples from the above datasets identified 47 significantly upregulated and seven downregulated genes in TCGA-KIRC ccRCC tumors (Fig. 1C). In the three additional datasets, 43 (GSE251905), 37 (GSE16449), and 71 (GSE252600) differentially expressed genes were identified. Unsupervised clustering analysis of these differentially expressed mitosis-associated genes grouped tumor and normal samples into two distinct clusters (Supplementary Fig. S1B). Integrated analysis across these datasets identified seven mitosis-related genes that were consistently and significantly upregulated in tumor samples (Fig. 1D).

To identify core drivers of metastasis, we employed LASSO-based variable selection. By evaluating the coefficient trajectories against model penalization (Fig. 1E) and optimizing the selection via 10-fold cross-validation (Fig. 1F), we narrowed our focus to a mathematically optimized 4 genes comprising SKA3, TUBA3D, DTL, and CEP55 (Supplementary Fig. S1C). Among these, SKA3 and CEP55 exhibited significant prognostic value (Supplementary Fig. S2A). We then analyzed the correlations of SKA3 and CEP55 with the previously reported CIN-related gene signature CA20 [27] and CIN25. Notably, while both genes correlated with these signatures, SKA3 presented a more robust association (Fig. 1G, H, and Supplementary Fig. S2B). We further performed immunohistochemical (IHC) assays to detect SKA3 expression in 35 paired normal and ccRCC tissues, which showed a higher level of SKA3 in tumors than in normal tissues (Fig. 1I). The analysis of TCGA data further confirmed higher expression of SKA3 in ccRCC (Supplementary Fig. S2C). Interestingly, SKA3 expression was elevated not only in high-AS (Fig. 1J) and metastatic ccRCC (Supplementary Fig. S2D), but also alongside the elevation of clinical stage (Supplementary Fig. S2E) and Fuhrman nuclear grade (Supplementary Fig. S2F). Further multivariable Cox regression analysis identified SKA3 as an independent prognostic factor after adjusting for key covariates, including age, sex, AJCC tumor stage, and *MKI67* (to control for active proliferation) (Supplementary Fig. S2G). Importantly, SKA3 maintained prognostic independence in two complementary analyses. First, an interaction analysis showed no significant interaction (*SKA3* × *MKI67*, *p* for interaction >0.05). Second, to explicitly isolate the proliferation-independent effect, we employed a residual analysis by regressing *SKA3* on *MKI67*. This consistently demonstrated that the *SKA3* residual component remained significantly associated with poor prognosis (HR = 1.71, *p* = 0.037; Supplementary Table S4). Collectively, these data suggested *SKA3* as a critical mediator of CIN and a potential biomarker for ccRCC aggressiveness.

### SKA3 promotes ccRCC proliferation, metastasis, and CIN

To evaluate the function of SKA3 in ccRCC, we first detected SKA3 expression across five renal cancer cell lines. Both RT-qPCR and western blotting assays showed relatively high endogenous SKA3 levels in 786-O and OS-RC-2 cells, while relatively low expression was observed in 769-P and Caki-1 cells (Supplementary Fig. S3A, B). Then, we designed and synthesized three siRNAs targeting SKA3, which showed that siRNA-1 and siRNA-3 exhibited superior knockdown efficiency (Supplementary Fig. S3C). Accordingly, shRNA-mediated stable SKA3-knockdown 786-O and OS-RC-2 cell lines were successfully generated (Supplementary Fig. S3D). Following SKA3 knockdown, cell proliferation was markedly suppressed (Supplementary Fig. S3E), and cell cycle progression was arrested at the G2/M phase (Supplementary Fig. S3F). Conversely, SKA3 overexpression in 769-P and Caki-1 cells (Supplementary Fig. S3G) significantly promoted cell growth (Supplementary Fig. S3H) and increased the proportion of cells in the S phase (Supplementary Fig. S3I). However, neither knockdown nor overexpression of SKA3 had any significant effect on the migratory and invasive capabilities of ccRCC cells in vitro (Supplementary Fig. S4A, B).

**Fig. 2:**
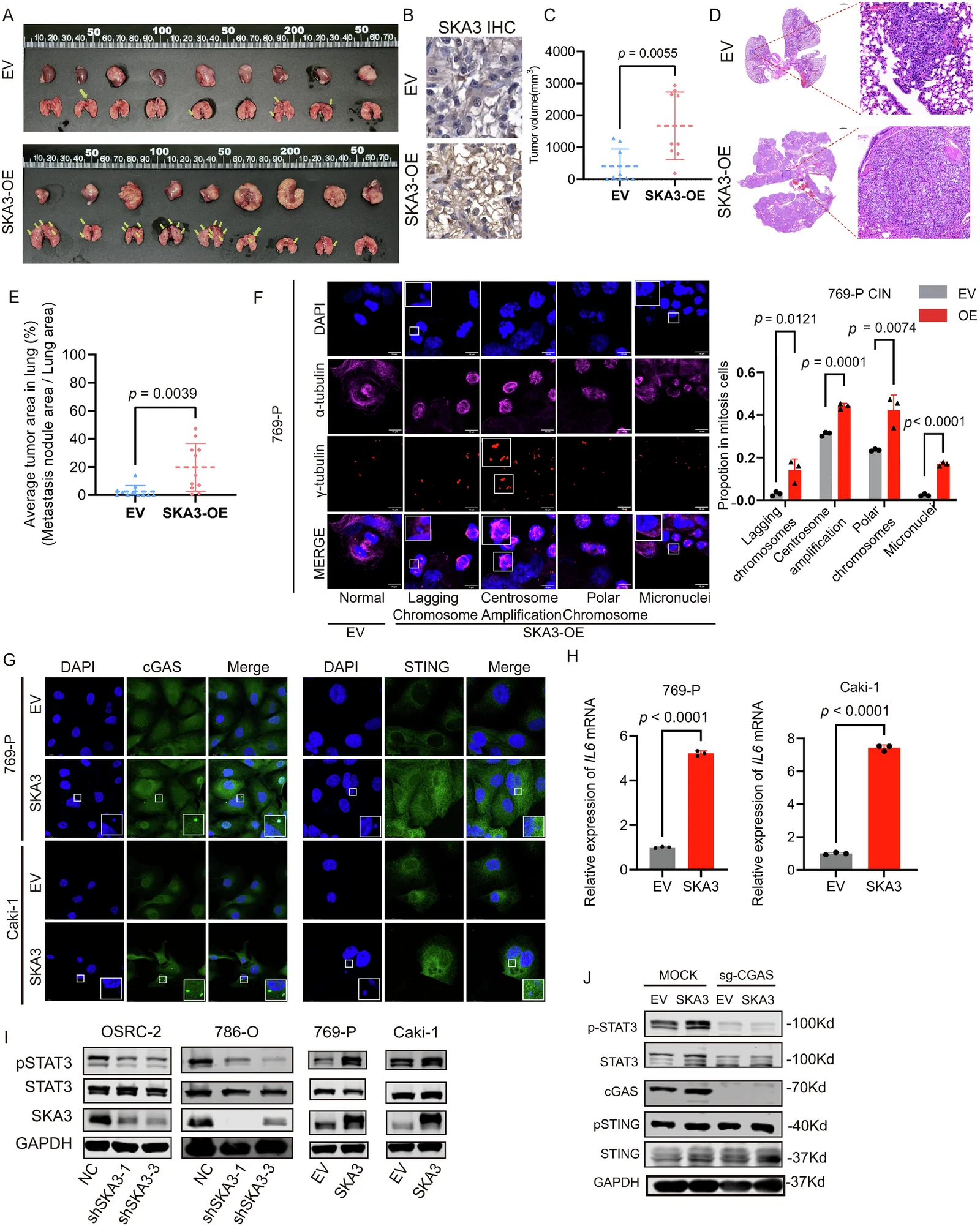
SKA3 promotes ccRCC proliferation, metastasis, and CIN. **A** Images of orthotopic renal xenograft tumors and lung metastasis (*n* = 9 per group). **B** Representative IHC staining of SKA3 in orthotopic xenograft tumor tissues. Scale bar = 50 μm. **C** Quantitative analysis of the volume of the renal orthotopic xenograft tumors (*n* = 9). **D** Representative images of H&E-stained lung tissues. Scale bar = 1 mm. **E** Quantitative analysis of the average tumor area of the metastatic lung nodules (*n* = 9). **F** Representative immunofluorescence image of α-tubulin (magenta), γ-tubulin (red), and DAPI (blue) in SKA3-overexpressing 769-P cells, showing normal mitosis and CIN (left). Scale bar = 10 μm. Proportion of mitotic M-phase cells exhibiting lagging chromosomes, centrosome amplification, chromosome misalignment (polar chromosome), and micronuclei formation (right). (*n* = 3 biological replicates, with at least 100 cells counted per replicate). **G** Immunofluorescence staining of cGAS (green, left) and STING (green, right) in SKA3-overexpressing 769-P and Caki-1 cells. Insets highlight cGAS-positive micronuclei. Scale bar = 20 μm. **H** RT-qPCR analysis of *IL6* mRNA expression in cells overexpressing SKA3. **I** Western blot analysis of STAT3 phosphorylation in ccRCC cells with SKA3 knockdown or overexpression. **J** Western blot analysis of STING and STAT3 phosphorylation levels in cGAS-knockout 769-P cells overexpressing SKA3. Data are presented as mean ± SD. The experiment was repeated *n* = 3 Independent times (biological replicates) (**F**–**J**), *n* = 9 biological replicates (**C**, **E**). Statistical analysis: independent Student’s *t*-test (**C**, **E**, **H**), multiple unpaired *t*-tests (**F**).

To test the effect of SKA3 on ccRCC in vivo, we constructed subcutaneous and renal orthotopic xenograft models. SKA3 knockdown significantly repressed tumor growth in the subcutaneous xenograft models (Supplementary Fig. S4C). Conversely, SKA3 overexpression markedly promoted primary tumor growth (Fig. 2A–C). Intriguingly, more lung metastatic foci were observed in the SKA3 overexpression group (Fig. 2D, E). These in vivo data suggest that SKA3 could promote ccRCC growth and distant metastasis. Speculatively, the discrepancy where SKA3 facilitates metastatic outgrowth in vivo despite having negligible impact on tumor cell motility in vitro suggests a potential role for CIN [3, 28].

Therefore, we performed immunofluorescence staining for α-tubulin (spindles), γ-tubulin (centrosomes), and DAPI (chromosomes) to systematically monitor mitotic progression. Ectopic SKA3 expression across four ccRCC cell lines increased the frequency of chromosomal lagging, abnormal centrosome amplification, misaligned/polar chromosomes, and micronuclei formation (Fig. 2F, Supplementary Fig. S5A, B). Time-lapse live-cell imaging of H2B-mCherry-infected cells revealed that SKA3 overexpression accelerated M-phase progression and induced abnormal centrosome amplification, lagging chromosomes, and micronuclei formation (Supplementary Fig. S5C, Movie S1–S3). These findings collectively suggest that SKA3 functions as an oncogene to drive CIN and to facilitate malignant progression.

Given that CIN-induced activation of the cGAS-STING cascade is an important mediator of tumor malignant progression in vivo [3, 4], we next explored whether SKA3-driven CIN activates the cGAS-STING cascade. Immunofluorescence assays revealed robust colocalization of SKA3-induced micronuclei with cGAS, thereby promoting STING perinuclear translocation (Fig. 2G). And overexpression of SKA3 (Supplementary Fig. S5D) upregulated *IL6* mRNA levels (Fig. 2H). Accordingly, SKA3 overexpression increased, while SKA3 knockdown decreased STAT3 phosphorylation level (Fig. 2I). Interestingly, the acute CIN-responsive marker *ISG15* was undetectable in 769-P and Caki-1 cells (Supplementary Fig. S5E), and *IRF3* levels were unaffected by SKA3 (Supplementary Fig. S5F), suggesting a non-canonical signaling output. To determine if SKA3 regulates STAT3 phosphorylation via the cGAS-STING pathway, we performed CRISPR-Cas9-mediated *CGAS* knockout. Importantly, *CGAS* knockout abolished SKA3-mediated upregulation of STAT3 phosphorylation (Fig. 2J). Taken together, these data suggest that the SKA3-CIN axis is intimately associated with the activation of a non-canonical cGAS-STING-STAT3 cascade, providing a potential molecular link to the aggressive phenotype of ccRCC.

### BAP1 mutation upregulates SKA3 expression in ccRCC

**Fig. 3:**
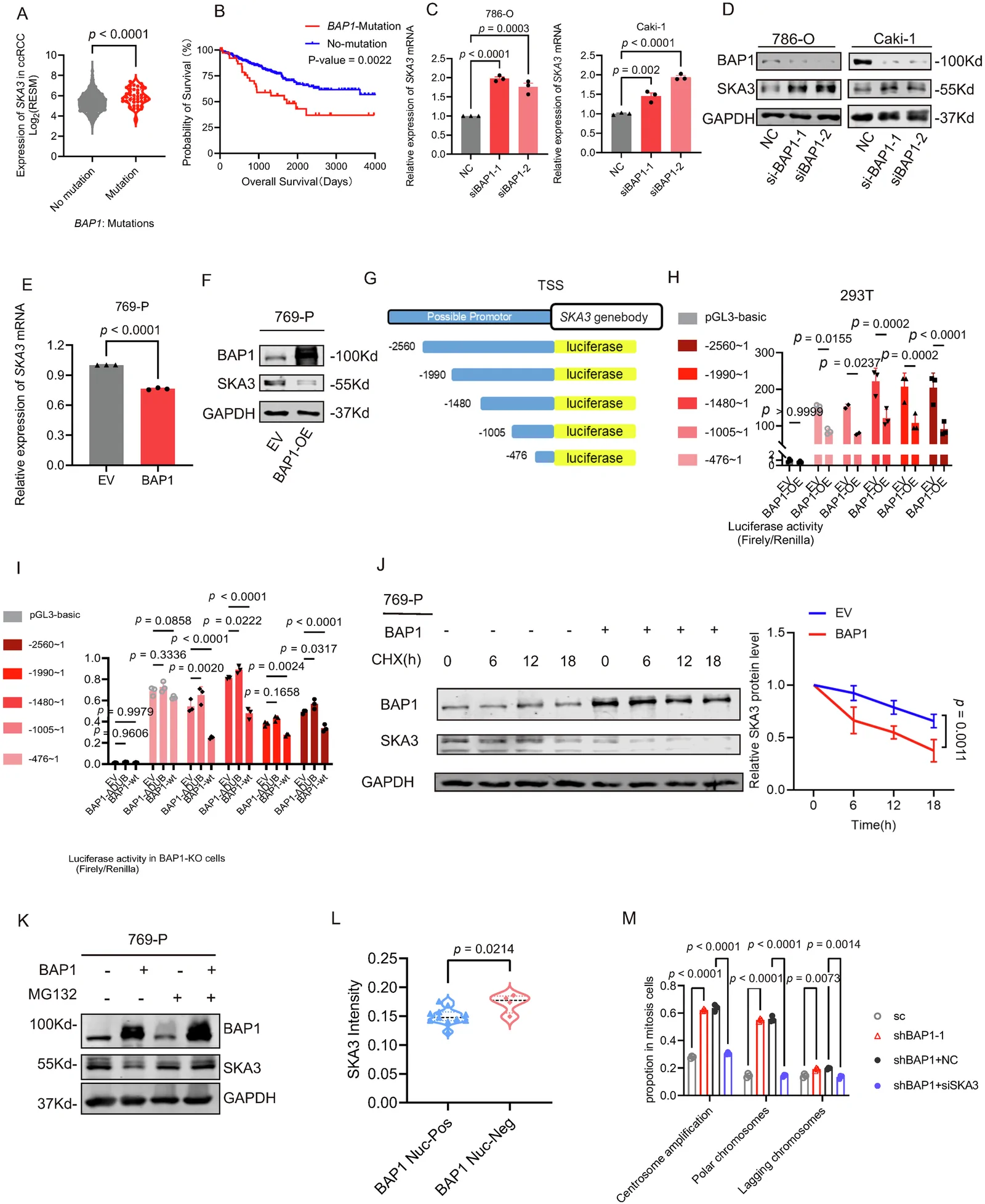
BAP1 mutation upregulates SKA3 expression in ccRCC. **A** Violin plots showing mRNA levels of *SKA3* in ccRCC samples stratified by *BAP1* somatic mutation status in the TCGA-KIRC cohort. **B** K–M analysis of OS in ccRCC patients with or without *BAP1* somatic mutations. **C** RT-qPCR analysis of *SKA3* mRNA levels in BAP1 knockdown 786-O and Caki-1 cells. **D** Western blot analysis of SKA3 protein level in BAP1 knockdown 786-O and Caki-1 cells. **E** RT-qPCR analysis of *SKA3* mRNA levels in BAP1 overexpressed 769-P cells. **F** Western blot analysis of SKA3 protein level in BAP1 overexpressed 769-P cells. **G** Schematic representation of truncated *SKA3* promoter-luciferase reporter constructs. **H** Dual-luciferase reporter assay evaluating the effect of BAP1 overexpression on the luciferase reporter activity of the *SKA3* promoter in 293T cells. **I** Dual-luciferase reporter assay evaluating the effect of wild-type and DUB-domain-deficient (ΔDUB) BAP1 overexpression on the luciferase reporter activity of the *SKA3* promoter in BAP1-knockout cells. **J** Protein stability analysis of SKA3 in BAP1-overexpressing 769-P cells treated with cycloheximide (CHX, 50 μg/ml) (left). Relative quantification of SKA3 expression levels over time (right). **K** Western blot analysis of SKA3 expression in BAP1-overexpressing 769-P cells treated with MG132 (20 μM). **L** Relative expression of SKA3 in BAP1 nuclear-negative versus BAP1 nuclear-positive samples (right) (*n* = 11 for BAP1 nuclear-positive; *n* = 4 for BAP1 nuclear-negative). **M** Proportion of mitotic M-phase cells exhibiting lagging chromosomes, centrosome amplification, or chromosome misalignment. Data are presented as mean ± SD; the experiment was repeated *n* = 3 independent times (biological replicates) (**C**–**F**, **H**–**K**, **M**). Statistical analysis: Mann–Whitney *U*-test (**A**), Log-rank test (**B**), independent Student’s *t*-test (**E**, **J**, **L**, **M**), one-way ANOVA (**C**), two-way ANOVA (**H**, **I**).

While our data establish SKA3 as a driver of ccRCC progression, the upstream regulatory mechanisms remain unclear. We first investigated SKA3 levels in ccRCC samples from the TCGA-KIRC cohort using the cBioportal database, stratified by the five most frequently mutated genes (*VHL*, *PBRM1*, *SETD2*, *BAP1*, and *KDM5C*). The results showed significantly elevated SKA3 levels in ccRCC samples with *SETD2* and *BAP1* mutations (Fig. 3A and Supplementary Fig. S6A). Further stratification into “driver” mutations (driver) and Variant of Uncertain Significance (VUS) revealed that SKA3 expression was the highest in samples with *BAP1* and *SETD2* “driver” mutations (Supplementary Fig. S6B). In addition, only the *BAP1* mutation was significantly associated with poor prognosis in TCGA-KIRC cohorts (Fig. 3B, Supplementary Fig. S6C), which was further validated in the TRACERx Renal cohort (Supplementary Fig. S6D) [29]. Notably, an integrated molecular analysis of over 100 patients indicated that SETD2 mutation is also associated with shorter disease-free survival [30]. Moreover, previous studies have also demonstrated that the loss of BAP1 and SETD2 enhances metastatic potential in mouse models of ccRCC, underscoring the functional relevance of these mutations in tumor progression [31, 32]. Notably, genomic profiling revealed that *BAP1* predominantly undergoes functional biallelic inactivation via concurrent somatic mutation and loss of heterozygosity (LOH) in ccRCC (Supplementary Fig. S6E). Given that the major somatic mutations of BAP1 and SETD2 in ccRCC are nonsense and missense mutations resulting in loss-of-function, we employed siRNA-mediated knockdown to mimic BAP1 or SETD2 deficiency in BAP1 and SETD2 wild-type ccRCC cell lines. Interestingly, SETD2 depletion failed to induce SKA3 upregulation (Supplementary Fig. S7A). In contrast, BAP1 knockdown (Supplementary Fig. S7B) markedly increased both mRNA (Fig. 3C) and protein (Fig. 3D and Supplementary Fig. S7C) levels of SKA3. Unexpectedly, overexpression of BAP1 slightly reduced SKA3 mRNA (Fig. 3E) but led to a profound reduction in SKA3 protein level (Fig. 3F).

To further verify that BAP1 regulates SKA3 expression, we performed dual-luciferase reporter gene assays. The results revealed that BAP1 suppresses the *SKA3* promoter activity, specifically targeting the region between −1008 and −476 bp relative to the transcription start site (TSS) (Fig. 3G, H and Supplementary Fig. S7D). As a deubiquitinase, BAP1 typically regulates the expression or activity of target proteins. We found that restoring wild-type BAP1, but not a deubiquitinase-domain-deficient mutant (ΔDUB) in *BAP1*-knockout cells, could suppress *SKA3* promoter activity (Fig. 3I). Additionally, cycloheximide (CHX) chase assays showed that ectopic expression of BAP1 significantly reduced SKA3 protein stability (Fig. 3J). Similarly, in BAP1-knockout cells, only reintroducing wild-type BAP1, but not the ΔDUB mutant, significantly destabilized the SKA3 protein (Supplementary Fig. S7E). This BAP1-induced downregulation was completely abolished by the proteasome inhibitor MG132 (Fig. 3K), confirming that BAP1 promotes the ubiquitin-proteasome-dependent degradation of SKA3.

Clinically, loss of nuclear BAP1 staining indicates mutated or functionally inactivated [33]. Therefore, we performed immunohistochemistry on 15 ccRCC tissues, which showed that SKA3 expression was significantly elevated in BAP1 nuclear-negative specimens compared to that in nuclear-positive specimens (Fig. 3L). To determine if this BAP1-SKA3 axis is functionally responsible for the observed CIN and growth advantages, we performed functional rescue experiments. BAP1 knockdown exacerbated CIN; however, knockdown of SKA3 almost entirely rescued these phenotypes (Fig. 3M, Supplementary Fig. S7F). Additionally, BAP1 knockdown enhanced the ccRCC cell proliferation, whereas SKA3 knockdown nearly abolished the effect of BAP1 on ccRCC cell proliferation (Supplementary Fig. S7G). Taken together, these findings suggest that BAP1 deficiency promotes malignant progression of ccRCC by unleashing SKA3-driven CIN and proliferation.

### BAP1 suppresses SKA3 expression by deubiquitinating NCOR1

As a core component of the polycomb repressive deubiquitinase (PR-DUB) complex, BAP1 classically regulates transcription by removing mono-ubiquitin from histone H2A at lysine 119 (H2AK119ub). To investigate whether BAP1 directly modulates the *SKA3* transcription through this canonical mechanism, we analyzed public H2AK119ub ChIP-seq data (GSE101987) and found that neither wild-type BAP1 nor its DUB-inactive mutant (C91A) reduced H2AK119ub levels at the *SKA3* promoter or gene body in BAP1-deficient ccRCC cells (Supplementary Fig. S8A). ChIP-qPCR confirmed that BAP1 knockdown did not increase H2AK119ub enrichment at the *SKA3* promoter region (Supplementary Fig. S8B). These findings suggest that BAP1 possibly influences SKA3 transcription through an indirect mechanism involving transcriptional co-repressors rather than direct PR-DUB activity.

**Fig. 4:**
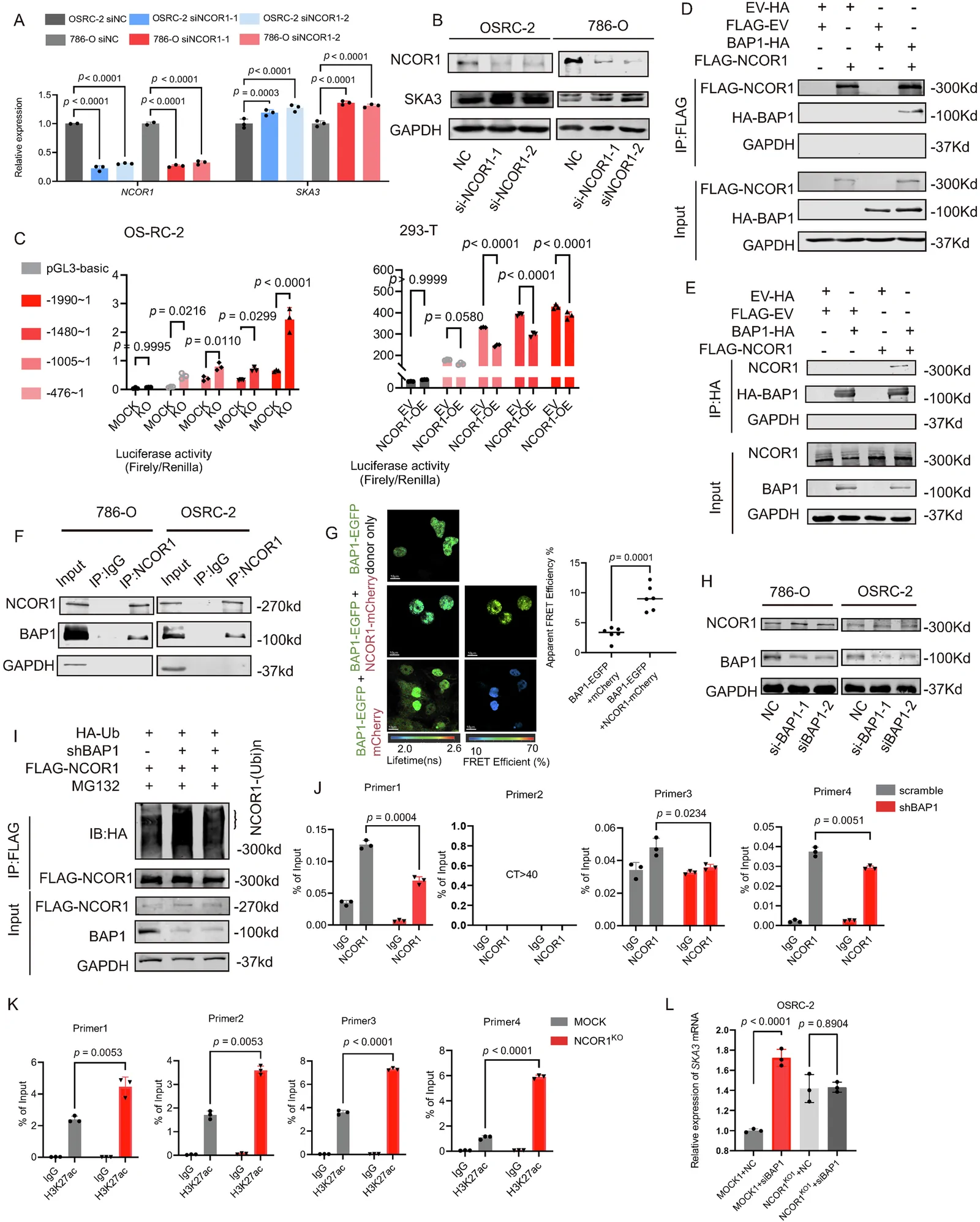
BAP1 Suppresses SKA3 Expression by Deubiquitinating NCOR1. **A** RT-qPCR analysis of *SKA3* mRNA levels in 786-O and OS-RC-2 cells with NCOR1 knockdown. **B** Western blot analysis of SKA3 protein expression in 786-O and OS-RC-2 cells with NCOR1 knockdown. **C** Dual-luciferase reporter assay evaluating *SKA3* promoter activity in NCOR1-knockout OS-RC-2 and NCOR1-overexpression HEK293T cells. **D**, **E** Reciprocal Co-IP assays in HEK293T cells transfected with HA-BAP1 and FLAG-NCOR1. **F** Endogenous Co-IP assay demonstrating the interaction between NCOR1 and BAP1. **G** FLIM-FRET analysis of EGFP fluorescence lifetime in cells expressing BAP1-EGFP (donor) alone or co-expressing with mCherry or NCOR1-mCherry (acceptor) (left). Quantitative analysis of apparent FRET efficiency is shown (right). **H** Western blot analysis of NCOR1 protein expression in 786-O and OS-RC-2 cells with BAP1 knockdown. **I** Ubiquitination assay showing that BAP1 knockdown enhances ubiquitin binding to FLAG-NCOR1. **J** ChIP-qPCR analysis of NCOR1 enrichment at distinct regions of the *SKA3* promoter in BAP1-knockdown OS-RC-2 cells. **K** ChIP-qPCR analysis of histone H3K27ac abundance at the *SKA3* promoter in OS-RC-2 NCOR1*-*KO cells. **L** RT-qPCR analysis of *SKA3* mRNA levels in NCOR1*-*KO OS-RC-2 cells with BAP1 knockdown. Data are presented as mean ± SD. The experiments were repeated *n* = 3 independent times (biological replicates) (**A**–**F**, **H**–**L**). The experiment was repeated *n* = 6 independent times (biological replicates), with at least 5 cells analyzed per experiment (**G**). Statistical analysis: independent Student’s *t*-test (**J**, **K**, **L**); one-way ANOVA (**A**), two-way ANOVA (**C**).

A previous study reported that BAP1 interacts with Nuclear Receptor Co-repressor 1 (*NCOR1*) to enhance the inhibitory effect on γ-globin transcription [34]. *NCOR1* is one of the core components of histone deacetylase (HDAC) complexes and mediates transcriptional repression by promoting histone deacetylation and forming repressive chromatin structures to impede basal transcription factors' binding to the promoter [35]. Therefore, we investigated whether BAP1 regulates SKA3 by interacting with NCOR1 in ccRCC. NCOR1 knockdown significantly upregulated SKA3 mRNA (Fig. 4A) and protein levels (Fig. 4B). Luciferase reporter assays confirmed that NCOR1 overexpression significantly inhibited, while NCOR1 knockout increased, *SKA3* promoter activity within the 476 bp region upstream of the TSS, which is consistent with the effects observed for BAP1 (Fig. 4C).

However, the mechanism by which BAP1 regulates NCOR1 remains unclear. Co-immunoprecipitation (Co-IP) assays using both exogenous and endogenous proteins confirmed the interaction between BAP1 and NCOR1 (Fig. 4D–F). Semi-exogenous pull-down assay using purified Flag-NCOR1 also confirmed the interaction between BAP1 and NCOR1 (Supplementary Fig. S9A). Moreover, immunofluorescence assays showed that BAP1 and NCOR1 colocalized in the nucleus (Supplementary Fig. S9B). To further confirm this interaction, we performed fluorescence lifetime imaging microscopy - Förster resonance energy transfer (FLIM-FRET) assays. The fluorescence lifetime of EGFP was significantly shortened in cells co-transfected with EGFP-BAP1 and NCOR1-mCherry, and the FRET efficiency was markedly higher than in cells co-expressing EGFP-BAP1 and mCherry alone (Fig. 4G), which suggests that BAP1 and NCOR1 are in close proximity (<10 nm). Notably, both BAP1 knockdown and knockout did not alter NCOR1 protein levels (Fig. 4H and Supplementary Fig. S9C), indicating that BAP1 regulated NCOR1 function rather than its stability.

Previous studies suggest that ubiquitination of NCOR1 impairs its chromatin binding [34, 35]. Therefore, we analyzed the effect of BAP1 on the ubiquitination level of NCOR1. Co-IP assays showed that the BAP1 knockdown markedly promoted NCOR1 ubiquitination (Fig. 4I), while ectopic expression of BAP1 markedly decreased the ubiquitination of NCOR1 (Supplementary Fig. S9D), whereas the DUB catalytic mutant BAP1 (C91A) was unable to reduce the ubiquitination of NCOR1 (Supplementary Fig. S9E). We further constructed BAP1 truncated variants according to the functional domain (Supplementary Fig. S9F) and found that BAP1 interacts with NCOR1 via the DUB catalytic UCH domain. Truncated variants of BAP1 and the catalytic mutant (C91A) failed to deubiquitinate NCOR1, which revealed that the intact full-length BAP1 protein is required for efficient NCOR1 deubiquitination (Supplementary Fig. S9G). Crucially, ChIP-qPCR demonstrated that BAP1 knockdown inhibited the enrichment of NCOR1 at the promoter region of SKA3 (Fig. 4J). Similarly, in BAP1‑knockout cells, only the restoration of wild‑type BAP1—but not the DUB‑domain-deficient mutant—enhanced the ability of NCOR1 binding to the promoter region of the *SKA3* gene (Supplementary Fig. S9H).

It has been reported that NCOR1 mediates the transcriptional repression of target genes, in part through its recruitment to histone deacetylase complexes, leading to histone deacetylation and chromatin condensation [36]. We then assessed H3K27ac levels following NCOR1 knockout. We found that NCOR1 knockout led to a significant increase in H3K27 acetylation (H3K27ac) (Supplementary Fig. S9I), specifically elevated H3K27ac enrichment at the *SKA3* promoter (Fig. 4K).

Next, we aimed to determine whether BAP1 repressed *SKA3* transcription through NCOR1. Intriguingly, the BAP1-mediated transcriptional suppression of *SKA3* was completely abolished in NCOR1-deficient cells (Fig. 4L and Supplementary Fig. S9J). Collectively, these results indicated that BAP1 facilitates the recruitment of NCOR1 to the *SKA3* promoter by deubiquitinating NCOR1, leading to the subsequent regulation of *SKA3* transcription.

### BAP1 promotes SKA3 degradation through regulating TRIM25 transcription

The ubiquitin-proteasome system is a fundamental pathway involved in protein degradation. Our observation that BAP1, a deubiquitinase typically associated with protein stabilization, paradoxically promotes SKA3 degradation suggested an indirect regulatory mechanism. To investigate whether BAP1 directly promotes SKA3 degradation, we performed immunofluorescence assays, which revealed no colocalization between BAP1 and SKA3 during both interphase and metaphase (Supplementary Fig. S10A). This suggested that BAP1 regulates SKA3 stability through an indirect mechanism.

**Fig. 5:**
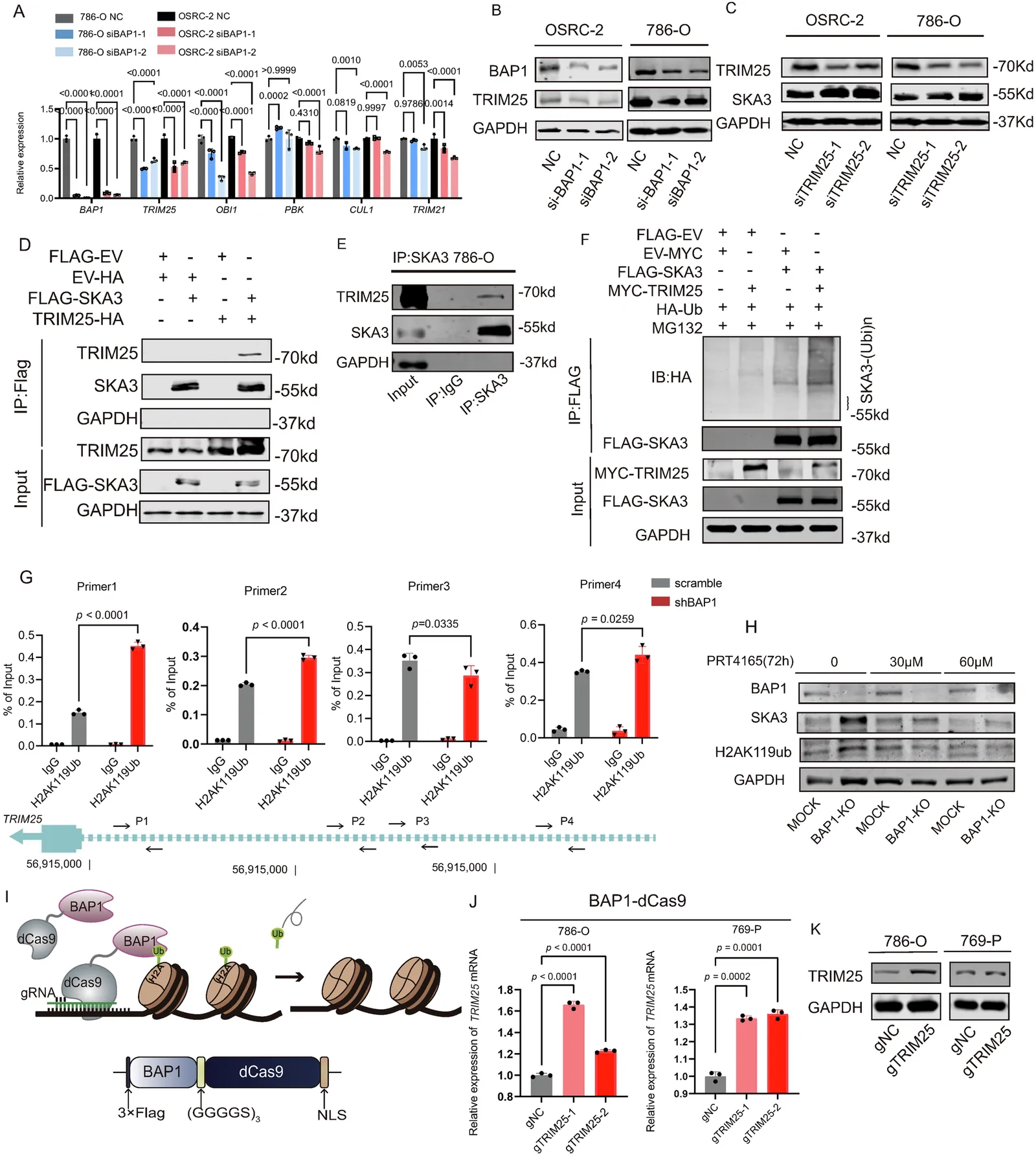
BAP1 promotes SKA3 degradation through regulating TRIM25 transcription. **A** RT-qPCR analysis of mRNA levels of ubiquitin-proteasome-related genes interacting with SKA3 in BAP1 knockdown 786-O and OS-RC-2 cells. **B** Western blot analysis of TRIM25 protein levels in OS-RC-2 and 786-O cells with BAP1 knockdown. **C** Western blot analysis of SKA3 protein expression in OS-RC-2 and 786-O cells with TRIM25 knockdown. **D** Co-IP assay showing FLAG-SKA3 and HA-TRIM25 interaction. **E** Co-IP assay showing endogenous SKA3 and TRIM25 interaction. **F** Co-IP assay demonstrating that TRIM25 enhances polyubiquitin-SKA3 binding. **G** ChIP-PCR analysis of histone H2AK119ub changes at *TRIM25* promoter regions in BAP1-knockdown 786-O cells. **H** Western blot analysis of SKA3 protein levels in BAP1 MOCK or KO OS-RC-2 cells treated with PRC1 inhibitor PRT4165. **I** Schematic diagram of dCas9-mediated BAP1-induced histone H2AK119 deubiquitination (top). The design of the BAP1-dCas9 fusion expression vector (bottom). **J** RT-qPCR analysis of *TRIM25* mRNA levels in BAP1-dCas9-ectopically expressed 786-O and 769-P cells transfected with specific sgRNAs. **K** Western blot analysis of TRIM25 protein levels in BAP1-dCas9-ectopically expressed 786-O and 769-P cells transfected with specific sgRNAs. Data are presented as mean ± SD. The experiment was repeated *n* = 3 independent times (biological replicates) (**A**–**H**, **J**–**K**). Statistical analysis: independent Student’s *t*-test (**G**), one-way ANOVA (**J**), two-way ANOVA (**A**).

We then screened ubiquitin-proteasome-associated proteins that interacted with SKA3 through a Co-IP assay combined with LC-MS/MS (Supplementary Fig. S10B). Among these proteins, the E3 ligase TRIM25 was identified as a potential ubiquitin-related protein that regulates SKA3. We found that BAP1 knockdown significantly reduced TRIM25 expression (Fig. 5A, B), while TRIM25 knockdown markedly increased the protein level of SKA3 (Fig. 5C). Subsequent Co-IP assay confirmed that TRIM25 interacts with SKA3 (Fig. 5D, E and Supplementary Fig. S10C). Ubiquitination assays showed that TRIM25 promotes the poly-ubiquitination of SKA3 (Fig. 5F, Supplementary Fig. S10D), establishing TRIM25 as a functional E3 ligase for SKA3.

We next investigated whether BAP1 modulates TRIM25 expression through its canonical PR-DUB activity. Analysis of H2AK119ub ChIP-seq data from the GEO dataset (GSE101987) showed that the wild-type BAP1, but not its DUB-inactive mutant, significantly reduced H2AK119ub levels at the *TRIM25* genomic locus in ccRCC cells (Supplementary Fig. S10E). Our ChIP-qPCR was consistent with this, showing that BAP1 knockdown increased H2AK119ub occupancy at the *TRIM25* promoter (Fig. 5G). In BAP1-knockout cells, wild-type BAP1 re-expression reduced H2AK119ub occupancy at the *TRIM25* promoter, while the BAP1ΔDUB mutant had no effect (Supplementary Fig. S10F). Consistently, TRIM25 mRNA levels were also upregulated by wild-type BAP1 re-expression (Supplementary Fig. S10G), which demonstrates that BAP1 regulates histone ubiquitination at the TRIM25 locus in a catalysis-dependent manner. In addition, we found that BAP1 did not affect the protein stability of SKA3 in cells with TRIM25 knockdown (Supplementary Fig. S10H). We also applied PRT4165, an inhibitor of the PRC1 complex, to specifically inhibit the mono-ubiquitination of H2AK119. The results showed that BAP1 knockout increased SKA3 expression; however, the protein level of SKA3 was significantly reduced by the treatment of PRT4165 (Fig. 5H).

To prove that BAP1-mediated deubiquitination specifically at the TRIM25 promoter is sufficient to drive TRIM25 expression, we constructed a BAP1-dCas9 fusion protein utilizing the catalytically inactive Cas9 protein (dCas9) to achieve locus-specific modulation of histone H2AK119 deubiquitination at the target genomic site (Fig. 5I). Immunoblotting showed a consistent molecular weight with the expected BAP1-dCas9 fusion protein at ~250 kDa (Supplementary Fig. S11A). Immunofluorescence demonstrated a significant colocalization between FLAG-tagged fusion proteins and H2AK119ub signals (Supplementary Fig. S11B). ChIP-qPCR confirmed that by using site-specific sgRNAs, the BAP1-dCas9 system markedly reduced H2AK119ub occupancy specifically at the *TRIM25* promoter region (Supplementary Fig. S11C). Further analysis showed that TRIM25 mRNA (Fig. 5J) and protein (Fig. 5K) levels were increased by the BAP1-dCas9 fusion protein. In addition, a positive correlation between BAP1 and TRIM25 mRNA expression was observed in the TCGA-KIRC cohort (Supplementary Fig. S11D). Collectively, these data demonstrate that BAP1 facilitates the proteasomal degradation of SKA3 by activating TRIM25 transcription.

### Impact of SKA3 and BAP1 status on therapeutic response in ccRCC

**Fig. 6:**
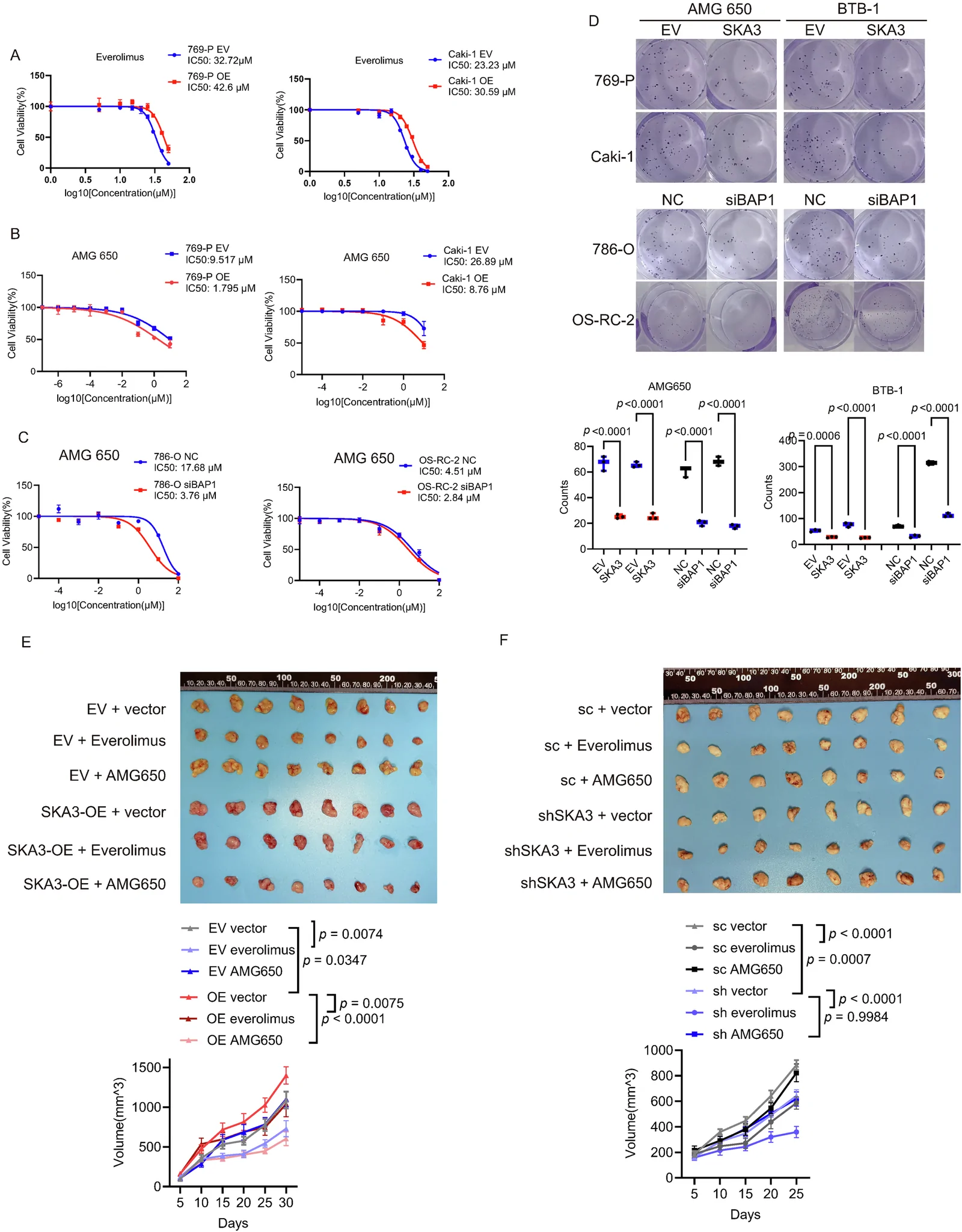
Impact of SKA3 and BAP1 status on therapeutic response in ccRCC. **A** Dose-response curves of 769-P and Caki-1 cell lines for the empty vector (EV) and SKA3-OE groups to everolimus (96 h). **B** Dose-response curves for the 769-P and Caki-1 cell lines of EV and SKA3-OE groups to KIF18A inhibitor AMG650 (144 h). **C** Dose-response curves of 786-O cell lines for the non-targeting control (NC) or siBAP1 groups to KIF18A inhibitor AMG650 (144 h). **D** Colony formation assay of the cells overexpressing SKA3 or BAP1 knockdown treated with KIF18A inhibitors AMG650 (left) and BTB1 (right). **E** AMG650 and everolimus efficacy analysis in SKA3-OE OS-RC-2 cell-derived xenograft tumor models. Mice were orally administered (*p.o*.) vehicle, AMG650 (30 mg/kg), and everolimus (2.5 mg/kg) daily for 20 days. Graphs show tumor volume measurements as mean ± SD. versus time (d) (*n* = 8 mice per group). **F** AMG650 and everolimus efficacy analysis in SKA3-knockdown CDX tumor models. Mice were orally administered (*p.o*.) vehicle, AMG650 (30 mg/kg), and everolimus (2.5 mg/kg) daily for 20 days. Graphs show tumor volume measurements as mean ± SD. versus time (d) (*n* = 8 mice per group). Data are presented as mean ± SD. The experiment was repeated *n* = 3 independent times (biological replicates) (**A**–**D**), *n* = 8 biological replicates (mice) per group. Statistical analysis: independent Student’s *t*-test (**A**–**C**), one-way ANOVA (**D**) and two-way ANOVA with Tukey’s post hoc test (**E**, **F**).

To evaluate the clinical implications of SKA3-mediated CIN in ccRCC, we assessed the therapeutic sensitivity of ccRCC cells to frontline targeted agents. While SKA3 overexpression did not significantly alter cellular responses to tyrosine kinase inhibitors (TKIs) such as sunitinib or pazopanib (Supplementary Fig. S12A, B), it conferred resistance to the mTOR inhibitor everolimus in both 769-P and Caki-1 cell lines (Fig. 6A), suggesting that SKA3-driven CIN may serve as a potential contributor to everolimus insensitivity in this setting.

Targeting the spindle assembly checkpoint (SAC) or KIF18A components has emerged as a strategic vulnerability for aneuploid tumor cells [37–39]. We therefore investigated whether ccRCC cells with SKA3 overexpression showed an increased susceptibility to SAC or KIF18A inhibition. Although we observed no significant enhancement in sensitivity to the AZ3146 (one inhibitor of SAC component MPS1) upon SKA3 overexpression (Supplementary Fig. S12C), targeting KIF18A (BTB1 or AMG650) exhibited markedly enhanced potency in cells with either SKA3 overexpression (Fig. 6B and Supplementary Fig. S12D) or BAP1 depletion (Fig. 6C). These results were further confirmed by clone formation assay (Fig. 6D). We further validated this therapeutic strategy using mouse xenograft models. In subcutaneous models, the efficacy of everolimus treatment was attenuated in SKA3-overexpressing tumors, whereas the KIF18A inhibitor potently suppressed tumor growth (Fig. 6E). Conversely, in SKA3-knockdown tumors, KIF18A inhibitor showed limited efficacy compared to the robust response seen with everolimus (Fig. 6F). These in vitro and in vivo observations collectively highlight the SKA3-CIN axis as a preliminary but promising biomarker for predicting targeted therapy responses in ccRCC.

To investigate if targeting KIF18A exacerbates genomic damage in CIN-high tumor cells, we assessed DNA damage levels under relevant genetic conditions. Comet assays and γH2A.X immunostaining indicated that BAP1 deficiency alone induced a modest increase in DNA damage (Supplementary Fig. S12E, F). Metaphase spread assays revealed that BAP1-knockout cells exhibited more chromosomal aberrations (Supplementary Fig. S12G). In a comparative analysis of 10 BAP1 nuclear-negative and 10 BAP1 nuclear-positive ccRCC patient samples revealed that tumors lacking nuclear BAP1 expression exhibited a higher frequency of γH2A.X-positive and cGAS-positive cells than BAP1 nuclear-positive tumors (Supplementary Fig. S13A), suggesting increased intrinsic genomic instability in BAP1-mutant ccRCC. Crucially, the KIF18A inhibitor significantly exacerbated DNA damage specifically in SKA3-overexpressing cells (Supplementary Fig. S13B). Collectively, these findings indicate that ccRCC tumors with BAP1 mutation or high SKA3 expression are sensitive to the KIF18A inhibitor.

## Discussion

**Fig. 7:**
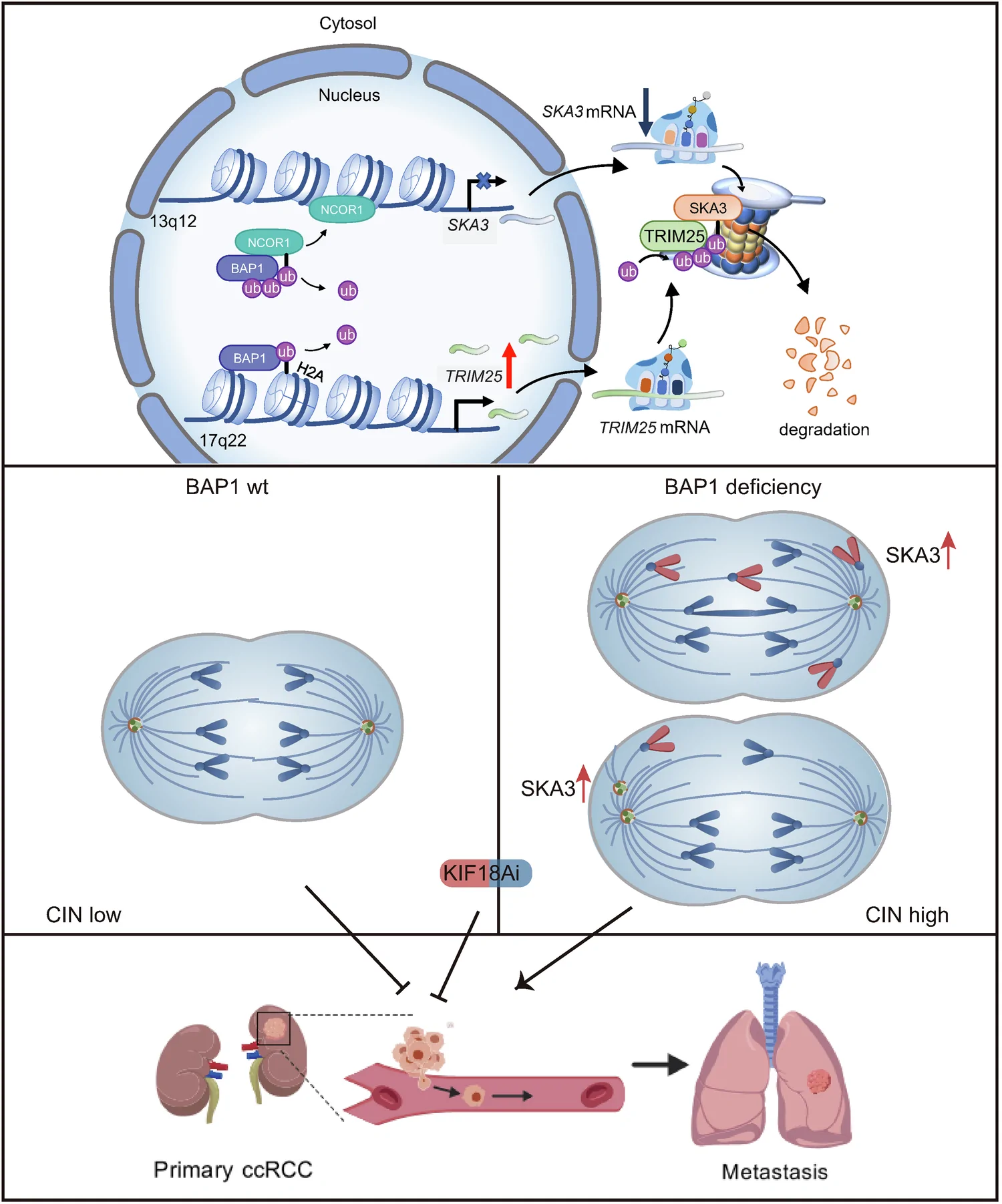
BAP1 deficiency-induced SKA3 promotes ccRCC malignant progression by regulating CIN. BAP1 regulates SKA3 expression through dual-regulatory mechanisms: (1) Transcriptional repression—BAP1 deubiquitinates NCOR1 to repress *SKA3* transcription; (2) Post-translational modification—BAP1 enhances TRIM25 expression by deubiquitinating H2AK119 at the *TRIM25* promoter, enabling TRIM25-mediated SKA3 ubiquitination and subsequent degradation. Loss of BAP1 collapses both pathways, elevating SKA3 levels, which induces CIN in ccRCC, ultimately driving metastasis and poor prognosis.

CIN is a hallmark of cancer that drives tumor heterogeneity and malignant progression [3, 40, 41]. Although aberrant mitotic regulation is a major contributor to CIN, the tissue-specific molecular mechanisms underlying this process remain poorly defined. Here, we show that SKA3 is overexpressed in ccRCC and promotes malignant phenotypes by inducing CIN and possibly activating non-canonical cGAS-STING signaling. Furthermore, we demonstrate that BAP1, an epigenetic regulator frequently mutated in ccRCC, suppresses SKA3 expression through dual pathways: NCOR1-mediated transcriptional repression and TRIM25-mediated proteasomal degradation. Consequently, loss of BAP1 leads to aberrant accumulation of SKA3, thereby driving CIN and accelerating tumor progression (Fig. 7).

The SKA complex ensures mitotic fidelity by stabilizing kinetochore-microtubule attachments under mechanical tension [18, 19, 42–44]. In normal cells, both the expression levels and phosphorylation states of the SKA components are precisely regulated. However, SKA subunits are overexpressed in multiple cancers and associated with poor prognosis, although their tumor-promoting roles have largely been attributed to non-mitotic functions in previous studies [45, 46]. For example, *SKA3* has been reported to function as an oncogene in cholangiocarcinoma through stabilizing HIF1α protein [22]. Nevertheless, the specific role of *SKA3* in ccRCC has remained unclear. In this study, we identify a novel oncogenic function of *SKA3* in ccRCC, revealing its critical contribution to CIN. This instability, in turn, activates the cGAS-STING-IL6 signaling axis, which promotes metastatic progression in ccRCC.

In contrast to the role in other cancer contexts, SKA3 promotes ccRCC progression through the induction of micronuclei formation. The fragile micronuclei envelopes are susceptible to rupture via p62/SQSTM1-dependent autophagy and mitochondria-derived reactive oxygen species (ROS) [47, 48], which exposes genomic DNA to the cytosol and activates the cGAS-STING signaling cascade. The cGAS-STING signaling axis has emerged as a critical mediator linking CIN to tumor progression. While STING activation typically induces STAT1-mediated interferon responses, our data implicate that SKA3 overexpression preferentially activates the IL6-STAT3 axis in a cGAS-dependent manner, as evidenced by the unchanged levels of classical interferon signaling (ISG15 and IRF3).

While SKA3 is upregulated in various tumors [45, 49], the specific regulatory machinery responsible for maintaining SKA3 homeostasis had not been defined. In this study, we found BAP1 regulates SKA3 expression by acting at both transcriptional and post-translational levels. Specifically, BAP1 facilitates the recruitment of the co-repressor NCOR1 to the *SKA3* promoter, thereby silencing *SKA3* transcription. In parallel, BAP1 functions as a transcriptional activator for the E3 ligase TRIM25 by reducing H2AK119ub levels at the *TRIM25* promoter, ensuring the continuous proteasomal degradation of SKA3 protein. The BAP1-SKA3 regulatory axis represents a critical safeguard for maintaining genomic stability. Consequently, BAP1 deficiency—a frequent event in ccRCC—leads to aberrant SKA3 accumulation, disrupting mitotic fidelity and driving CIN. Intriguingly, although endogenous BAP1 typically exerts a tempered, fractional effect on its downstream targets—as seen by the moderate 50% reduction in TRIM25 mRNA and partial NCOR1 ubiquitination changes—our experimental siRNA or CRISPR tools inflict an abrupt and near-complete ablation of these individual components. The expression of this mitosis-related gene is precisely regulated; disrupting either its transcriptional “intake valve” (NCOR1) or its proteolytic “drain pipe” (TRIM25) is possibly sufficient to overwhelm the protein’s kinetic balance, causing a rapid accumulation that mimics complete BAP1 loss. Furthermore, this overlapping phenotype is likely reinforced by a biological “ceiling effect” of genomic instability, where the high CIN levels observed in single-knockout models may already represent the tolerable threshold for viable ccRCC cells.

Notably, BAP1 loss may concurrently perturb additional cell cycle regulators, which may explain why SKA3 inhibition only partially rescues the phenotypes associated with BAP1 depletion. Nonetheless, aberrant upregulation of SKA3 levels resulting from BAP1 depletion is indeed an important driver of increased CIN levels, providing a molecular basis for the poor prognosis observed in ccRCC patients harboring BAP1 mutations.

However, the clinical consequences of BAP1 mutations appear paradoxically context-dependent. While somatic BAP1 loss in ccRCC is associated with high-grade progression and metastasis, germline mutations often predispose individuals to more indolent forms of mesothelioma [50]. We hypothesize this discrepancy stems from the distinct genomic landscapes in which these mutations occur [51]. In ccRCC, somatic BAP1 loss frequently arises within a “primed” background of chromosome 3p loss. By stringently restricting our analyses to ‘Oncogenic’ variants annotated by OncoKB/ClinVar databases and filtering out variants of uncertain significance (VUS), we confirmed that these pathogenic alterations predominantly undergo functional biallelic inactivation via the classic ‘two-hit’ mechanism—specifically, a somatic mutation coupled with LOH. This complete biallelic ablation within a 3p-deficient environment characteristic of ccRCC directly leads to the downstream SKA3 upregulation and induction of CIN. By contrast, in germline cases, systemic haploinsufficiency may favor a more prolonged evolutionary trajectory. This path likely involves lower mutational “noise” compared to the explosive genomic instability observed in asbestos-induced sporadic cases or in the 3p-deficient environment characteristic of ccRCC.

In conclusion, dysregulation of the BAP1-mediated SKA3 homeostasis represents a key driver of CIN in ccRCC. BAP1-deficient tumors exhibit SKA3 overexpression, which is related to increased metastatic potential and poorer clinical outcomes. Moreover, high SKA3 levels, potentially in combination with other mitosis-related genes, may serve as predictive biomarkers for metastasis risk in ccRCC. Although these preliminary insights offer potential clinical utility in risk stratification, future prospective studies are needed to fully validate whether targeting the SKA3-CIN axis, such as KIF18A inhibition, can effectively overcome CIN-driven disease progression.

## Supplementary information

Supplementary Figure S1

Supplementary Figure S2

Supplementary Figure S3

Supplementary Figure S4

Supplementary Figure S5

Supplementary Figure S6

Supplementary Figure S7

Supplementary Figure S8

Supplementary Figure S9

Supplementary Figure S10

Supplementary Figure S11

Supplementary Figure S12

Supplementary Figure S13

Supporting Information

Table S1

Movie S1

Movie S2

Movie S3

Full and uncropped western blots

## Data Availability

All stable/unique reagents generated in this study are available from the lead contact with a completed materials transfer agreement. The public microarray/RNA-seq data analyzed in this study were obtained from the TCGA database and Gene Expression Omnibus (GEO) under accession numbers GSE251905, GSE252600, and GSE16449. The ChIP-seq data were obtained from GSE101987. Additional raw data, resources, and reagents are available upon reasonable request to the corresponding author. All data analyses were performed using existing software packages as described. This paper does not report original code.
