# Supplementary material for: BAP1 deficiency-induced SKA3 promotes clear cell renal cell carcinoma malignant progression by regulating chromosomal instability
Source: Cell Death Dis. 2026 Jul 15;17(1):874. doi: 10.1038/s41419-026-09076-3 (PMC13649664; doi:10.1038/s41419-026-09076-3)

Figure_2I

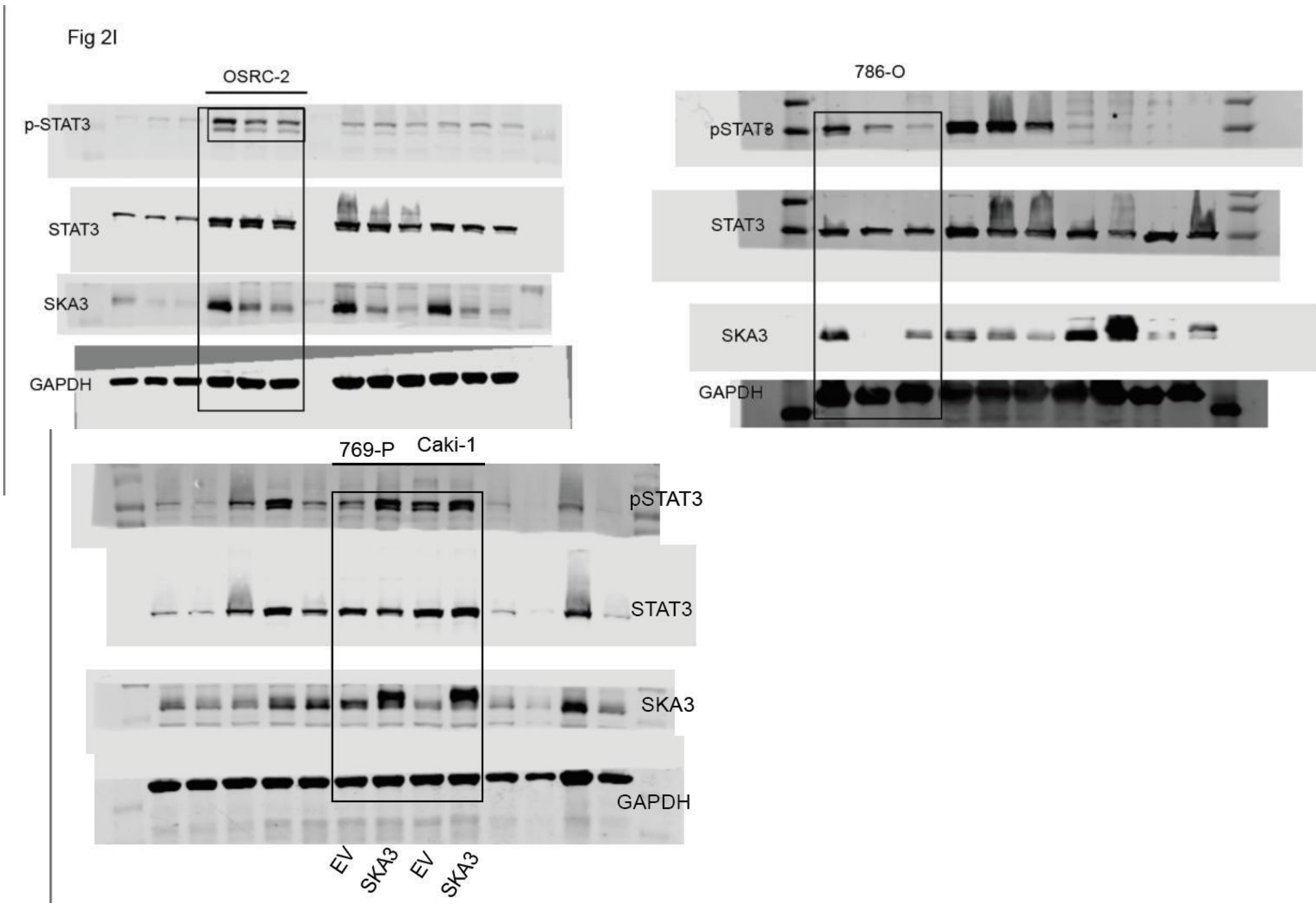

Fig 2I
OSRC-2
p-STAT3
STAT3
SKA3
GAPDH
786-O
pSTAT3
STAT3
SKA3
GAPDH
769-P
Caki-1
pSTAT3
STAT3
SKA3
GAPDH
EV
SKA3
EV
SKA3

Fig 2J

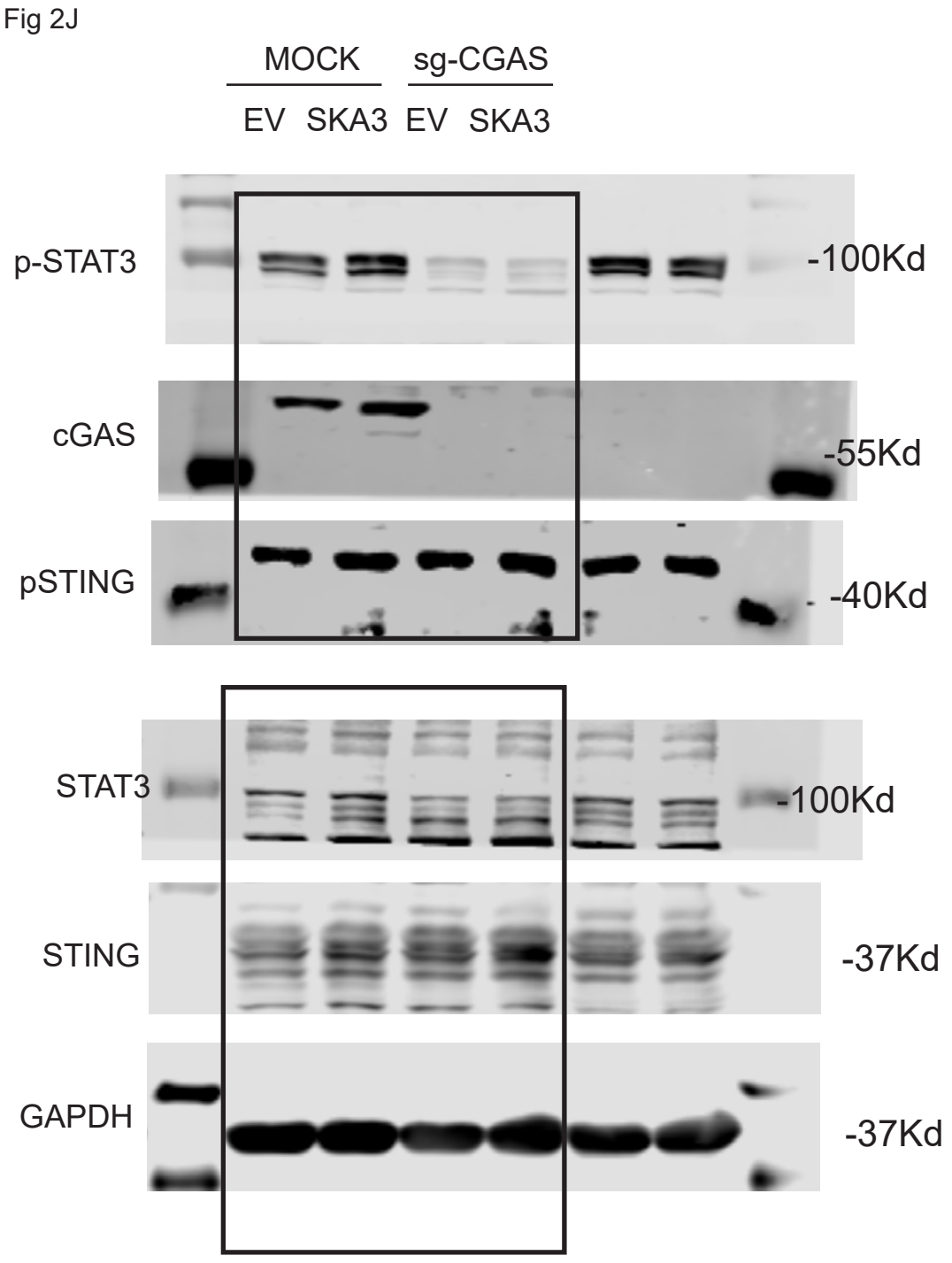
MOCK
sg-CGAS
EV
SKA3
EV
SKA3
p-STAT3
-100Kd
cGAS
-55Kd
pSTING
-40Kd
STAT3
-100Kd
STING
-37Kd
GAPDH
-37Kd

Figure_3D

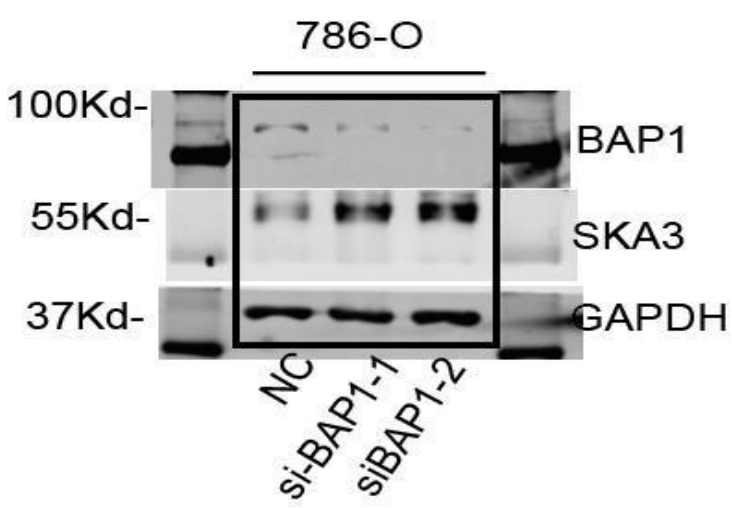

786-O
100Kd-
55Kd-
37Kd-
BAP1
SKA3
GAPDH
NC
si-BAP1-1
siBAP1-2

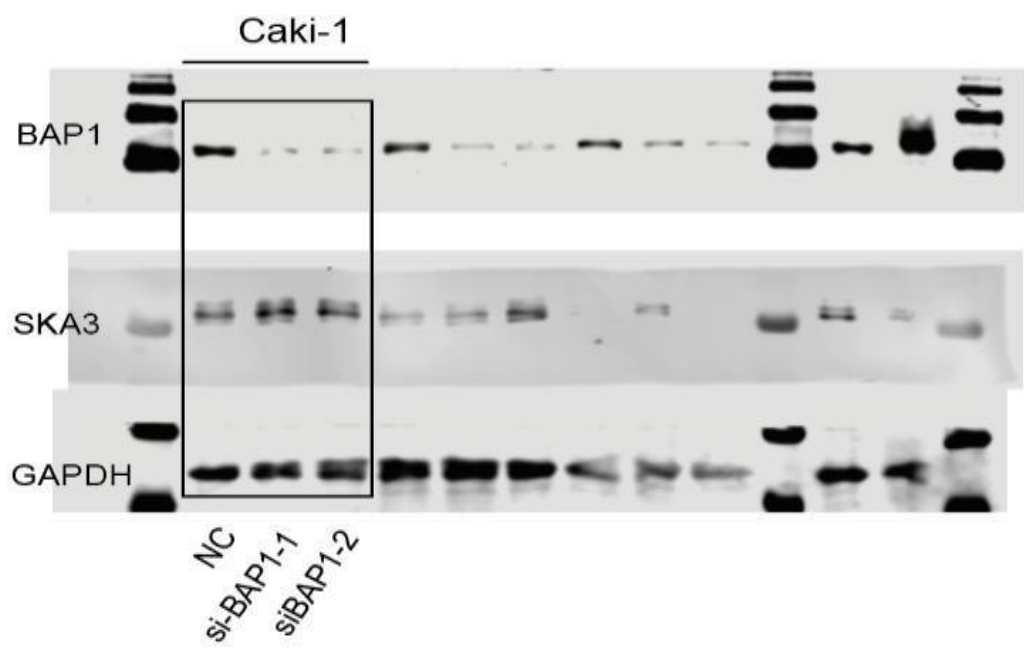

Caki-1
BAP1
SKA3
GAPDH
NC
si-BAP1-1
siBAP1-2

Figure_3F

Fig 3F

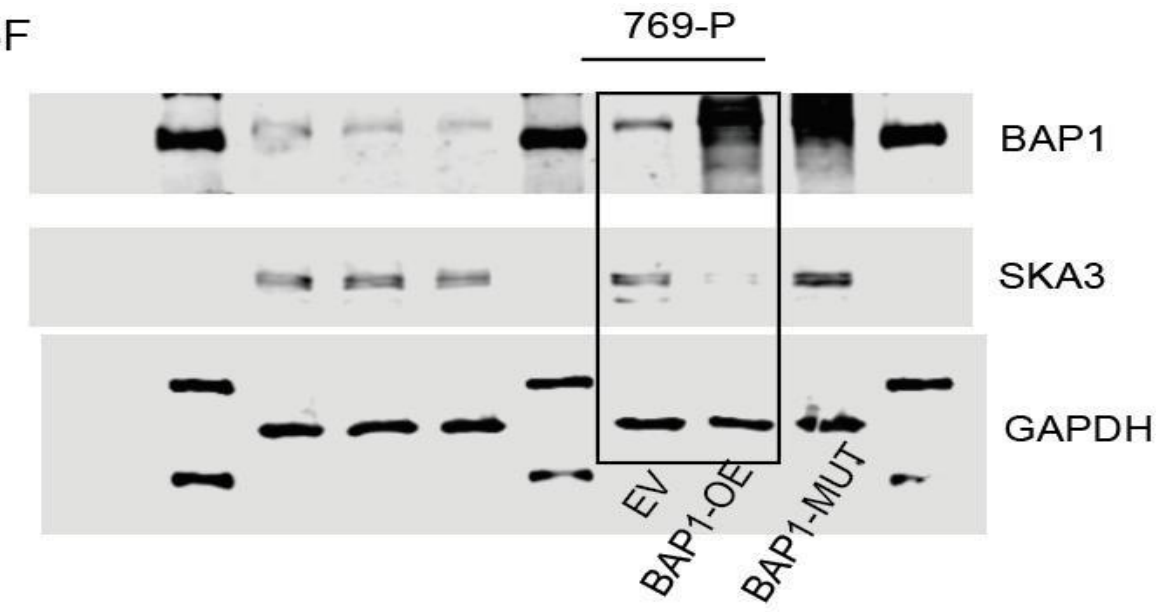

Figure_3J

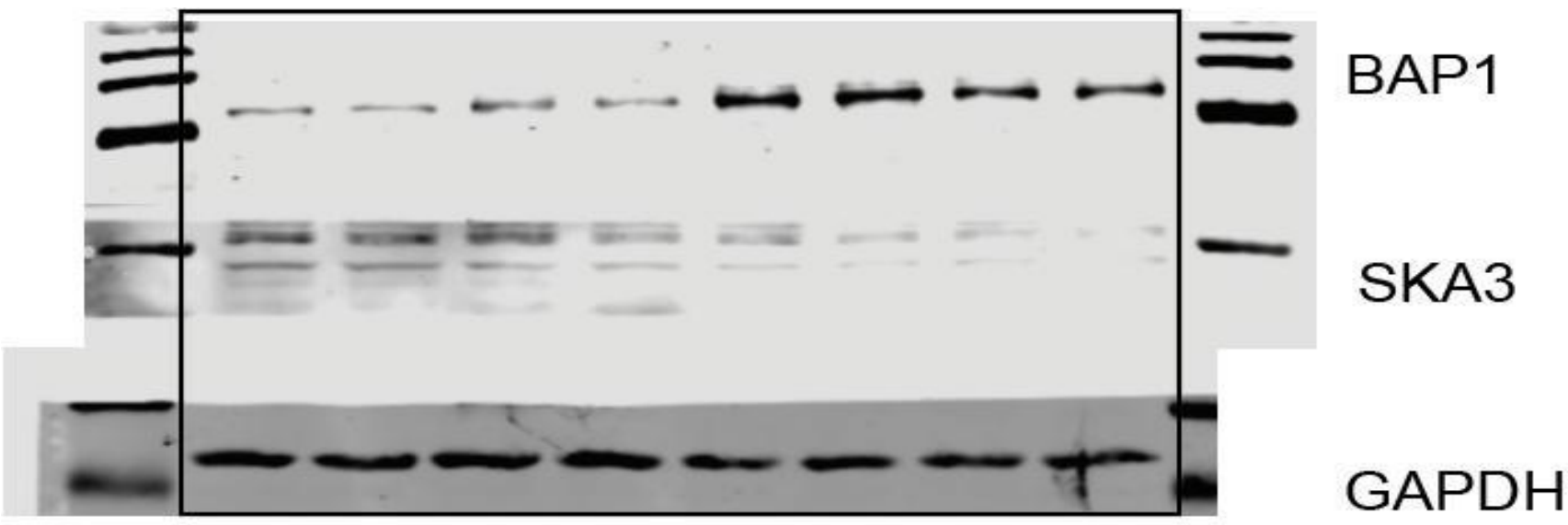

	EV			BAP1		
0	1.00	1.00	1.00	1.00	1.00	1.00
6	0.96	0.84	0.96	0.56	0.80	0.63
12	0.74	0.86	0.76	0.52	0.62	0.51
18	0.59	0.72	0.66	0.35	0.49	0.28

Figure_3K

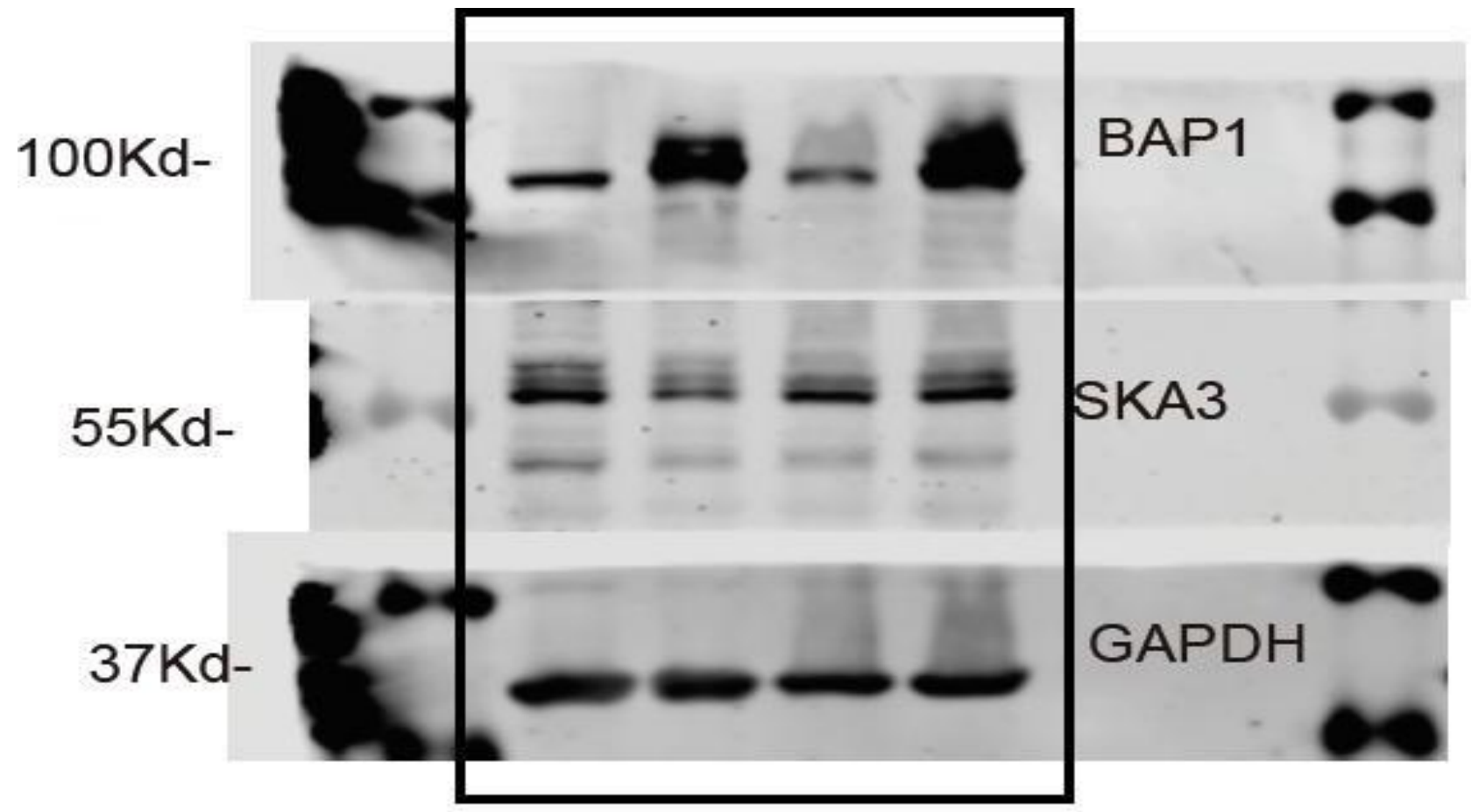

Figure_4B

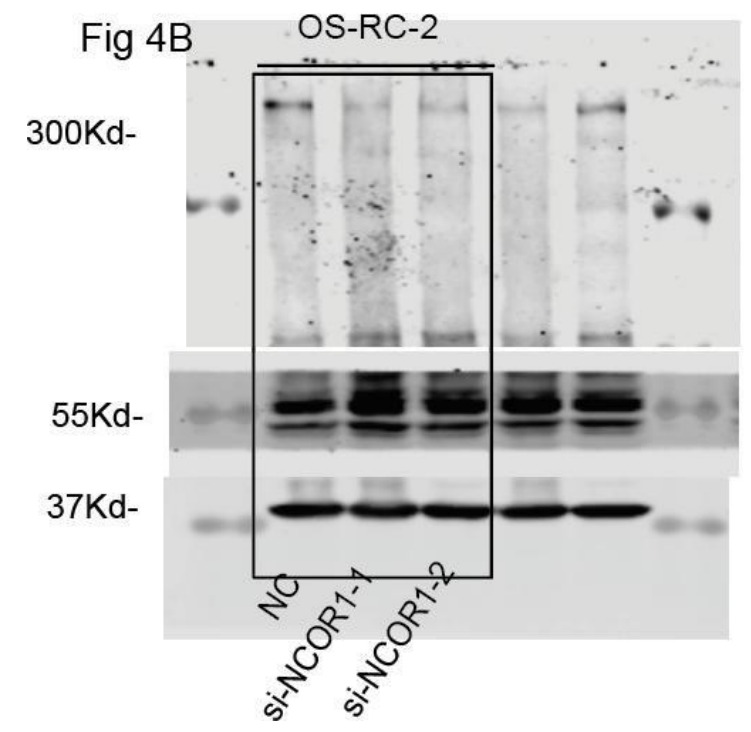

Fig 4B
OS-RC-2
300Kd-
55Kd-
37Kd-
NC
si-NCOR1-1
si-NCOR1-2

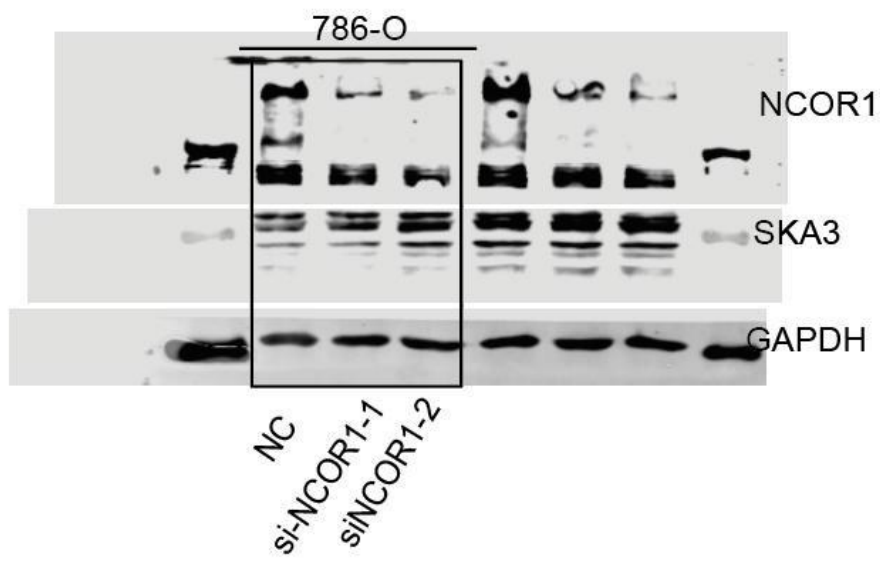

786-O
NCOR1
SKA3
GAPDH
NC
si-NCOR1-1
siNCOR1-2

Figure_4D

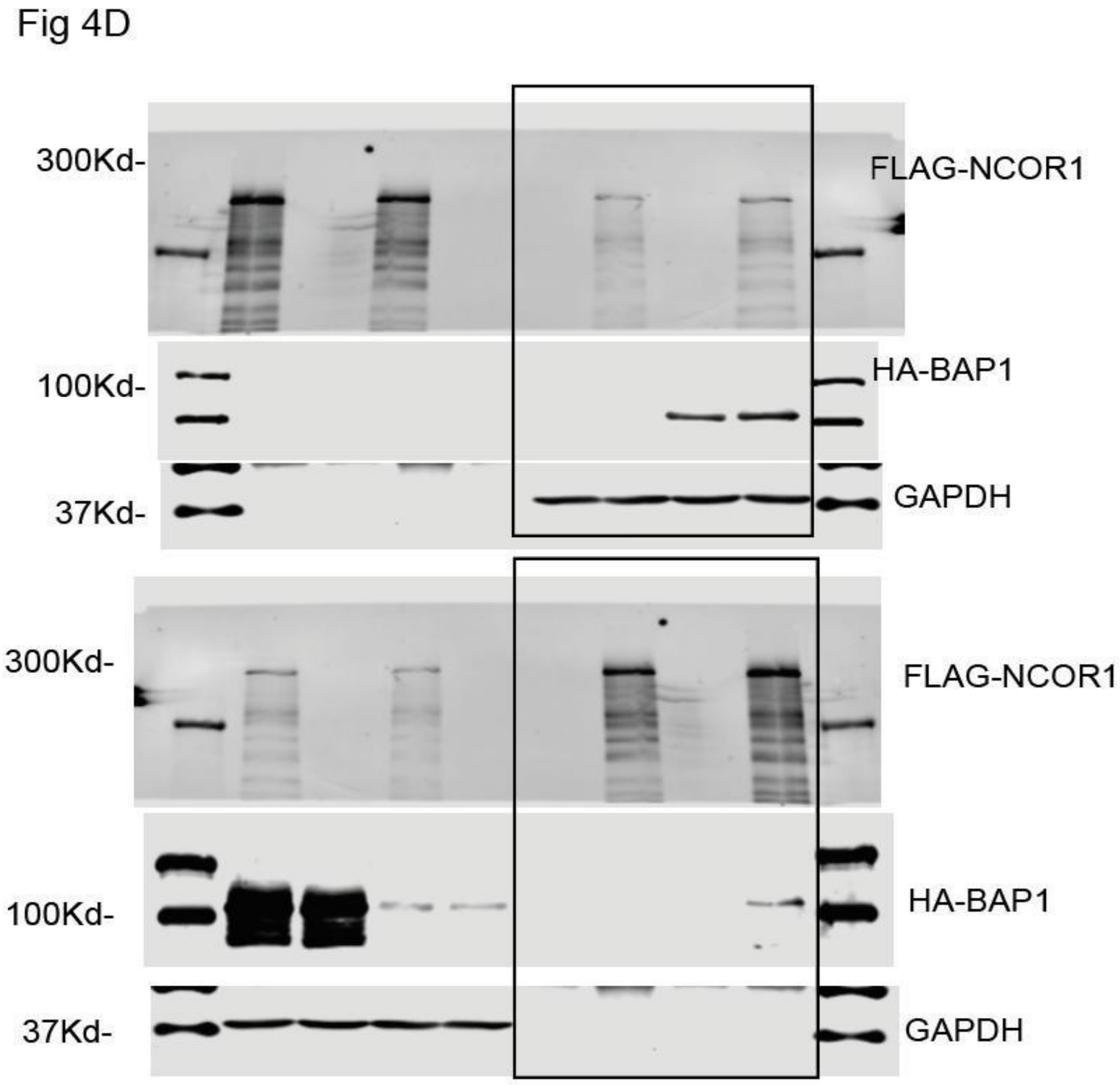
Fig 4D
300Kd-
FLAG-NCOR1
100Kd-
HA-BAP1
37Kd-
GAPDH
300Kd-
FLAG-NCOR1
100Kd-
HA-BAP1
37Kd-
GAPDH

Figure_4E

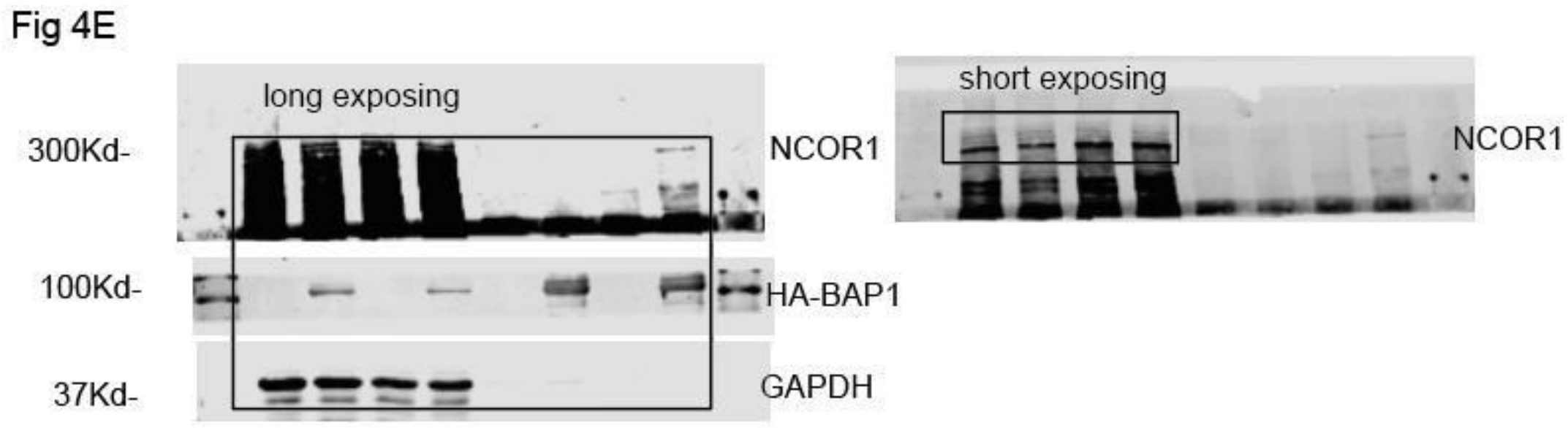
Fig 4E
long exposing
300Kd-
NCOR1
100Kd-
HA-BAP1
37Kd-
GAPDH
short exposing
NCOR1

Figure_4F

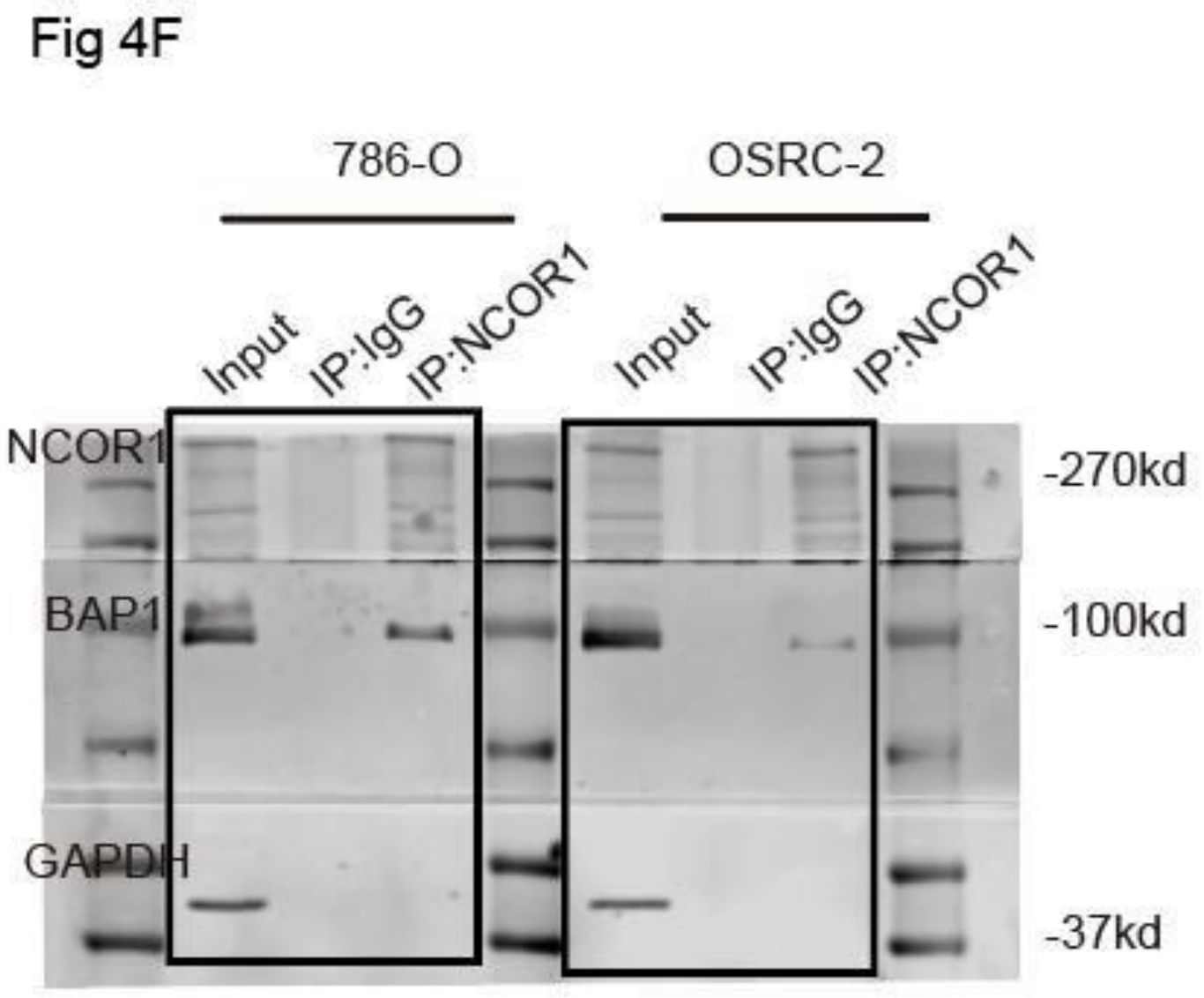
Fig 4F
786-O
OSRC-2
Input
IP:IgG
IP:NCOR1
Input
IP:IgG
IP:NCOR1
NCOR1
BAP1
GAPDH
-270kd
-100kd
-37kd

Figure_4H F4

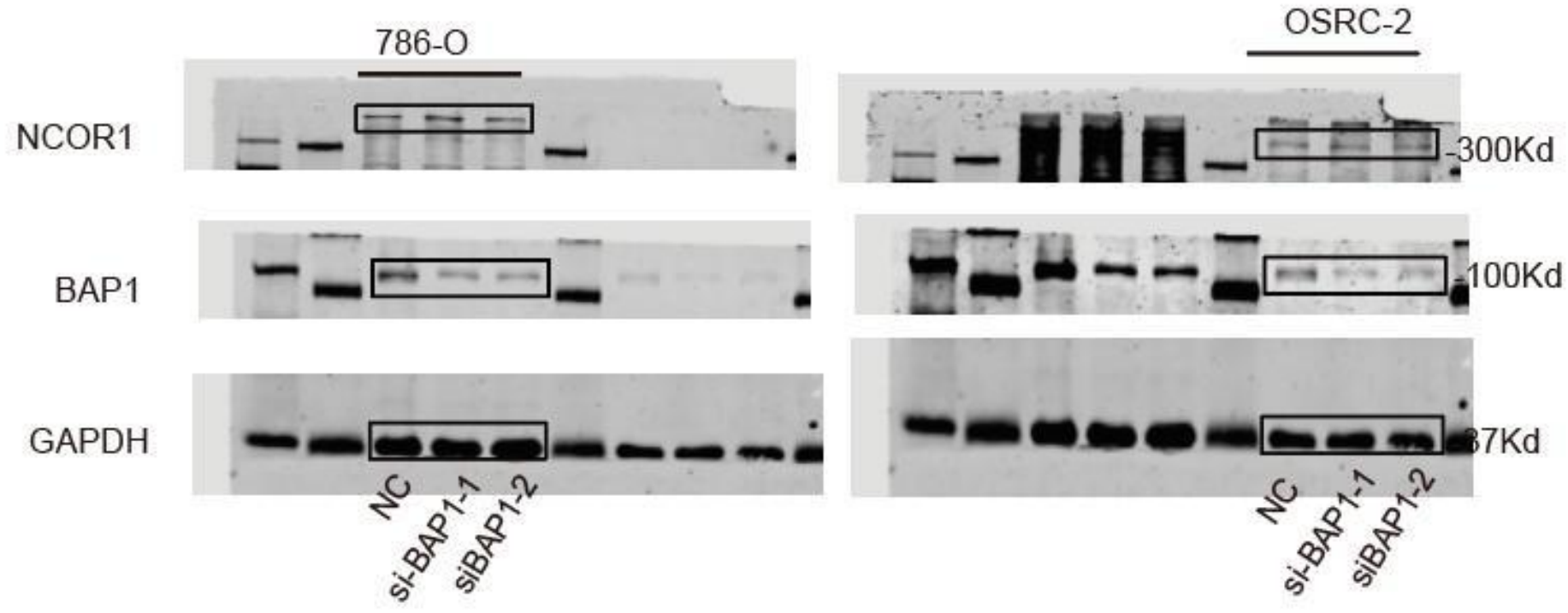

786-O
OSRC-2
NCOR1
BAP1
GAPDH
-300Kd
-100Kd
37Kd
NC
si-BAP1-1
siBAP1-2
NC
si-BAP1-1
siBAP1-2

Figure_4I

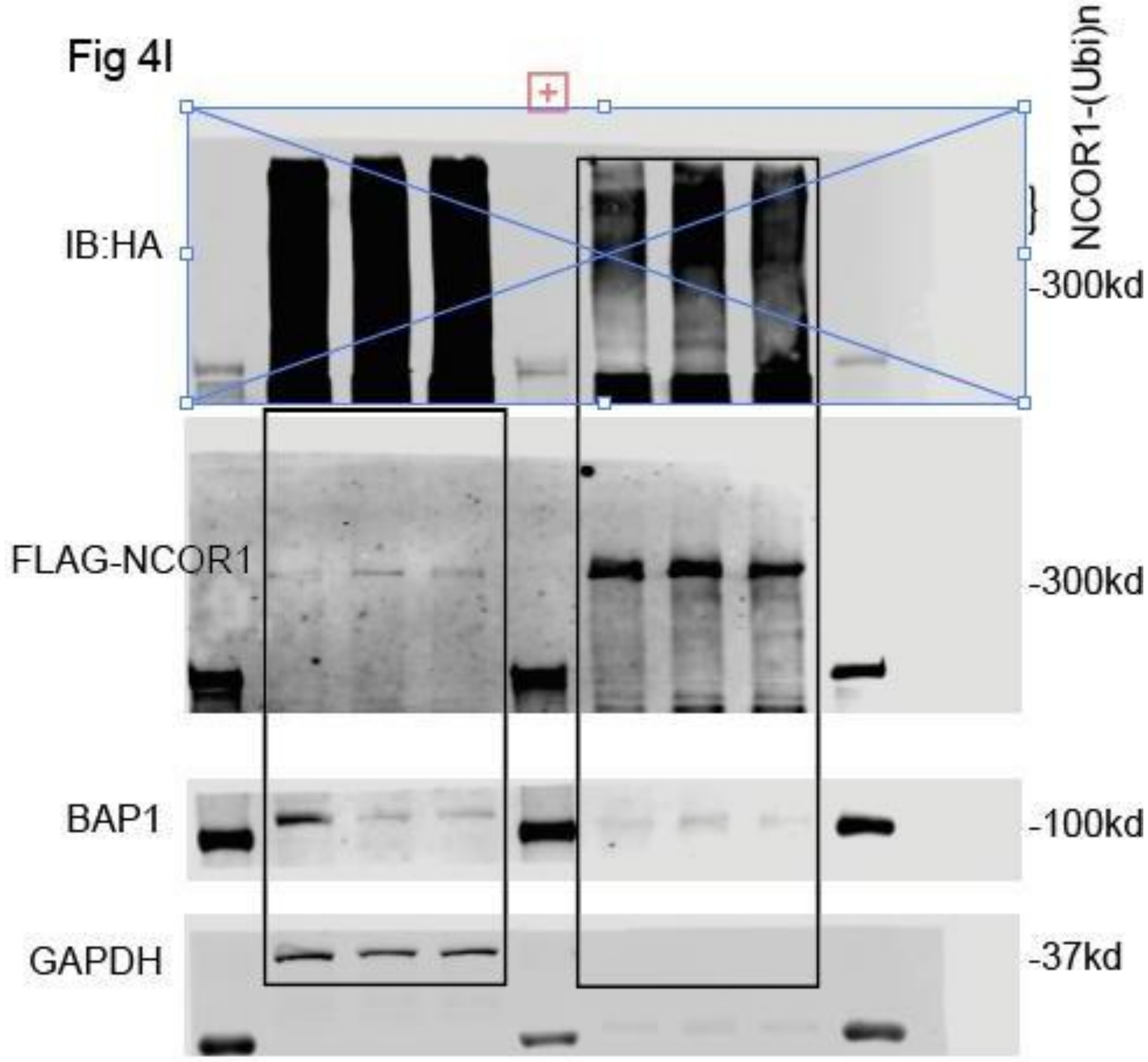

Fig 4I
NCOR1-(Ubi)n
IB:HA
-300kd
FLAG-NCOR1
-300kd
BAP1
-100kd
GAPDH
-37kd

Figure_5B

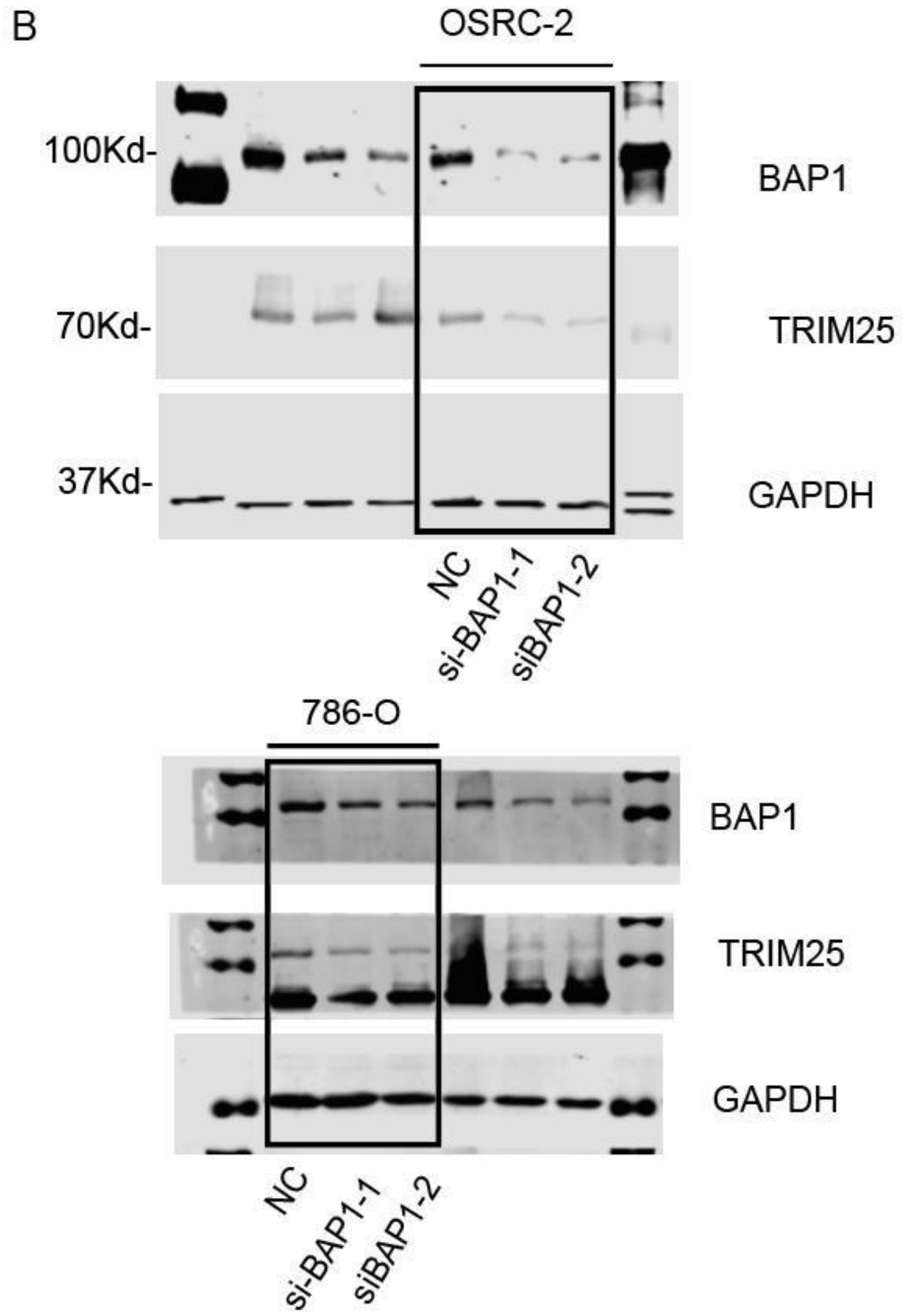
B
OSRC-2
100Kd-
BAP1
70Kd-
TRIM25
37Kd-
GAPDH
NC
si-BAP1-1
siBAP1-2
786-O
BAP1
TRIM25
GAPDH
NC
si-BAP1-1
siBAP1-2

Figure_5C

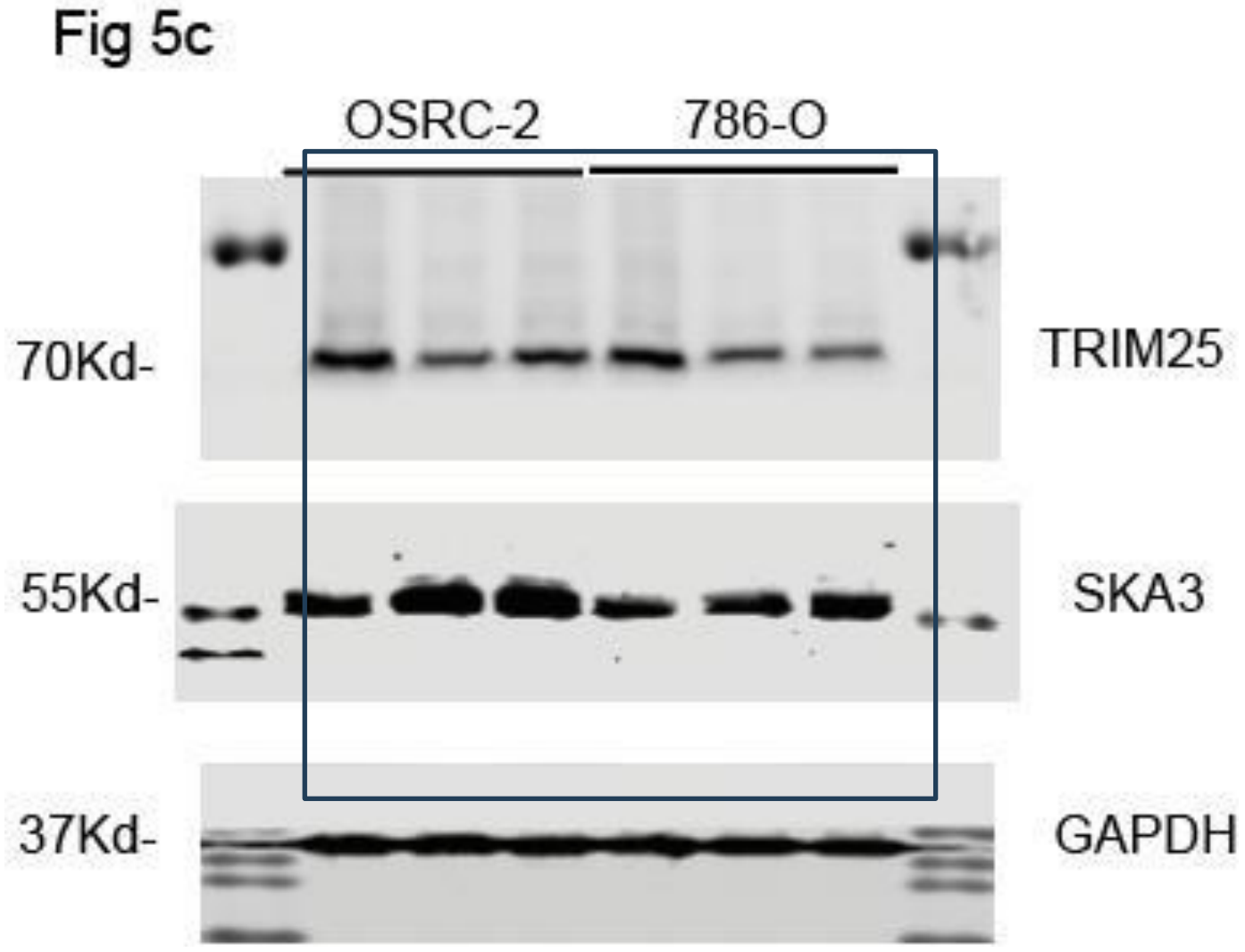

Figure_5D

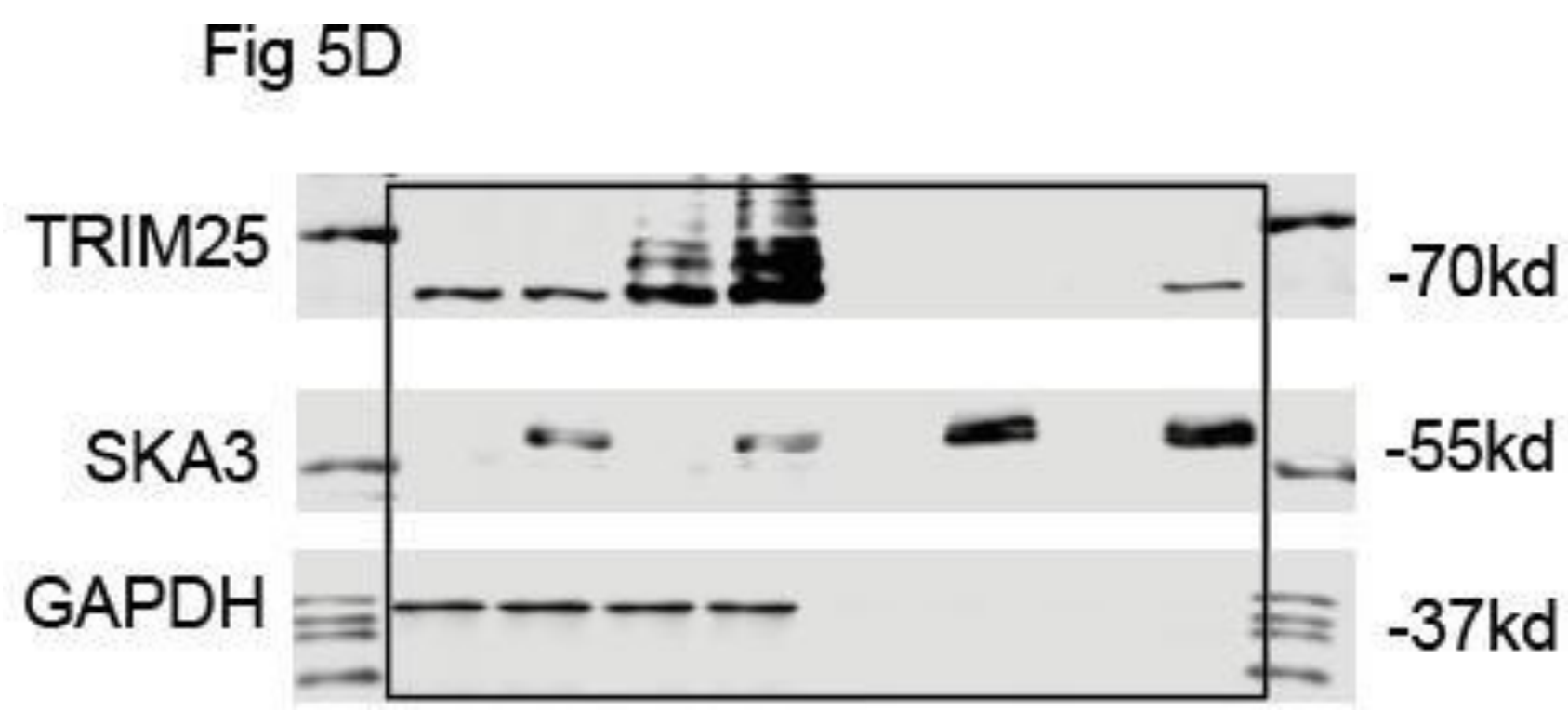

Figure_5E

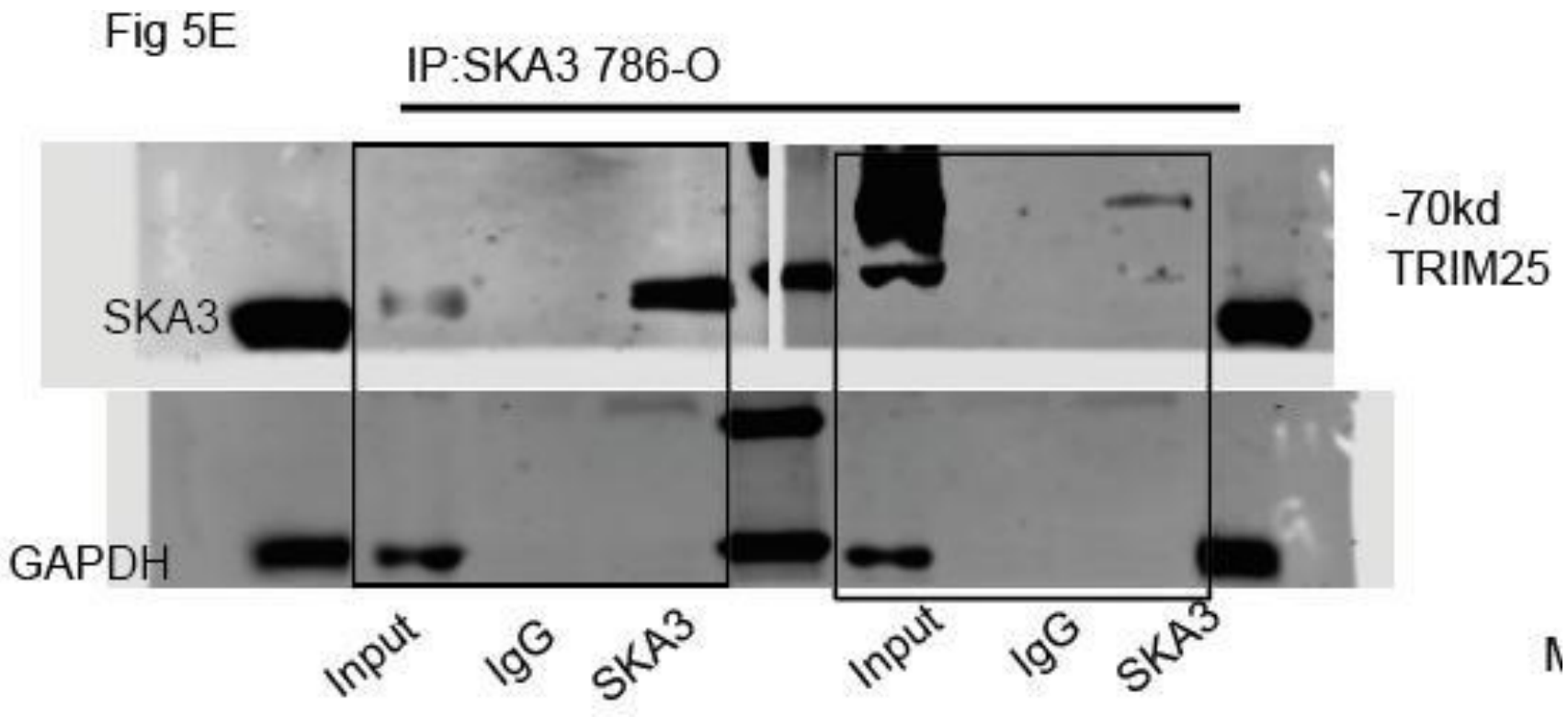
Fig 5E
IP:SKA3 786-O
-70kd
TRIM25
SKA3
GAPDH
Input
IgG
SKA3
Input
IgG
SKA3

Figure_5F

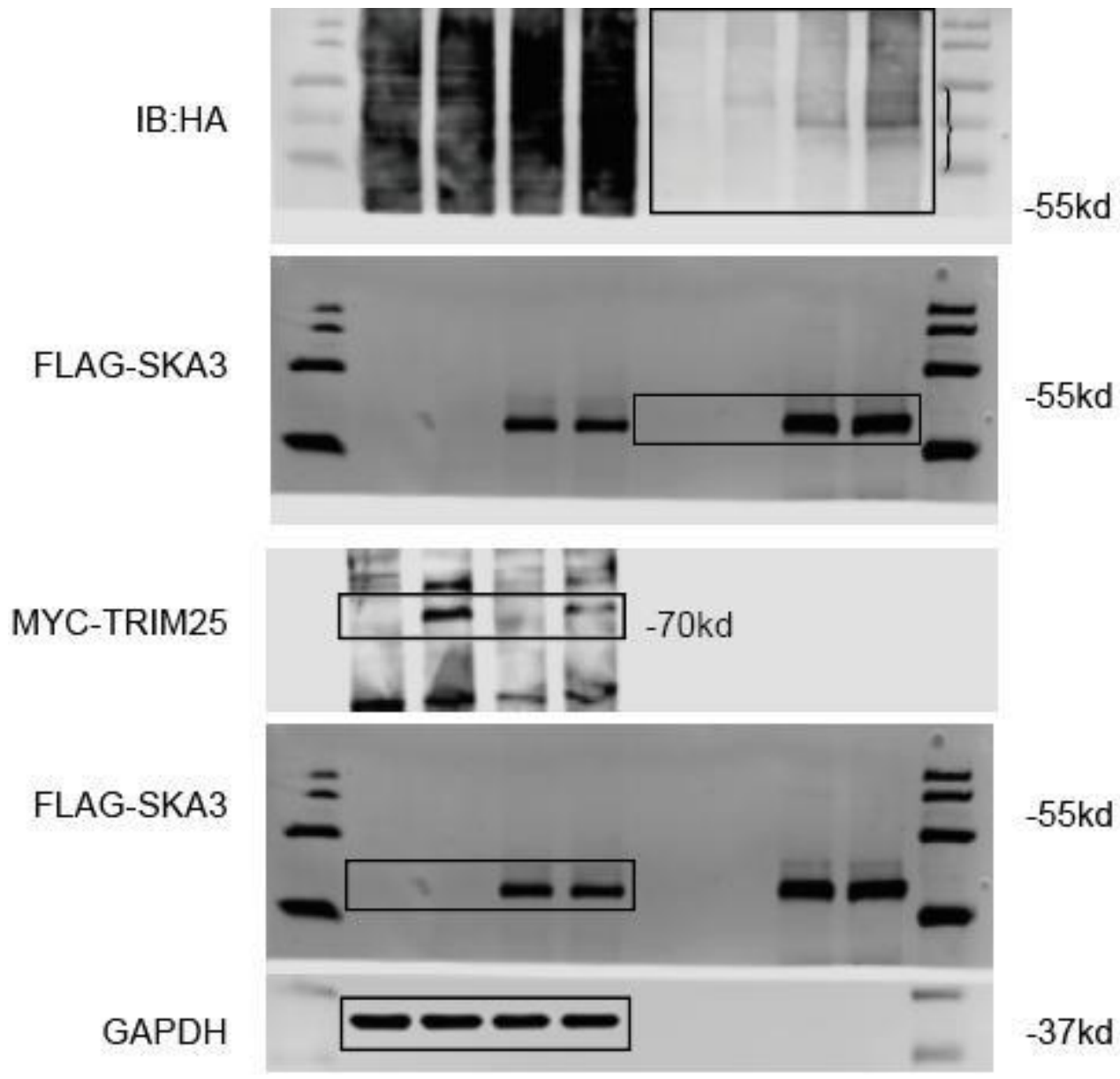
IB:HA
-55kd
FLAG-SKA3
-55kd
MYC-TRIM25
-70kd
FLAG-SKA3
-55kd
GAPDH
-37kd

Figure_5H

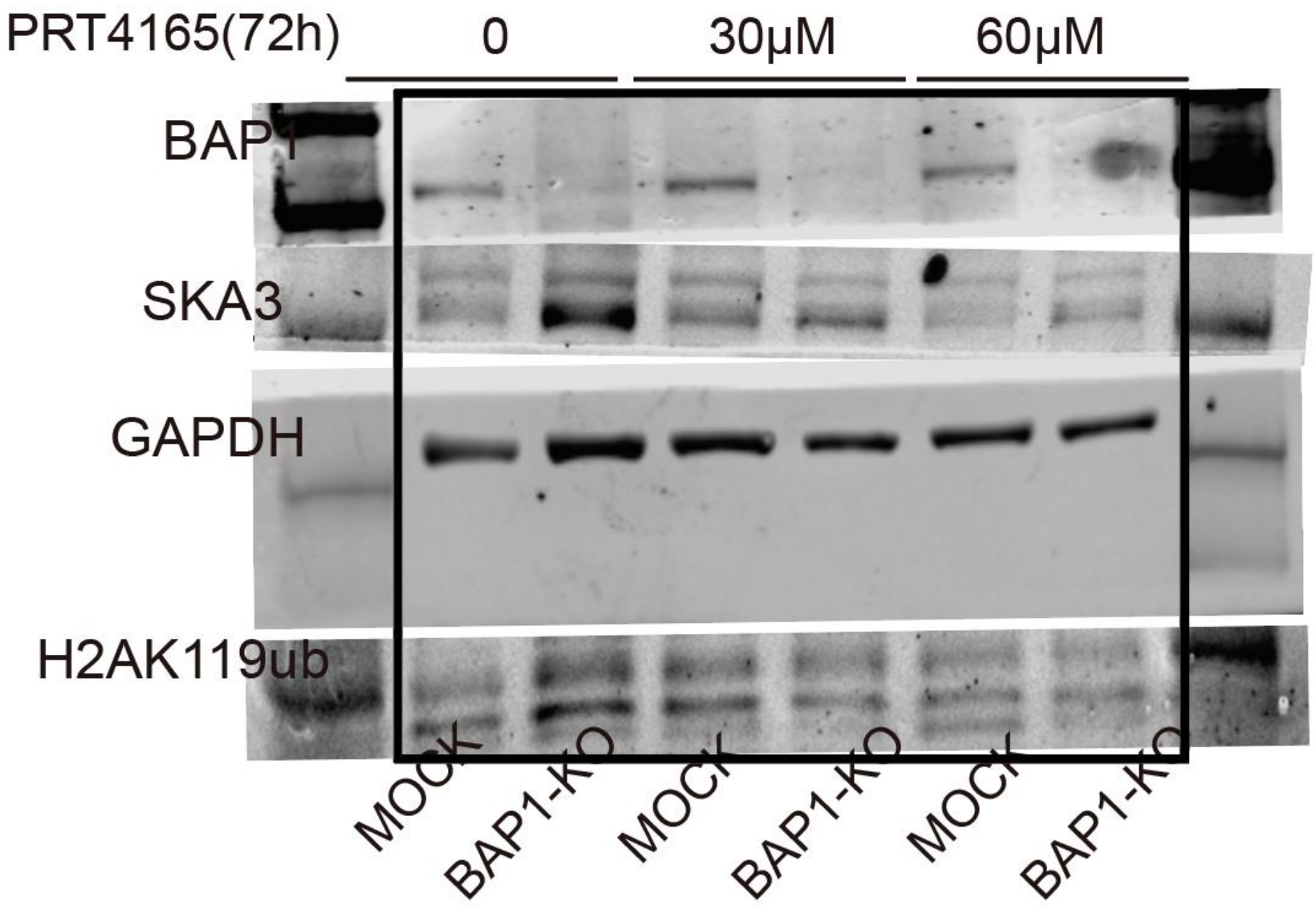

PRT4165(72h)
0
30μM
60μM
BAP1
SKA3
GAPDH
H2AK119ub
MOCK
BAP1-KO
MOCK
BAP1-KO
MOCK
BAP1-KO

Figure_5k

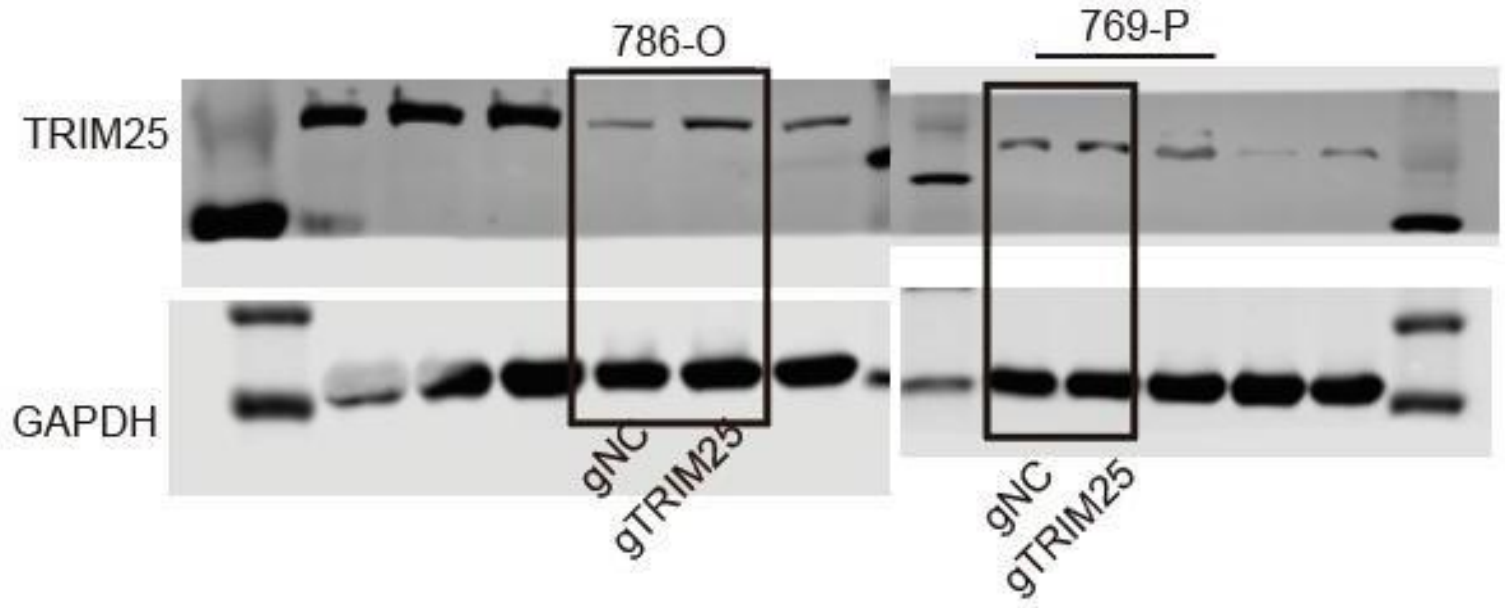

Supplementary_Figure_3B

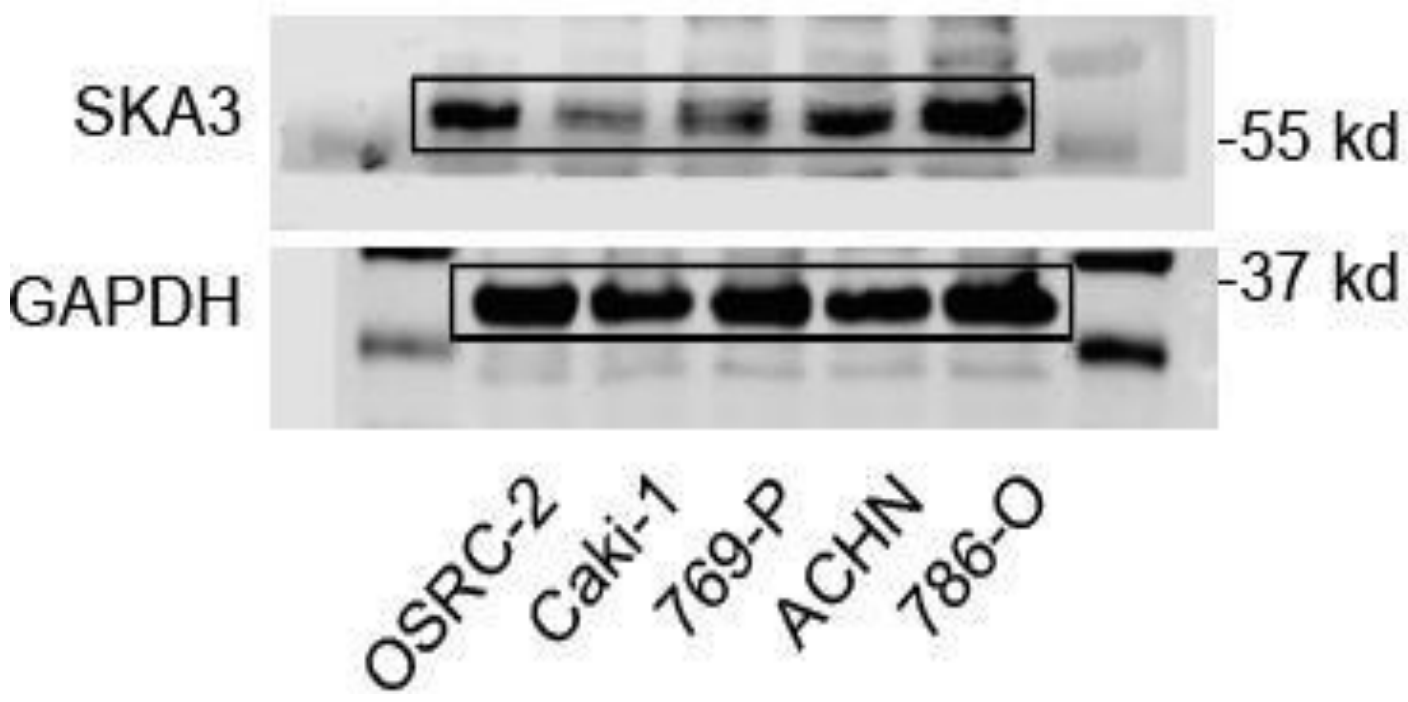

Supplementary_Figure_3C

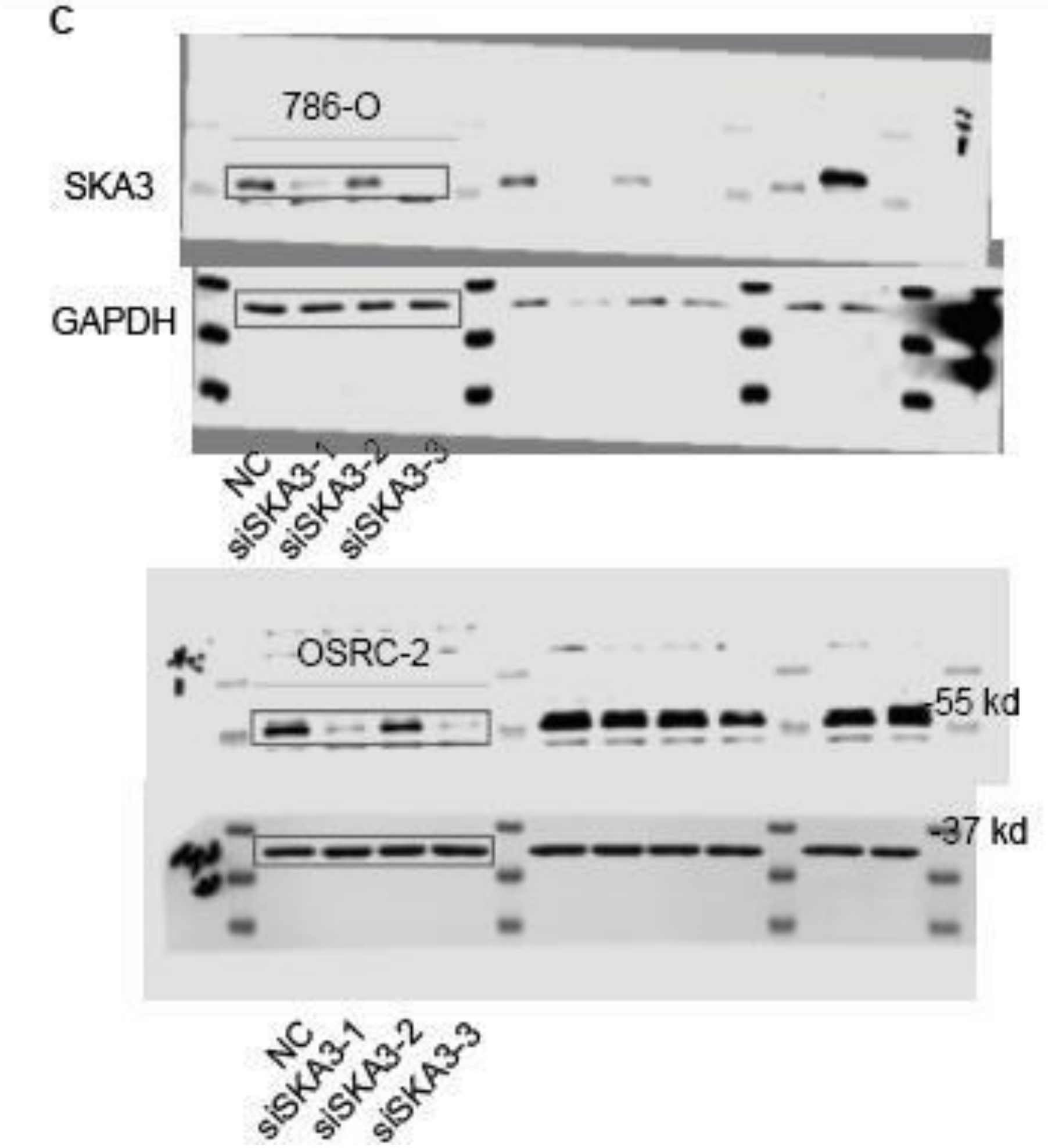

Supplementary_Figure_3D

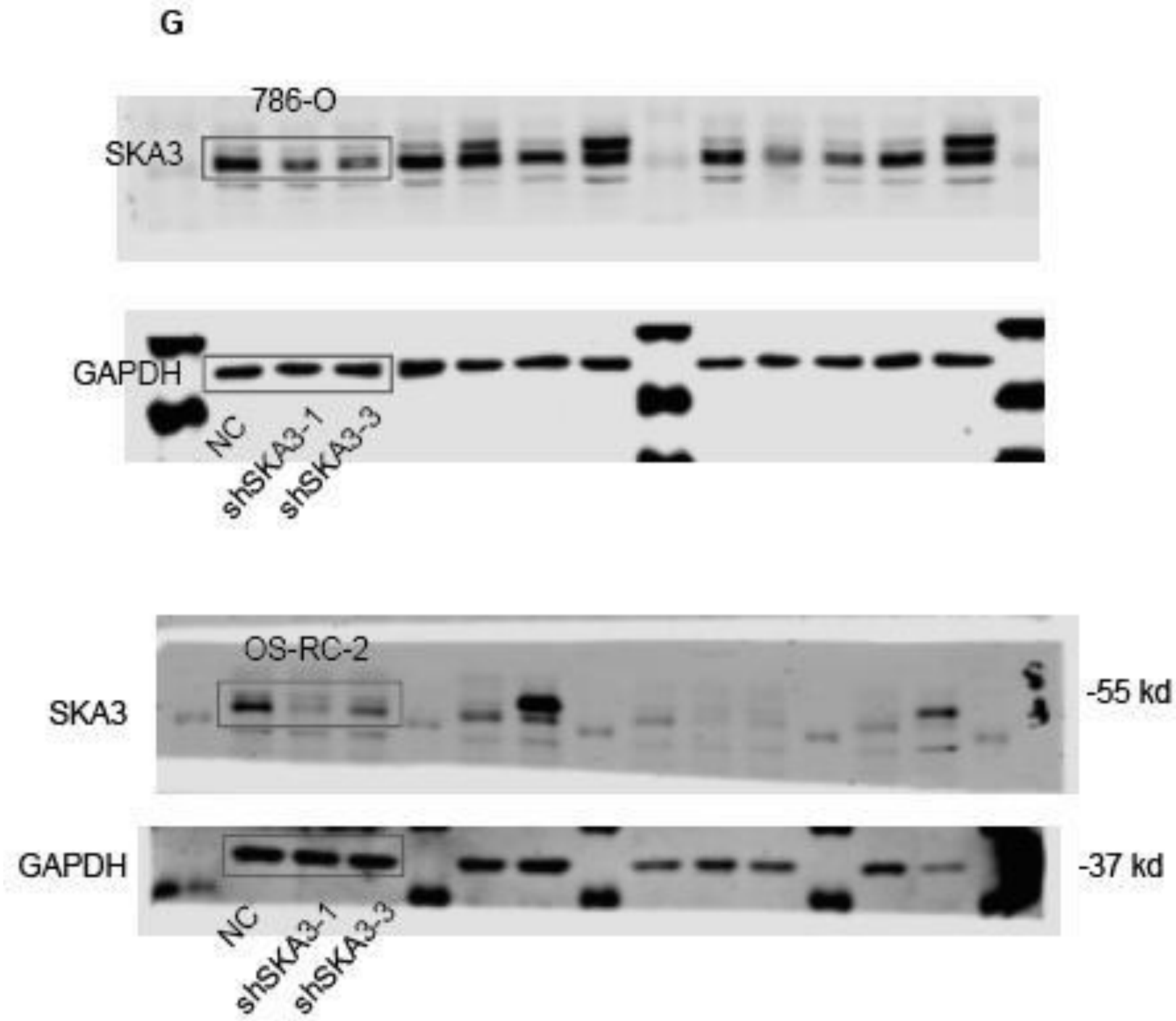

Supplementary_Figure_3G

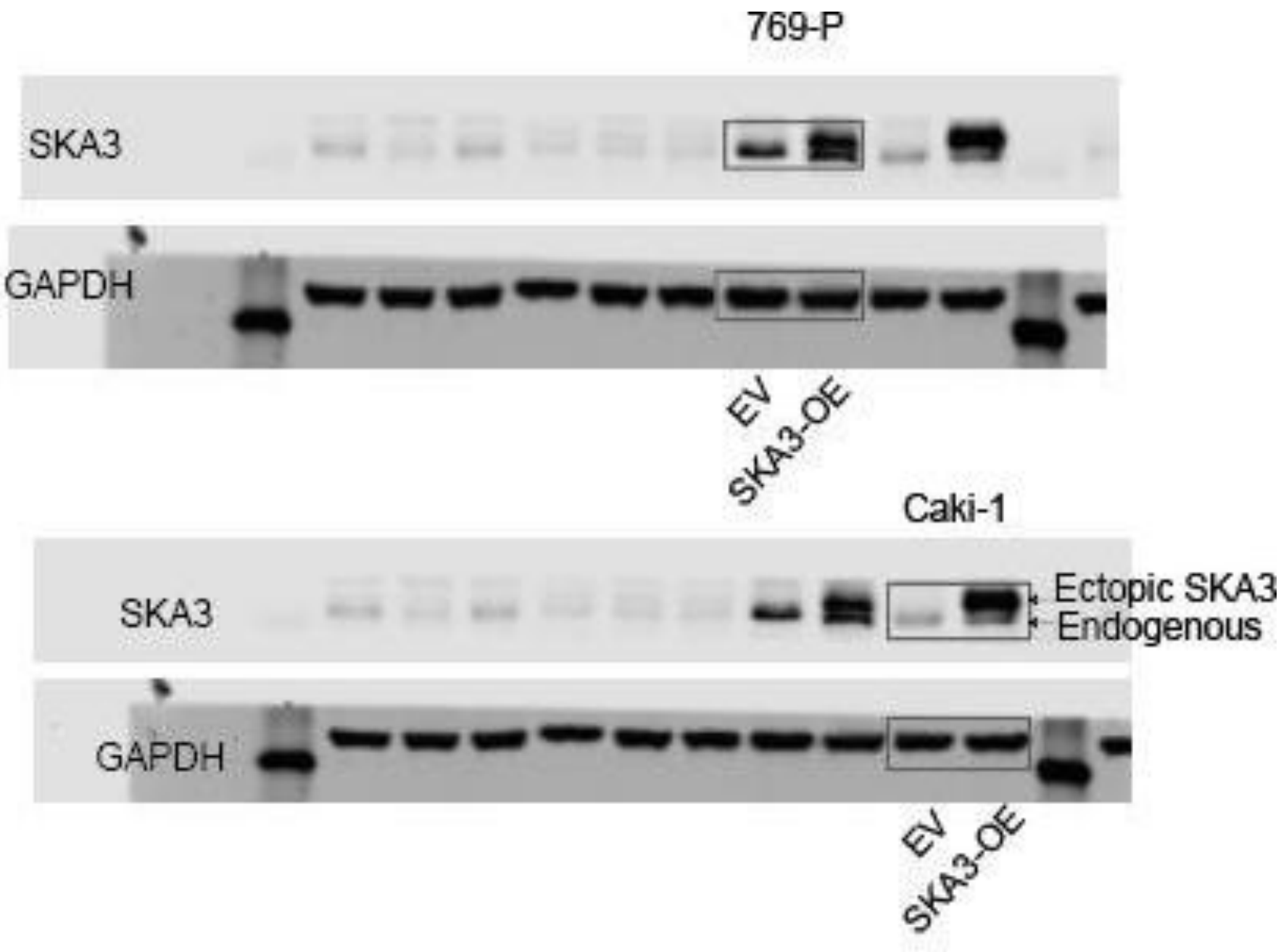

SupplementaryFigure_7C

Fig S7C

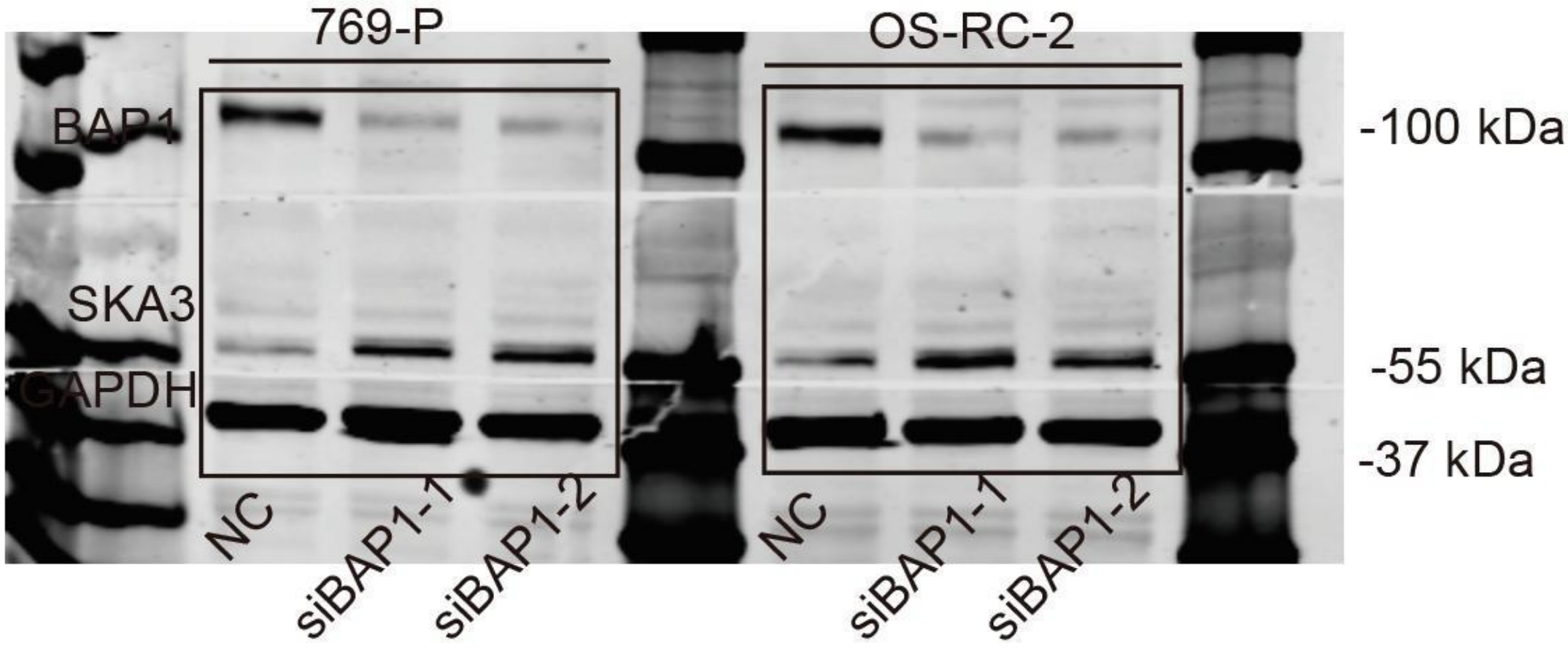

SupplementaryFigure_7E

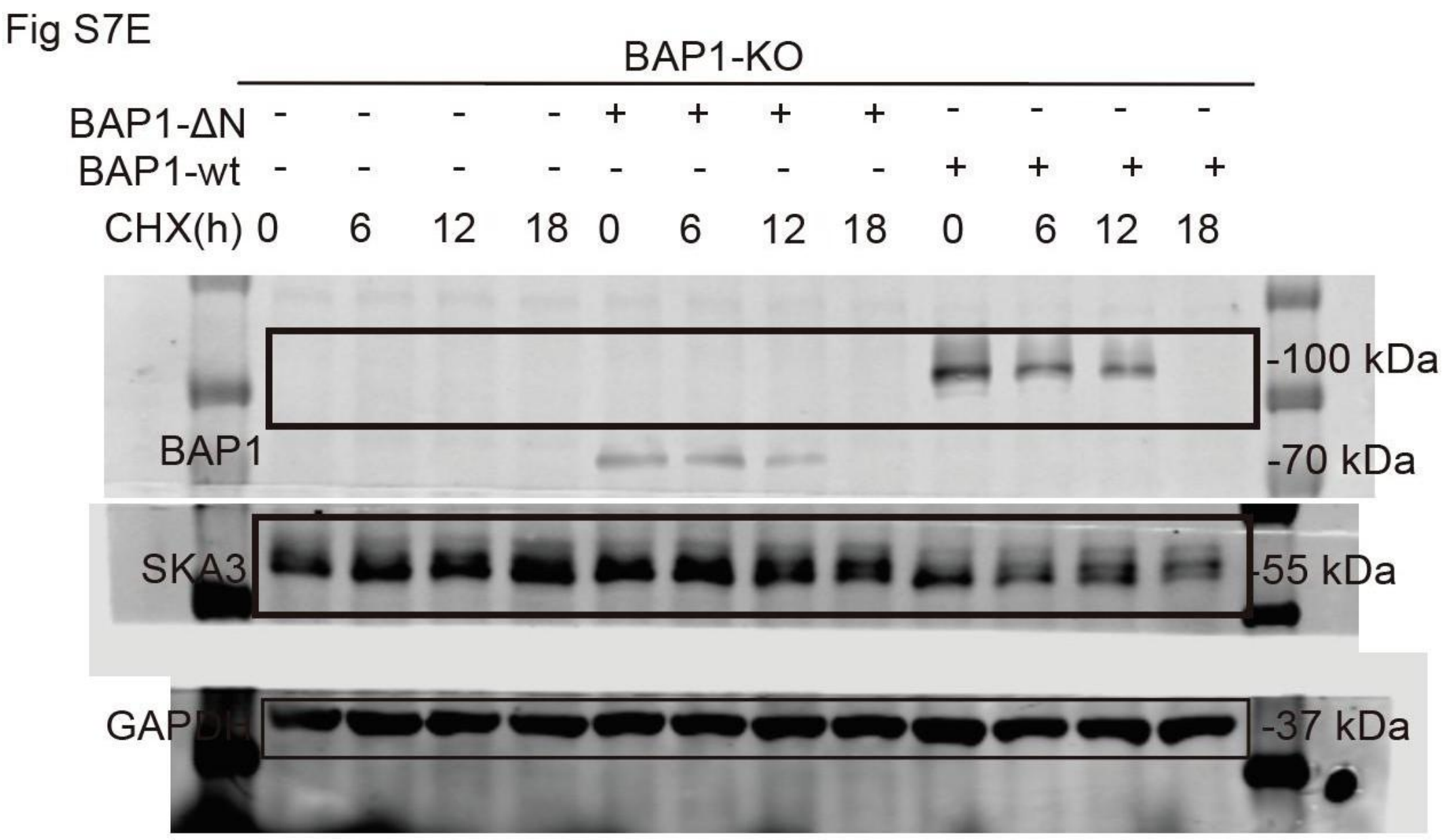

Supplementary Figure_S9A

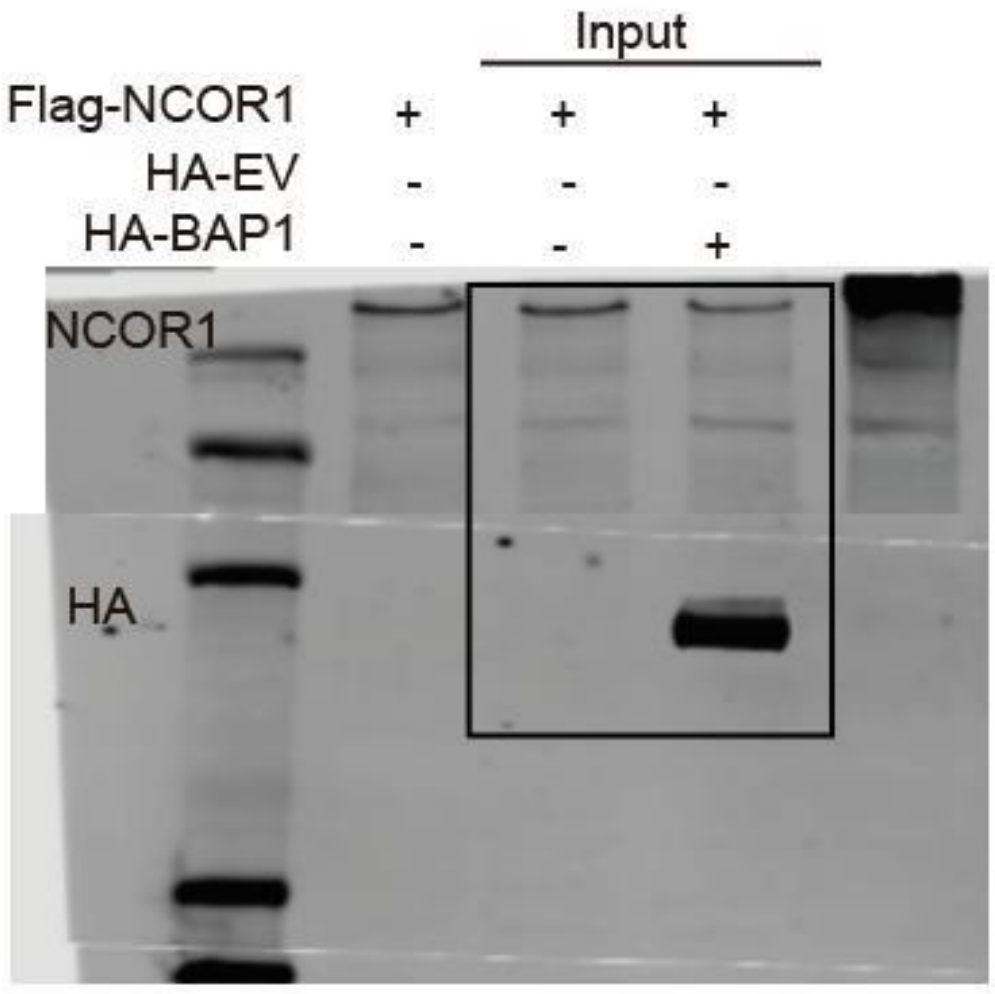

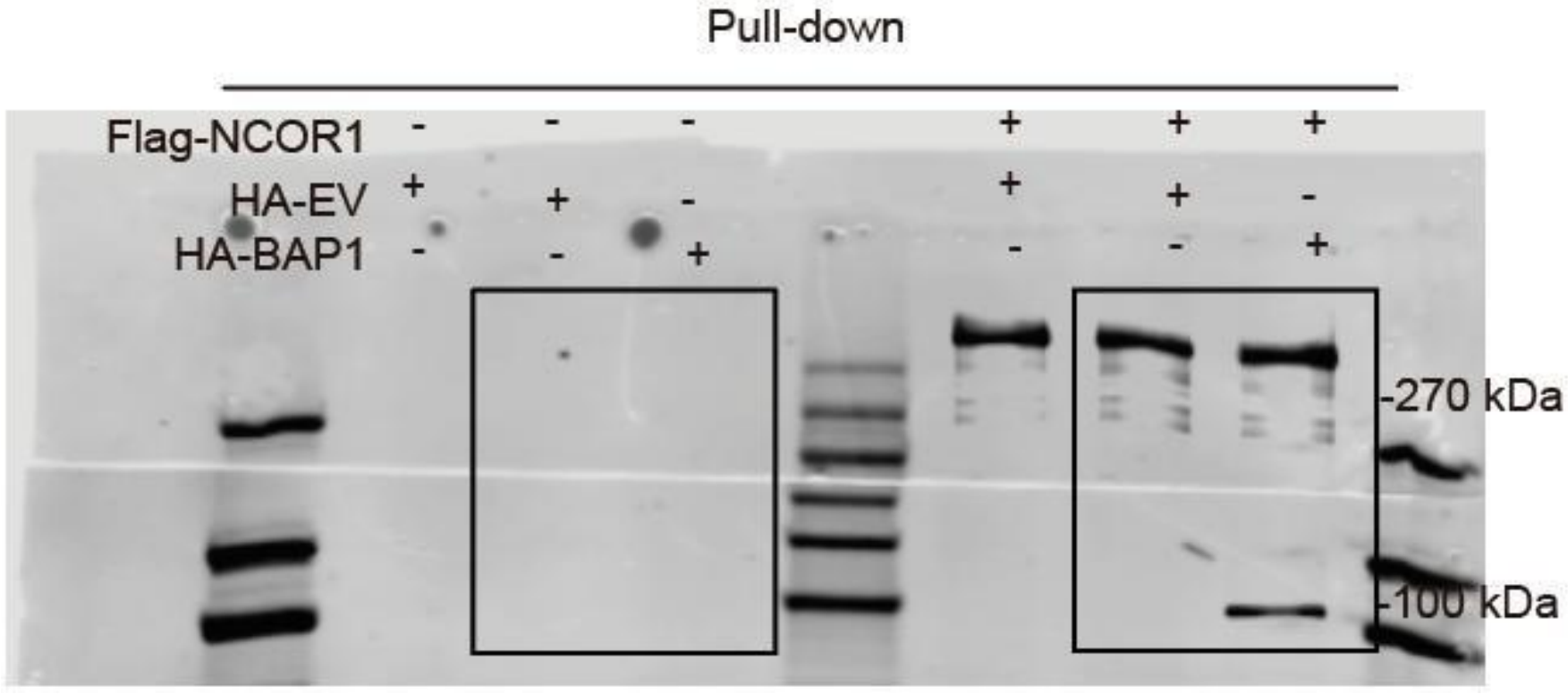

Supplementary Figure_S9C

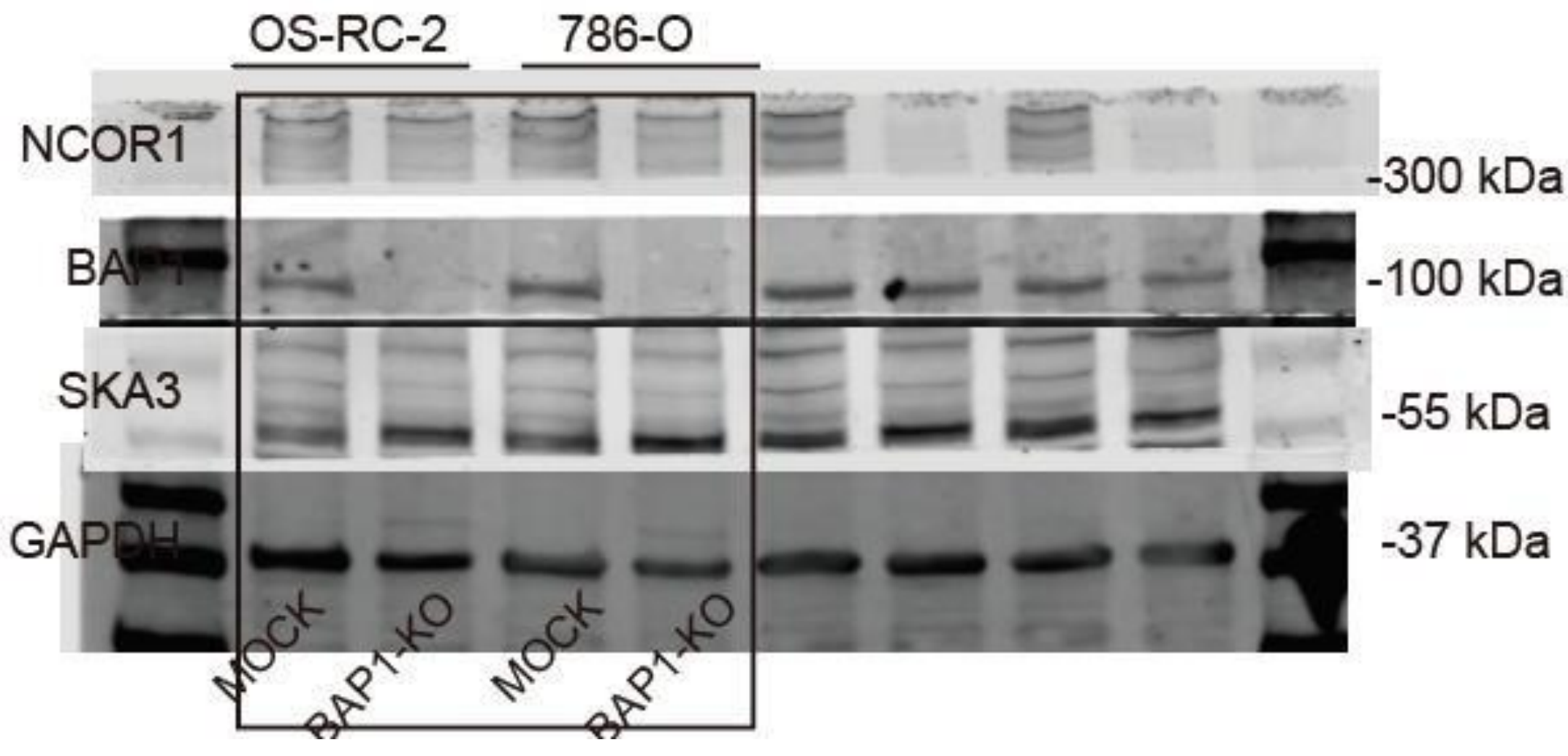

Supplementary Figure_S9D

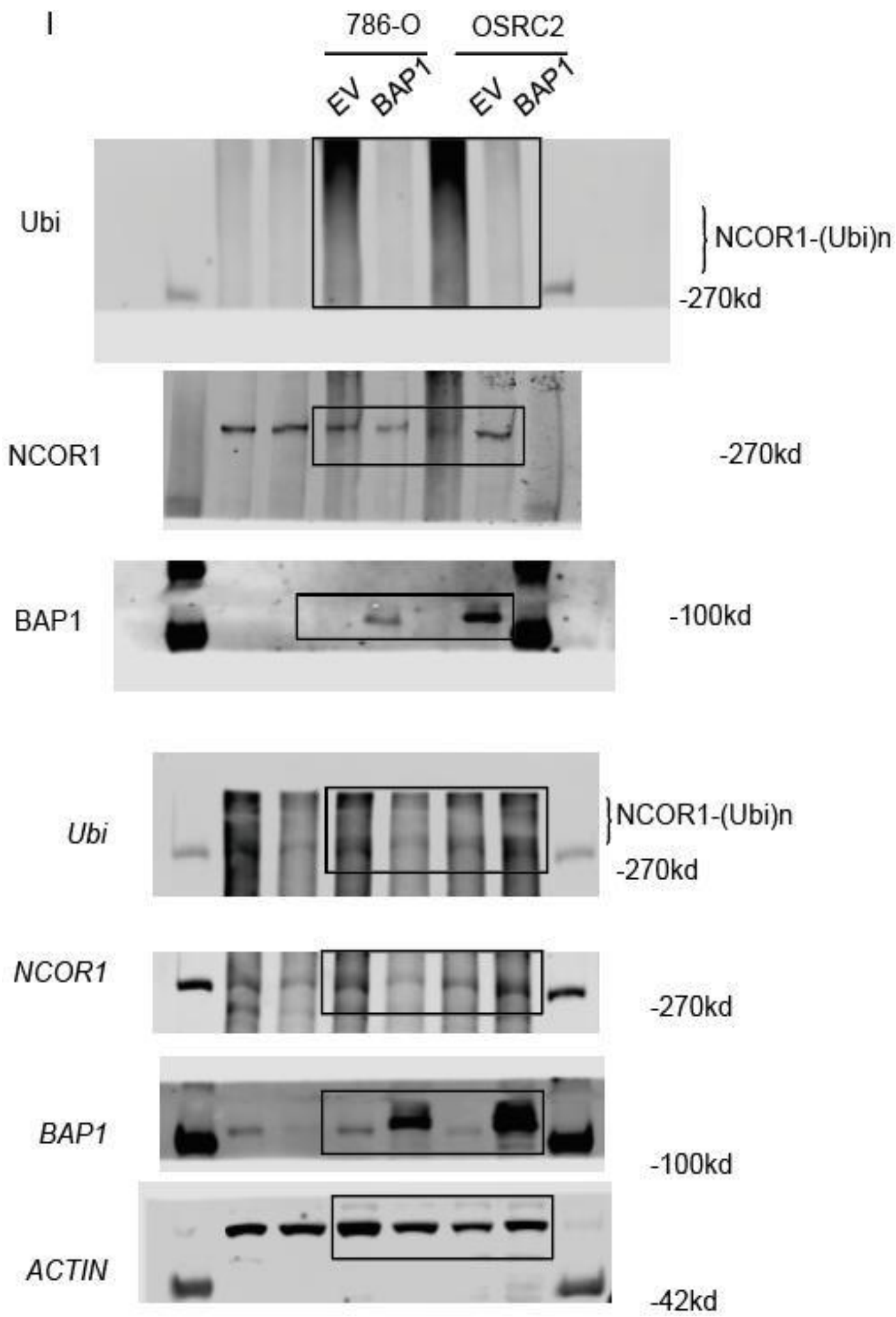

Supplementary Figure_S9E

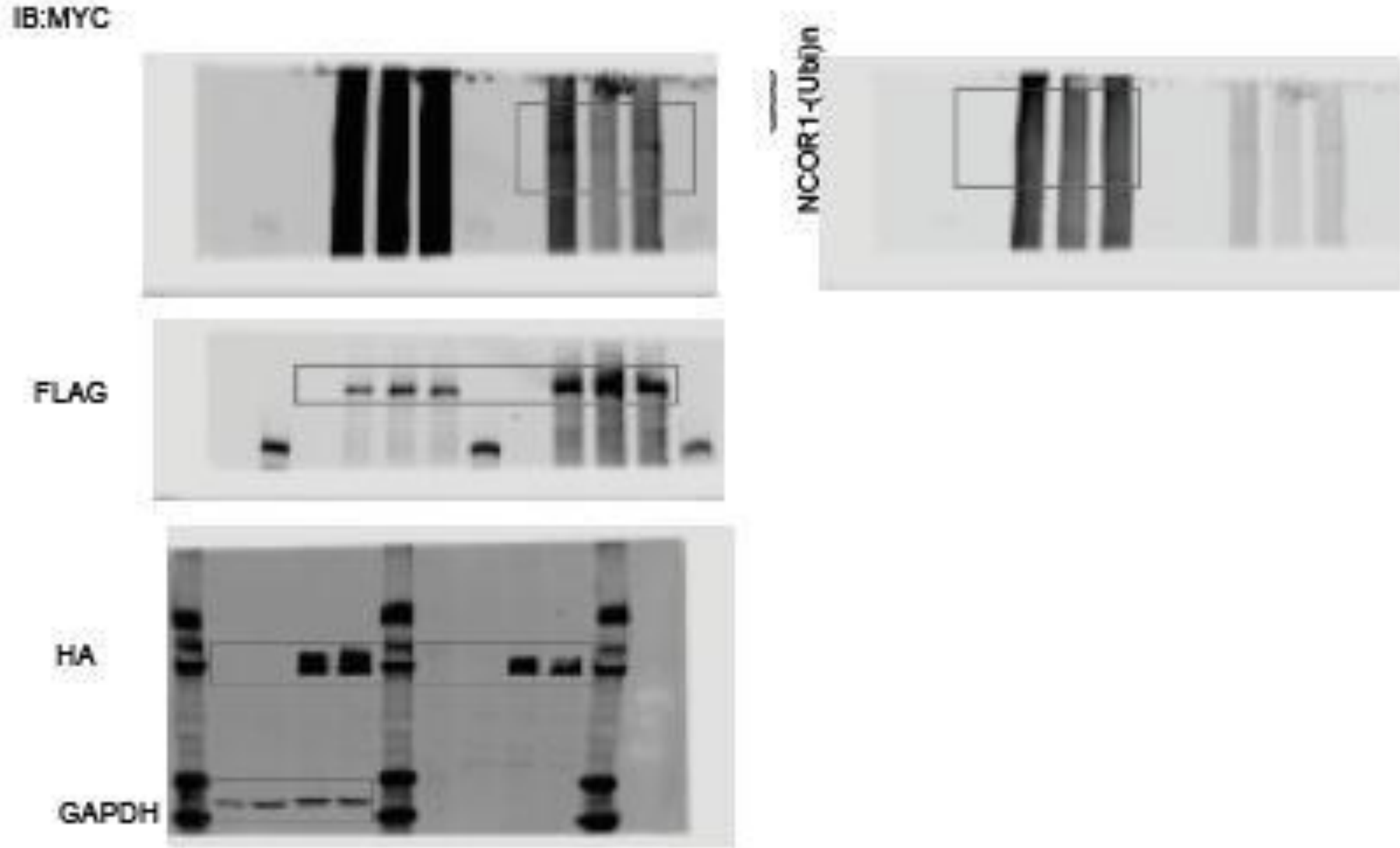

Supplementary Figure_S9G

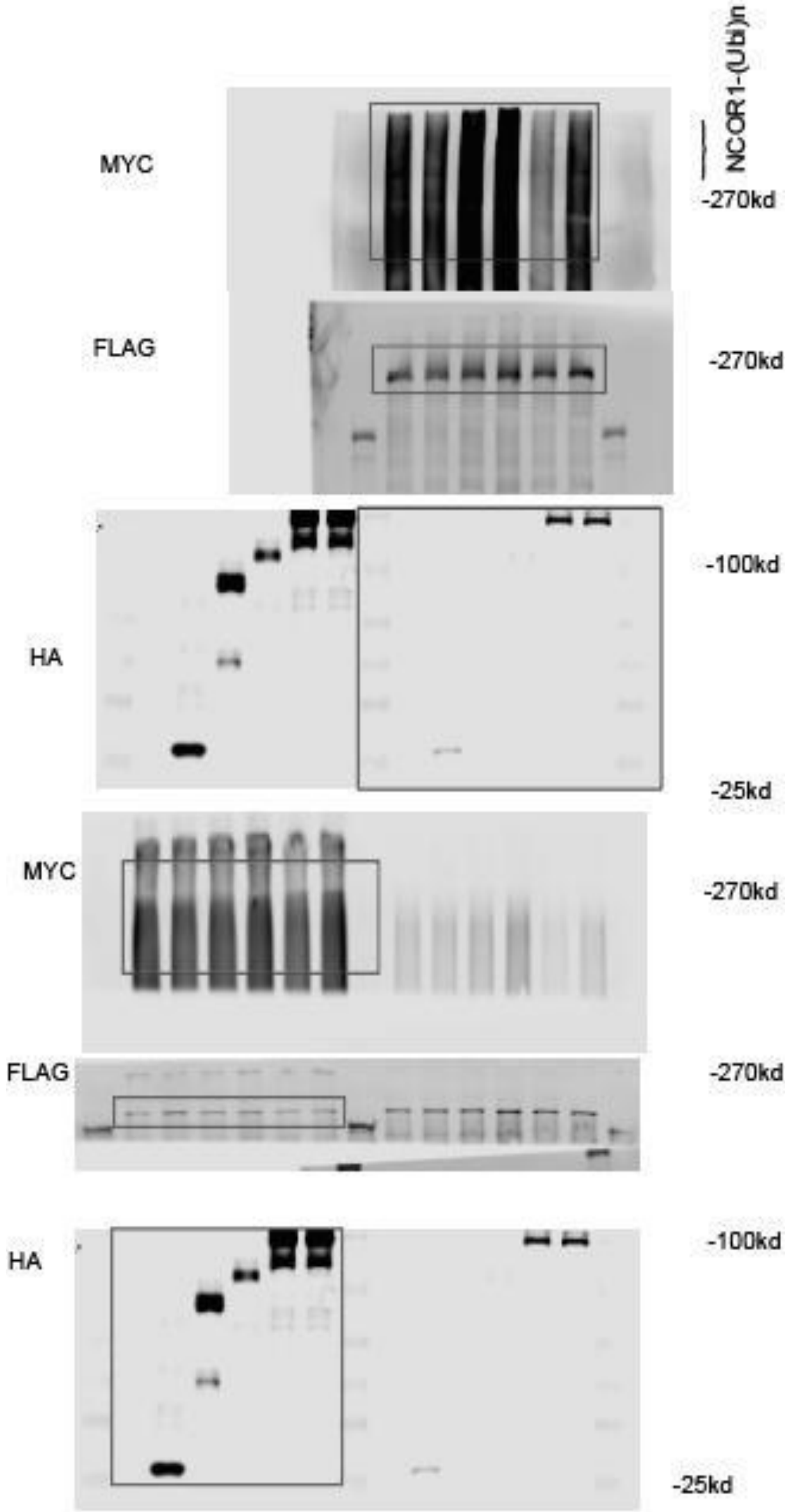

Supplementary Figure_S9I

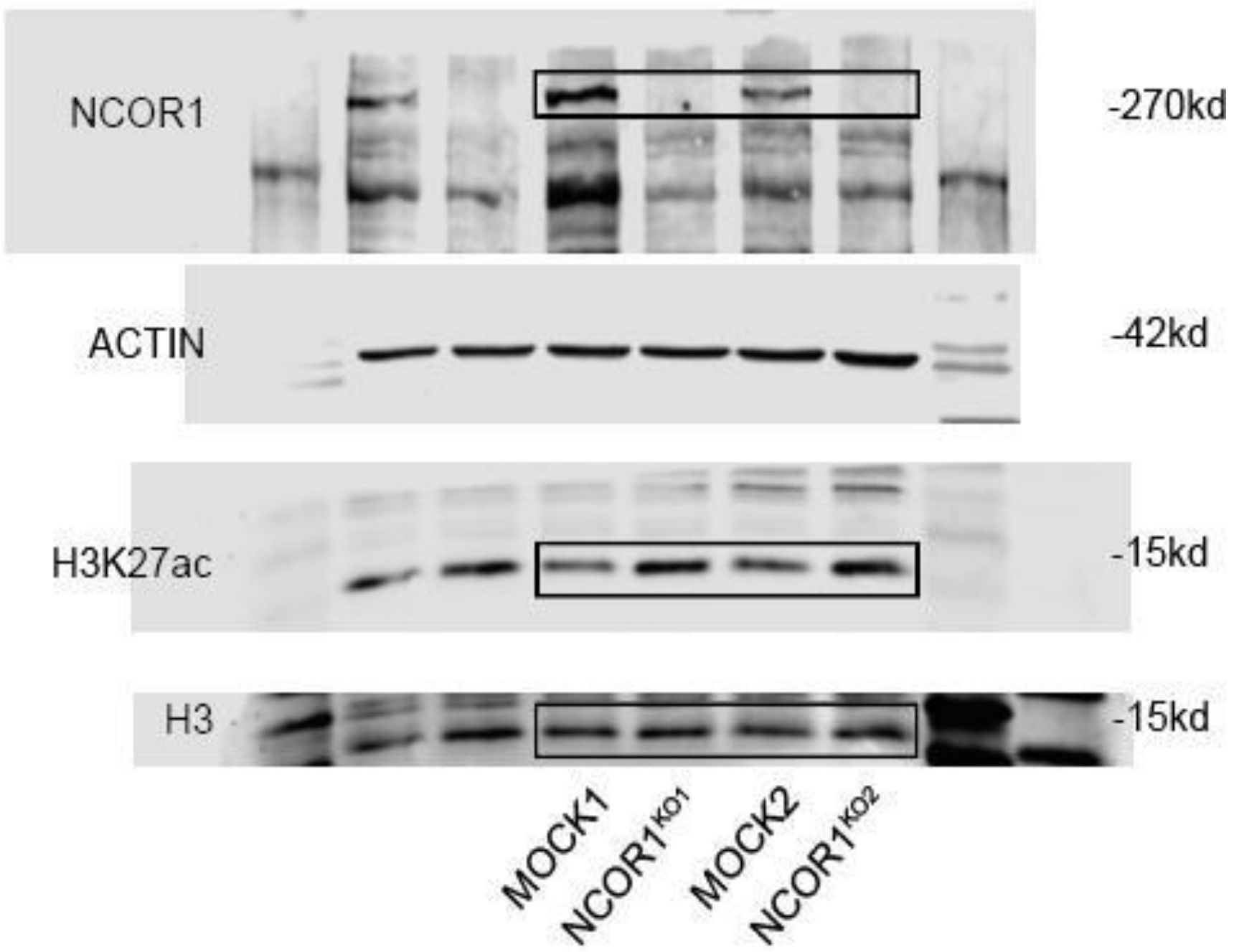

Supplementary Figure_S10C

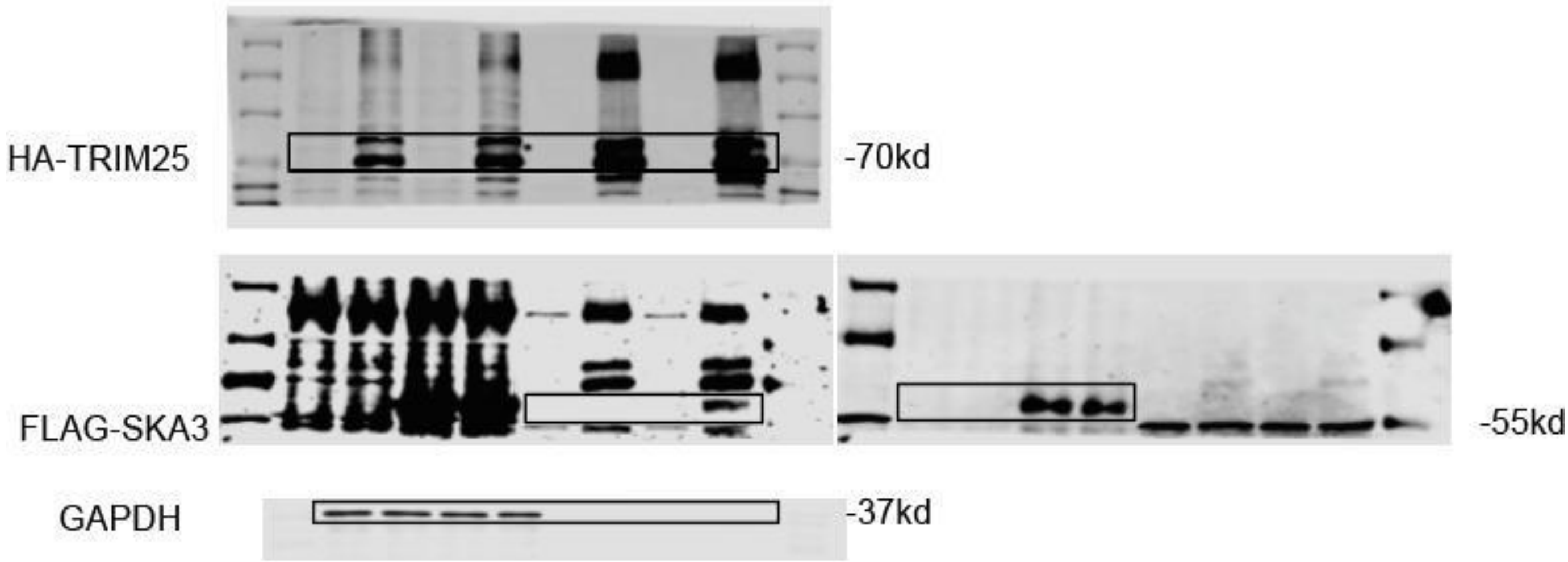

Supplementary Figure_S10D

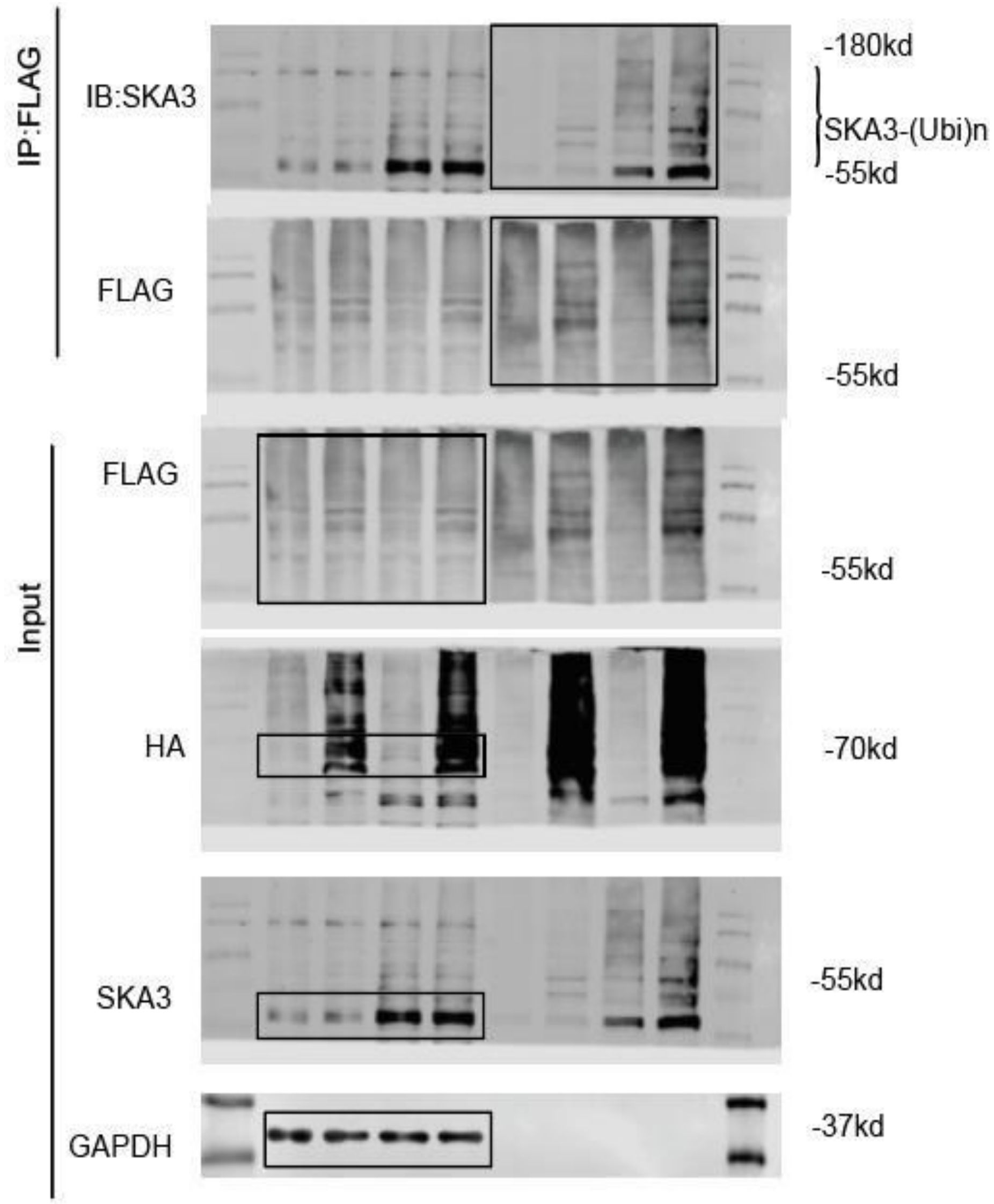

Supplementary Figure_S10H

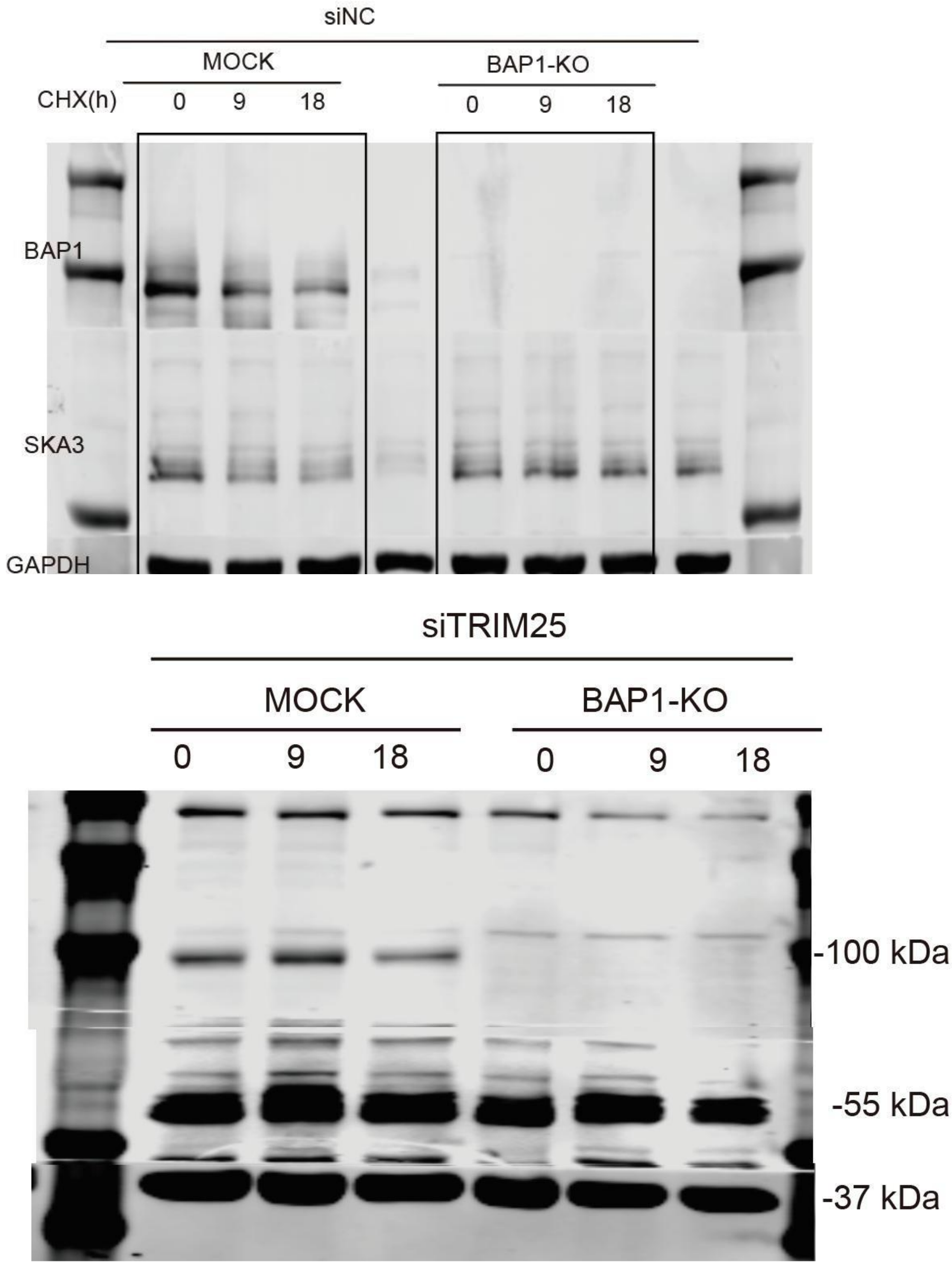

Supplementary Figure_S11A

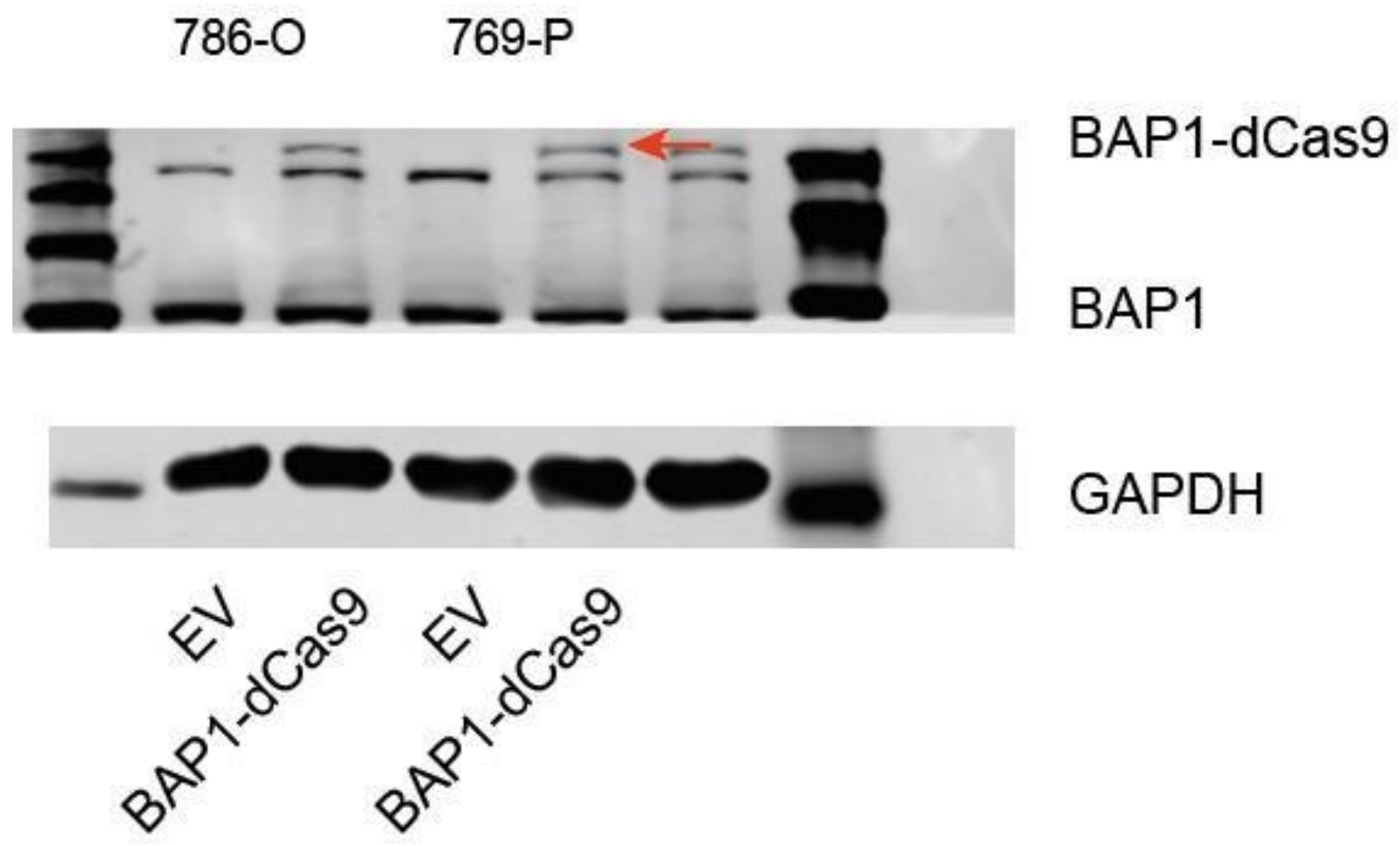

Supplement: Supplementary file 1 [file 41419_2026_9076_MOESM19_ESM.pdf]
