# Supplementary material for: BAP1 deficiency-induced SKA3 promotes clear cell renal cell carcinoma malignant progression by regulating chromosomal instability
Source: Cell Death Dis. 2026 Jul 15;17(1):874. doi: 10.1038/s41419-026-09076-3 (PMC13649664; doi:10.1038/s41419-026-09076-3)
Supplement: Supplementary file 2 [file 41419_2026_9076_MOESM14_ESM.docx]

**Supporting Information for**

**BAP1 Deficiency-Induced SKA3 Promotes Clear Cell Renal Cell Carcinoma Malignant Progression by Regulating Chromosomal Instability**

Yueyang Wang^1,2,3^, Chong Lai^4^, Wang Lingling^1,2,3^, Jingwen Deng^5^, Zhou Tian^6^, Zhiyong Liang^1*^, Honghe Zhang^2,3,6 *^

1 Department of Pathology, Peking Union Medical College Hospital, Chinese Academy of Medical Science & Peking Union Medical College, Beijing 100730, China

2 Research Unit of Intelligence Classification of Tumor Pathology and Precision Therapy, Chinese Academy of Medical Sciences (2019RU042) and Zhejiang University School of Medicine, Hangzhou, 310058, China.

3 Department of Pathology, Zhejiang University School of Medicine, Hangzhou, 310058, China.

4 Department of Urology, the First Affiliated Hospital, Zhejiang University School of Medicine, Hangzhou, 310003, China

5 Department of Medical Oncology, Sir Run Run Shaw Hospital, School of Medicine, Zhejiang University, Hangzhou, 310016, China.

6 Department of Surgery, The Fourth Affiliated Hospital, International Institutes of Medicine, Zhejiang University School of Medicine, Yiwu, 322000, China

*Correspondence to: Honghe Zhang ([honghezhang@zju.edu.cn](mailto:honghezhang@zju.edu.cn)) and Zhiyong Liang ([liangzy@pumch.cn](mailto:liangzy@pumch.cn))

**This PDF file includes:**

Figures. S1 to S8

Tables S1 to S3

Captions for Movies S1 to S3

**Other Supplementary Materials for this manuscript include the following:**

Movie S1 – S3

Data S1 [Source data]

TABLE OF CONTENTS

[Supplementary Materials and Methods	1](#_Toc230812157)

[Cell culture	1](#_Toc230812158)

[Clinical materials	1](#_Toc230812159)

[siRNA synthesis, plasmid construction and transfection	2](#_Toc230812160)

[Generation of knockout cells	3](#_Toc230812161)

[RNA extraction, reverse transcription and real-time quantitative PCR (RT-qPCR)	3](#_Toc230812162)

[Western blotting	4](#_Toc230812163)

[Immunofluorescence assay	5](#_Toc230812164)

[Immunoprecipitation assay	6](#_Toc230812165)

[Cell cycle analysis	7](#_Toc230812166)

[Hematoxylin and Eosin Staining (H&E Staining) AND Immunohistochemistry (IHC)	8](#_Toc230812167)

[Chemical treatment	8](#_Toc230812168)

[Public Database Analysis	9](#_Toc230812169)

[Supplementary Figure and Figure legends	10](#_Toc230812170)

[Supplementary Figure S1. Computational screening of mitosis-related genes in ccRCC.	10](#_Toc230812171)

[Supplementary Figure S2. Clinical and prognostic significance of SKA3.	11](#_Toc230812172)

[Supplementary Figure S3. SKA3 promote ccRCC cell proliferation and cell cycle progression.	11](#_Toc230812173)

[Supplementary Figure S5. SKA3 fails to modulate cGAS-STING-mediated ISG15/IRF3 levels.	14](#_Toc230812174)

[Supplementary Figure S6. BAP1 loss induces SKA3 overexpression in ccRCC.	15](#_Toc230812175)

[Supplementary Figure S7. BAP1 deficiency promotes proliferation and CIN of ccRCC via SKA3.	16](#_Toc230812176)

[Supplementary Figure S8. BAP1 fails to modulate H2AK119ub enrichment at the *SKA3* promoter.	17](#_Toc230812177)

[Supplementary Figure S9. BAP1 facilitates NCOR1 deubiquitination through DUB activity.	18](#_Toc230812178)

[Supplementary Figure S10. BAP1 promotes TRIM25 expression to drive SKA3 degradation.	19](#_Toc230812179)

[Supplementary Figure S12. BAP1 promotes genomic damage in ccRCC cells.	21](#_Toc230812180)

[Supplementary Figure S13. BAP1 and SKA3 regulate genomic damage in ccRCC	22](#_Toc230812181)

[Table S1 (separate file)	23](#_Toc230812182)

[Table S2: The oligonucleotide Squence of siRNA/sgRNA.	23](#_Toc230812183)

[Table S3: The oligonucleotide Squence of qPCR primers	24](#_Toc230812184)

[Table S4. Multivariable Cox Regression Models for Assessing the Proliferation-Independent Prognostic Value of SKA3 in ccRCC	25](#_Toc230812185)

[Movie S1 - S3. (separate file)	26](#_Toc230812186)

[Data S1. (separate file)	26](#_Toc230812187)

Supplementary Materials and Methods

**Cell culture**

Human RCC cell lines 769-P (RRID: CVCL_1050), 786-O (RRID: CVCL_1051), Caki-1 (RRID: CVCL_0234) and ACHN (RRID: CVCL_1067) were obtained from the American Type Culture Collection (ATCC, USA). Human embryonic kidney 293T cells (HEK293T) (RRID: CVCL_0063) and the RCC cell line OS-RC-2 (RRID: CVCL_1626) were purchased from the National Collection of Authenticated Cell Cultures (Cell Bank/Stem Cell Bank, China). HEK293T and ACHN cells were cultured in Dulbecco’s modified eagle medium (DMEM) (Gibco, USA). 769-P, 786-O and OS-RC-2 were cells cultured in Roswell Park Memorial Institute (RPMI) 1640 medium (Gibco). Caki-1 cells were cultured in McCoy’s 5A medium (Gibco). All culture media were supplemented with 10% fetal bovine serum (ExCell Bio, FSP025, China), 100 U/ml penicillin, and 100 μg/ml streptomycin (Beyotime Biotechnology, C0222, China). All cell lines were cultured at 37 ℃ in a humidified incubator with 5% CO_2_. All cell lines were authenticated using short tandem repeat (STR) profiling analysis. All experiments were performed using cell lines with a low passage number (≤15). All cell lines were routinely tested for mycoplasma contamination using MycAway™ Plus-Color One-Step Mycoplasma Detection Kit (Yeasen, China) and tested negative for mycoplasma contamination.

**Clinical materials**

Human samples were obtained from patients who underwent radical or partial Nephrectomy at the First Affiliated Hospital, Zhejiang University School of Medicine. All specimens were collected after resection and fixed in 10% neutral buffered formalin within 30 minutes for 24 - 48 h at room temperature. Finally, tissues were embedded in paraffin blocks for subsequent histological sectioning and examination. This study was approved by the Ethics Committee of Zhejiang University, and all patients have signed the informed consent form. This study was approved by the Institutional Ethics Committee of the First Affiliated Hospital, Zhejiang University School of Medicine (2021IIT923).

**siRNA synthesis, plasmid construction and transfection**

siRNAs targeting SKA3, BAP1, NCOR1, TRIM25, and SETD2 were synthesized by Sangon (Shanghai, China). shRNAs targeting these genes were designed according to siRNAs or BLOCK-iT™ RNAi Designer (<http://rnaidesigner.thermofisher.com>) and were cloned into the PLKO.1 vector that had been digested with AgeI and EcoRI enzymes. The overexpression plasmids were constructed using the pCDH-CMV-MCS-EF1α-Puro, pCDNA3.1, or pLVX-IRES-neo empty backbone. The Escherichia coli Trelief® 5α Chemically Competent Cell (Tsingke, China, Cat# DZC101-96A100) was utilized for cloning applications. The plasmids were transfected into renal cancer cells using Lipofectamine™ LTX (Invitrogen, 15338100, USA) and into HEK293T cells using lipoD293^TM^ (SignaGen, #SL100668, USA), and siRNA was transfected into the cells using GeneMute^TM^ (SignaGen, #SL100568) according to the manufacturer's instructions. sgRNA sequences were designed using CRISPR-ERA (http://crispr-era.stanford.edu/index.jsp), Sequence Scan for CRISPR (http://crispr.dfci.harvard.edu/SSC/), and CRISPOR (https://crispor.gi.ucsc.edu). sgRNA oligos were constructed into LentiCRISPR-V2 and co-transfected with lentiviral packaging plasmids psPAX2 and pMD2.G into HEK293T cells. Cell supernatants containing the lentivirus were collected 48 h after transfection and used to infect RCC cells for 6-18h, with stably transfected cells selected by puromycin. siSKA3 sequence were: sense: 5’-GAGUGUGUAACUCCUAAAU-3’, antisense: 5’-AUUUAGGAGUUACACACUC-3’. Other sequences of the siRNAs/ shRNAs/ sgRNAs used in this article are listed in Supplementary Table S2).

**Generation of knockout cells**

CRISPR/Cas9-mediated gene knockout using the lentiCRISPR-V2 plasmid involves designing a target-specific sgRNA cloning annealed oligonucleotides into the BsmBI-digested plasmid, and validating constructs via sequencing. Lentivirus is produced by co-transfecting HEK293T cells with lentiCRISPR-V2, psPAX2, and pMD2.G plasmids, followed by supernatant collection and filtration. Target cells are transduced with viral particles in the presence of polybrene, selected with puromycin, and monoclonal populations are isolated. Knockout efficiency is confirmed by genomic PCR assay, Sanger sequencing, and western blot to verify frameshift formation and protein loss.

**RNA extraction, reverse transcription and real-time quantitative PCR (RT-qPCR)**

Total RNA was extracted the Total RNA Isolation Reagent (Pufei, Shanghai, China). cDNAs were synthesized using HiScript II Reverse Transcriptase (Vazyme, R223, China) according to the manufacturer’s instructions. Real-time quantitative polymerase chain reaction (RT-qPCR) was performed using ChamQ Universal SYBR qPCR Master Mix (Vazyme, Q711). The relative mRNA expression levels were calculated by the 2^-ΔΔCT^ method and normalized to the expression of GAPDH. Primers used are listed in Supplementary Table S3.

**Western blotting**

Cells were lysed with RIPA Lysis Buffer (Beyotime, P1003) containing a proteinase inhibitor cocktail (Roche, 11836170001, Switzerland) and phosphatase inhibitor cocktail (Roche, 4906845001) to extract total proteins. Cell lysates were incubated on ice for 30 min on rotator, and then centrifuged in a centrifuge at 4 ℃ at 17,000 × g. The supernatant was then transferred to a pre-chilled centrifuge tube. Proteins were quantified the by Pierce BCA Protein Assay Kit (Thermo Fisher, A55865) and then heat-denatured at 95 ℃ for 5 min with loading buffer (Reducing). Equal amounts of proteins (30 - 50 μg) were separated by SDS-PAGE and transferred to nitrocellulose membranes (Millipore, HATF00010, Germany). Then, the membranes were blocked with 5% non-fat milk/ bovine serum albumin (BSA) at room temperature for 1 h and incubated with primary antibodies. After incubation, the membranes were washed with TBS-T for three times (10 min/wash) and incubated with IRDye 800CW/680RD secondary antibodies (LI-CORbio, USA) for 1 h. After washing with TBS-T for three times, the membranes were visualized using an Odyssey DLx (LI-CORbio, USA). The primary antibodies include SKA3 (Thermo, A304-215, 1:1000, RRID: AB_2620412), BAP1 (Santa Cruz, sc28383, 1:200, UK, RRID: AB_626723), β-actin (Proteintech, 66009, 1:20000, China, RRID: AB_2687938), FLAG-M2 (Sigma-Aldrich, F1804, 1:1000, USA, RRID: AB_262044), NCOR1 (Cell signaling Technology, #34271, 1:1000, USA, RRID: AB_2799050), Ubiquitin (Abclonal, A19686, 1:1000, China, RRID: AB_2862735), HA-Tag (Abclonal, AE105, 1:10000, RRID: AB_1549585), TRIM25 (Proteintech, 12573-1-AP, 1:1000, RRID: AB_2209732), MYC-Tag (Abclonal, AE070, 1:2000, RRID: AB_2863795), cGAS (Cell signaling Technology, #79978, 1:1000, RRID: AB_2905508), STING (CST, # 50494, 1:1000, RRID: AB_2799375), GAPDH (Proteintech, 60004, 1:10000, RRID: AB_2107436), Histone H3 (Proteintech, 68345, 1:5000, RRID: AB_3086558), Phospho-STING (Ser366) (Cell signaling Technology, #50907, 1:1000, RRID: AB_2799831).

**Immunofluorescence assay**

An appropriate number of cells were seeded onto cell coverslips in culture dishes and incubated for a specific duration until 80% confluence. For staining of SKA3, centrosomes, and tubulin, cells were fixed with pre-cooled methanol at -20 ℃ for 15 min. For staining of H2AK119ub, BAP1, NCOR1, and TRIM25, cells were fixed using 4% paraformaldehyde. Subsequently, cells were permeabilized with PBST buffer containing 0.2% Triton X-100 at room temperature for 5 min. The cells were blocked with PBST solution supplemented with 5% goat serum at room temperature for 30 minutes. Primary antibodies were diluted in PBST buffer containing 1% BSA and incubated with the cells overnight at 4°C. The primary antibodies include α-tubulin (Sigma-Aldrich, T9026, 1:800), γ-tubulin (Abclonal, A9657, 1:100), BAP1 (Santa Cruz, sc28383, 1:50), FLAG-M2 (Sigma-Aldrich, F1804, 1:1000, USA), NCOR1 (Cell Signaling Technology, #34271, 1:1000), cGAS (Cell Signaling Technology, #79978, 1:1000), STING (CST, #50494, 1:500). The cells were then incubated with fluorescent secondary antibodies in the dark. Cells were mounted using mounting medium containing DAPI and antifade reagent (Lablead, China). Imaging was conducted using a high-resolution confocal microscope (Leica Stellaris 8, Germany) or OLYMPUS Spin10 (CSU-W1) (OLYMPUS). Cell counting and quantitative analysis of fluorescence intensity were performed using ImageJ software (V1.54, RRID:SCR_003070) and LAS X software (RRID:SCR_013673).

**Immunoprecipitation assay**

Cells were washed gently with prechilled PBS, and lysed by IP lysis buffer (25mM Tris-HCl pH7.4, NaCl 150mM, 1% Triton X-100, 1 mM EDTA), added with cOmplete™ protease inhibitor cocktail (Roche), followed by incubation on ice for 30 min with vortexing every 5 min for thorough mixing. Centrifugation at 13,300 rpm for 10 min at 4 ℃ was then performed to collect the supernatant, and 1/10 of the supernatant volume was taken as the "input" sample. Pre-prepared antibody-magnetic-beads complexes (DYKDDDDK tag Nanoselector (AlpVHHs, 016-101-003, China), anti-HA magnetic beads (Sigma-Aldrich, #SAE0197) or Pierce™ protein A/G magnetic beads (Thermo Scientific ,88802) were added to the cell lysate, and the mixture was incubated at 4 ℃ for 4 h with rotation. Subsequently, the magnetic beads were captured using a magnetic separation rack, and the supernatant was discarded. The beads were washed three times with wash buffer to remove non-specific bindings. The antibody-protein complexes were eluted from the beads using elution buffer, and then a reducing protein loading buffer was added. The samples were heat-denatured at 95 ℃ for 5 min before performing SDS-PAGE and immunoblotting to detect protein-protein interactions.

**Cell cycle analysis**

5 × 10^5^ cells were seeded in 6 cm cell culture dishes and cultured overnight. To synchronize cells at the G0/G1 phase, cells were rinsed with PBS twice and incubated in medium containing 0.1 % FBS for 24 - 48 h at 37°C under 5% CO₂. After serum starvation, the medium was replaced with complete medium containing 10% FBS to reinitiate cell cycle progression for 18 - 36 h. The synchronized cells were then washed with ice-cold PBS, detached using 0.25% trypsin-EDTA briefly, and pelleted by centrifugation at 200 × g for 5 min at 4 °C. The cells were gentle resuspended in 1 mL 70% chilled ethanol with gently vortexing, followed by incubation at 4°C overnight. Fixed cells were centrifuged at 400 × g for 5 min, washed twice with pre-chilled PBS to remove ethanol, and treated with 500 μL RNase A (100 μg/mL) at 37°C for 30 min. Propidium iodide (PI) staining solution was added to a final concentration of 10 μg/mL, and the cells were incubated in the dark for 30 min at room temperature. The stained suspension was analyzed using Cytoflex S (Beckman).

**Hematoxylin and Eosin Staining (H&E Staining) AND Immunohistochemistry (IHC)**

Mouse and Patients tissues were fixed in 10% neutral formalin and embedded in paraffin. Tissue sections (4 - 5 µm) were deparaffinized in xylene and rehydrated using a graded ethanol series. For H&E staining, sections were stained with hematoxylin for 10 min, rinsed in running tap water, and differentiated. After bluing, the sections were counterstained with eosin for 2 seconds, dehydrated through an ascending ethanol series, cleared in xylene, and mounted with a resinous medium. For IHC staining, endogenous peroxidase activity was blocked with 3% hydrogen peroxide for 10 min. Then, antigen retrieval was performed by heating sections in 10 mM sodium citrate buffer (pH 6.0) or EDTA buffer (pH 9.0) under high pressure for 3 min, followed by incubation with 10% goat serum for 30 min to reduce non-specific binding. Sections were incubated with primary antibody overnight at 4°C, followed by a biotinylated secondary antibody for 1 h at room temperature. Signal detection was performed using a streptavidin-HRP conjugate and DAB substrate, and the sections were counterstained with hematoxylin, dehydrated, cleared in xylene, and mounted with a resinous medium. The sections were subsequently digitized using NanoZoomer digital slide scanner (Hamamatsu, Japan) to facilitate high-resolution imaging and further analyses.

**Chemical treatment**

For MG132 treatment, 20 μM of MG132 (MedChemExpress, USA) was added to the cell culture medium for 6 h. For G2/M phase arrest, 50 ng/ml nocodazole (MCE) was added to the cell culture medium and incubated for 18 h. Following this, the medium was removed and the cells were washed three times with warm PBS. The medium was then replaced with fresh medium, and the cells were allowed to continue culturing for 0-150 min. For the assessment of protein stability, cycloheximide was added to the cell culture medium at a final concentration of 50 μg/ml, and then proteins were extracted at 0, 6, 12, and 18 h post-treatment for subsequent analysis.

**Public Database Analysis**

Clinical and transcriptomic data from The Cancer Genome Atlas Kidney Renal Clear Cell Carcinoma (TCGA-KIRC) cohort were accessed through diverse web-based tools, including UCSC Xena, GEPIA2, and cBioPortal. Other independent ccRCC patient datasets were obtained from the Gene Expression Omnibus (GEO) database. Differentially expressed genes (DEGs) in ccRCC tissues relative to normal tissues in TCGA-KIRC were identified using GEPIA2 and Xena browser, with thresholds of |log2 fold-change (log2FC) | > 1.5 and false discovery rate (FDR) < 0.05. Somatic mutation profiles were extracted from UCSC Xena and cBioPortal to characterize genomic alterations.

Supplementary Figure and Figure legends

Supplementary Figure S1. Computational screening of mitosis-related genes in ccRCC.

**(**A**)** Principal Component Analysis (PCA) of mitosis-related genes expression profiles in tumor tissues and normal renal tissues of 4 independent ccRCC datasets; (B) Unsupervised clustering heatmaps of differentially expressed mitosis-related genes in tumor tissues and normal renal tissues of four independent ccRCC datasets; (C) LASSO coefficients of metastasis-associated genes (supplemental to Figure 1E and 1F).

Supplementary Figure S2. Clinical and prognostic significance of SKA3.

**(**A**)** K-M analysis of OS in ccRCC patients stratified by *TUBA3D*, *DTL*, *CEP55*, and *SKA3* mRNA expression levels. (B) Correlation between *CEP55* mRNA expression and the CA20 and CIN25 gene signatures. (C) Comparison of *SKA3* mRNA expression between tumor tissues and normal kidney tissues in the TCGA-KIRC cohort. (D) *SKA3* mRNA levels in ccRCC samples stratified by metastasis status (M0 vs. M1). (E) *SKA3* mRNA expression levels across different AJCC (2017) clinical stages ((Stages I–IV)) in the TCGA-KIRC cohort. (F) Box plot illustrating SKA3 expression levels of TCGA-KIRC cohort across different Fuhrman nuclear grades. (G) Forest plots of multivariable Cox regression analysis of clinical parameters, *SKA3* and *MKI67* expression affecting OS in the TCGA-KIRC cohort. Data are presented as hazard ratios with 95% confidence intervals.

Data are presented as mean ± SD or median with interquartile range. Statistical analysis: Log-rank test (A), Pearson’s correlation (B), Mann-Whitney U test (C, D), Kruskal-wallis test (E, F), Cox proportional hazards regression analysis (G).

Supplementary Figure S3. SKA3 promote ccRCC cell proliferation and cell cycle progression.

**(**A**)** RT-qPCR analysis of SKA3 mRNA expression levels in five RCC cell lines; (B) Western blot analysis of SKA3 protein expression levels in five RCC cell lines; (C - D) Western blot confirming SKA3 knockdown by siRNA (C) and shRNA (D) in 786-O and OS-RC-2 cells; (E) CCK-8 assay evaluating the effect of SKA3 knockdown on cell proliferation. (F) Flow cytometric analysis of cell cycle distribution in synchronized 786-O cells following SKA3 knockdown in (left), with quantification shown (Right). (G) Western blot demonstrating SKA3 overexpression in 769-P and Caki-1 cells; (H) CCK-8 assay evaluating the effect of SKA3 overexpression on cell proliferation in 769-P and Caki-1 cells. (I) Flow cytometric analysis of cell cycle distribution in synchronized 769-P and Caki-1 cells following SKA3 overexpression (left), with quantification shown (Right).

Data are presented as mean ± SD. The experiments were repeated n = 3 independent times (biological replicates) (A-I).

Statistical analysis: one-way ANOVA (A, E, F), Student’s t-test (H, I).

Supplementary Figure S4. SKA3 does not affect invasion and migration.

(A) Transwell assays to assess the impact of SKA3 knockdown on the invasion and migration abilities of 786-O and OS-RC-2 cells (Left). Quantification of each group (Right). (B) Transwell assays to assess the impact of SKA3 overexpression on the invasion and migration abilities of 769-P and Caki-1 cells, and quantification of each group. (C) Images of subcutaneous xenograft tumors (left) and tumor volume measurements (right) of SKA3 knockdown (*n* = 3).

Data are presented as mean ± SD. The experiments were repeated *n* = 3 independent times (biological replicates) (A-B) or *n* = 3 mice.

Statistical analysis: one-way ANOVA (A), independent Student’s t-test (B, C).

Supplementary Figure S5. SKA3 fails to modulate cGAS-STING-mediated ISG15/IRF3 levels.

**(**A**)** Immunofluorescence staining of α-tubulin (magenta), γ-tubulin (red), and DAPI (blue) in SKA3 overexpressed Caki-1 cells, showing representative images of normal mitosis and CIN (left). Proportion of mitotic metaphase cells exhibiting lagging chromosomes, centrosome amplification, chromosome misalignment and micronuclei formation (right, *n* = 3 biological replicates, with at least 100 cells counted per replicate). Scale bar = 10 μm; (B) Immunofluorescence staining of α-tubulin (magenta), γ-tubulin (red), and DAPI (blue) in SKA3 overexpressed 786-O and OS-RC-2 cells, showing representative images of normal mitosis and CIN (left). Proportion of mitotic metaphase cells exhibiting lagging chromosomes, centrosome amplification, chromosome misalignment (right, *n* = 3 biological replicates, with at least 100 cells counted per group). Scale bar = 10 μm. (C) Representative time-lapse live-cell imaging of mitosis in 769-P cells overexpressing SKA3. Insets highlight lagging chromosomes, and micronuclei. Scale bar = 20 μm. (D) RT-qPCR analysis of *SKA3* mRNA expression levels in 769-P and Caki-1 cells overexpressing SKA3. (E-F) RT-qPCR analysis of *IRF3* and *ISG15* mRNA expression levels in 769-P and Caki-1 cells overexpressing SKA3 (Cells treated with IFN-γ (50 ng/mL) served as a positive control).

Data are presented as mean ± SD. The experiments were repeated n = 3 independent times (biological replicates) (A-F).

Statistical analysis: independent Student’s t-test (D, F), multiple unpaired t-tests (A, B).

Supplementary Figure S6. BAP1 loss induces SKA3 overexpression in ccRCC.

**(**A**)** Violin plots showing *SKA3* mRNA levels in ccRCC samples stratified by somatic mutation status of *VHL*/*PBRM1*/*KDM5C/SETD2*. (B) Violin plots showing *SKA3* mRNA levels in ccRCC sample with *VHL*/*PBRM1*/*KDM5C/SETD2/BAP1* somatic mutations, categorized into driver mutations and variants of uncertain significance (VUS). (C) K - M analysis of OS in ccRCC patients with VHL/PBRM1/KDM5C/SETD2 somatic mutations of TCGA-KIRC cohort. (D) K - M analysis of OS in ccRCC patients with VHL/PBRM1/KDM5C/SETD2 somatic mutations of TRACERx Renal cohort. (E) BAP1 mutation / copy-number status of TCGA-KIRC cohort.

Data are presented as mean ± SD.

Statistical analysis: Log-rank test (C, D), Mann-Whitney U test (A), Kruskal-Wallis test (B).

Supplementary Figure S7. BAP1 deficiency promotes proliferation and CIN of ccRCC via SKA3.

(A) RT-qPCR analysis of *SKA3* mRNA levels in SETD2-knockdown 769-P and 786-O cells. (B) RT-qPCR analysis of *BAP1* mRNA levels in BAP1 knockdown 786-O and Caki-1 cells. (C) Western blot analysis of SKA3 protein level in BAP1 knockdown 769-P and OS-RC-2 cells. (D) Dual-luciferase reporter assay evaluating the effect of BAP1 overexpression on *SKA3* promoter activity in 786-O cells. (E) Protein stability analysis of SKA3 in BAP1-KO cells reconstituted with wild-type or DUB-domain-deficient BAP1 (ΔDUB) (left). Relative quantification of SKA3 expression levels over time (right). (F) Representative immunofluorescence images demonstrating the effect of SKA3 depletion on CIN in BAP1-knockdown 786-O cells. (G) CCK-8 assay assessing the impact of BAP1 knockdown on proliferation in SKA3-silenced ccRCC cells.

Data are presented as mean ± SD. The experiments were repeated n = 3 independent times (biological replicates) (A-C, E-G), n = 4 independent times (biological replicates) (D).

Statistical analysis: two-way ANOVA (D), one-way ANOVA test (A, B, E, G).

Supplementary Figure S8. BAP1 fails to modulate H2AK119ub enrichment at the *SKA3* promoter.

**(**A**)** ChIP-seq profiles of H2AK119ub enrichment at the *SKA3* promoter region in BAP1-deficient cells overexpressing wild-type or mutant BAP1 (GSE101987 dataset). (B) ChIP-qPCR analysis of H2AK119ub levels at the *SKA3* promoter in BAP1-knockdown 786-O cells.

Data are presented as mean ± SD. The experiments were repeated *n* = 3 independent times (biological replicates) (B).

Statistical analysis: independent Student’s *t* test (B).

Supplementary Figure S9. BAP1 facilitates NCOR1 deubiquitination through DUB activity.

**(**A**)** Pulldown assay showing that the interaction between Flag-NCOR1 and HA-BAP1. (B) Immunofluorescence showing subcellular localization analysis of BAP1 and NCOR1 in 786-O and OSRC-2 cells. (C) Western blot analysis of NCOR1 expression in MOCK or BAP1-KO OS-RC-2 and 786-O cells. (D) Ubiquitination assay showing that BAP1 overexpression reduces NCOR1 ubiquitination level. (E) Ubiquitination assay showing that wild-type BAP1, but not the catalytically inactive mutant (C91A), promotes NCOR1 deubiquitination. (F) Schematic representation of the domain structure of the human BAP1 protein. (G) Co-IP showing that wild-type full-length of BAP1, but not the DUB-mutant or truncated variant, promotes NCOR1 deubiquitination. (H) ChIP-qPCR analysis of NCOR1 enrichment at the *SKA3* promoter in BAP1-KO cells with wild-type or DUB-domain-deficient BAP1 re-expression. (I) Western blot analysis of H3K27ac levels in NCOR1-KO OS-RC-2 cells. (J) RT-qPCR analysis of BAP1 mRNA levels in NCOR1-KO OS-RC-2 cells with BAP1 knockdown.

Data are presented as mean ± SD. The experiments were repeated *n* = 3 independent times (biological replicates) (A-E, G-J).

Statistical analysis: two-way ANOVA (H), independent Student’s *t* test (J).

Supplementary Figure S10. BAP1 promotes TRIM25 expression to drive SKA3 degradation.

(A) Immunofluorescence showing subcellular localization analysis of BAP1 and SKA3 in 786-O cells at different cell cycle phase. (B) Silver-stained SDS-PAGE gel showing proteins co- immunoprecipitated with SKA3, the band corresponding to TRIM25 is indicated. (C) Co-IP assay demonstrating the interaction between HA-TRIM25 and FLAG-SKA3. (D) Co-IP assay showing FLAG-ubiquitin and SKA3 interaction in TRIM25-overexpressed cells. (E) ChIP-seq peaks of H2AK119ub at the *TRIM25* genome region in BAP1-deficient cells overexpressing wild-type or mutant BAP1 (GSE101987 dataset). (F) ChIP-qPCR analysis of H2AK119ub enrichment at the *TRIM25* promoter in BAP1-KO OS-RC-2 cells with wild-type or DUB-domain-deficient BAP1 re-expression. (G) RT-qPCR analysis of TRIM25 in BAP1-KO OS-RC-2 cells with wild-type or DUB-domain-deficient BAP1 re-expression. (H) Protein stability analysis of SKA3 in MOCK and BAP1-KO cells with or without TRIM25 knockdown (left). Relative quantification of SKA3 expression levels over time (right).

Data are presented as mean ± SD. The experiments were repeated *n* = 3 independent times (biological replicates) (A-D, F-H).

Statistical analysis: one-way ANOVA test (G), two-way ANOVA (F).

Supplementary Figure S11. BAP1 promotes TRIM25 expression through PR-DUB activity.

(A) Western blot analysis of ectopic BAP1-dCas9 fusion protein expression in 786-O and 769-P cells using a BAP1-specific antibody. (B) Immunofluorescence staining of subcellular localization of FLAG-tagged BAP1-dCas9 fusion protein and H2AK119ub. (C) ChIP-qPCR analysis of H2AK119ub levels at the *TRIM25* promoter in BAP1-dCas9 ectopic expressed 786-O and 769-P cells transfected with specific sgRNAs. (D) Correlation analysis of BAP1 and TRIM25 mRNA expression in KIRC tumor samples from TCGA database.

Data are presented as mean ± SD. The experiments were repeated *n* = 3 independent times (biological replicates) (A-C).

Statistical analysis: independent Student’s *t* test (C), Spearman's rank correlation coefficient (D).

Supplementary Figure S12. BAP1 promotes genomic damage in ccRCC cells.

(A) Dose-response curves of 769-P and Caki-1 cell lines for the EV and SKA3-OE groups to sunitinib (72 h). (B) Dose-response curves of 769-P and Caki-1 cell lines for the EV and SKA3-OE groups to pazopanib (72 h). (C) Dose-response curves of 769-P and Caki-1 cell lines for the EV and SKA3-OE groups to MPS1 inhibitor AZ3146 (96 h). (D) Dose-response curves of 769-P and Caki-1 cell lines for the EV and SKA3-OE groups to KIF18A inhibitor BTB1 (96 h). (E) Immunofluorescence staining of γH2A.X (phospho-S139) in MOCK and BAP1-KO cells. (F) Comet assays demonstrating DNA damage levels in MOCK and BAP1-KO cells. Representative images are shown (left). (G) Representative images of chromosomal spreads of MOCK and BAP1-KO cells (left). In-serts indicate chromosomal aberration. Quantification of the percentage of metaphase cells with chromosomal aberration is presented in the right panel. Chromosome aberrations were counted in a minimum of 100 metaphase cells in each group. Results are pooled from three independent biological experiments. Scale bars = 2 μm. 

Data are presented as mean ± SD. The experiments were repeated *n* = 3 independent times (biological replicates) with 4 technical replicates (A- D). The experiments were repeated *n* = 3 independent times (biological replicates) (E - G).

Statistical analysis: independent student’s t-test (F, G).

Supplementary Figure S13. BAP1 and SKA3 regulate genomic damage in ccRCC

(A) Representative m-IHC image of BAP1 (red), cGAS (green), γH2A.X (yellow) in ccRCC tissue. Scale bar = 500 μm. Box plots (bottom) shows the proportion of γH2A.X-positive cells, cGAS-positive cells (n = 10 patients each group). (B) Immunofluorescence staining of γH2A.X in SKA3-OE cells with or without KIF18Ai (AMG650) treatment (left); Quantification of nuclear γH2A.X foci number per cell in each group. At least 50 cells in each group. Results are pooled from three independent biological experiments.

Data are presented as mean ± SD. The experiments were repeated *n*=10 clinical samples(A) or *n* = 3 independent times (biological replicates) (B).

Statistical analysis: independent student’s t-test (A), one-way ANOVA (B).

Table S1 (separate file)

591 mitosis-associated genes.

Table S2: The oligonucleotide Squence of siRNA/sgRNA.

| Oligo Name |  | Sequence |
| --- | --- | --- |
| siNCOR1-1 |  | GGAGAAUAAUGAGAAACAAAUTT |
| siNCOR1-2 |  | GACGAGUCAAGUUCAUUAACATT |
| siSETD2-1 |  | GAUAGCCAUGAUAGUAUUATT |
| siSETD2-2 |  | GGGAGAACAGGCGUAAUAATT |
| siBAP1-1 |  | CGGCCUUUCUAGACAAUCATT |
| siBAP1-2 |  | GCAGCUGAUAAGAGUAACATT |
| siSKA3-1 |  | GAGUGUGUAACUCCUAAAUTT |
| siSKA3-2 |  | GAACACCAGUUAAACUAAUTT |
| siSKA3-3 |  | CCAGAAGUAUGGAUAUAGUTT |
| siTRIM25-1 |  | GAGUGAGAUCCAGACCUUGAATT |
| siTRIM25-2 |  | GCAAGUUUGACACCAUUUAUCTT |
| sgNCOR1-1 | Forward | CACCgaagcgaccacgtctggaac |
| sgNCOR1-1 | Reverse | AAACgttccagacgtggtcgcttc |
| sgNCOR1-3 | Forward | CACCGtctggcagcgggtgcactaa |
| sgNCOR1-3 | Reverse | AAACttagtgcacccgctgccagaC |
| sgCGAS-1 | Forward | CACCGcggcccccattctcgtacgg |
| sgCGAS-1 | Reverse | AAACccgtacgagaatgggggccgC |
| sgCGAS-2 | Forward | CACCGccgcgatgatatctccacgg |
| sgCGAS-2 | Reverse | AAACccgtggagatatcatcgcggC |

Table S3: The oligonucleotide Squence of qPCR primers

| Gene Name | Species | F/R | sequence |
| --- | --- | --- | --- |
| SKA3 | Human | Forward | TACACGAGCAAGAAGCCATTAAC |
|  |  | Reverse | GGATACGATGTACCGCTCAAGT |
| BAP1 | Human | Forward | TTCAGCCCTGAGAGCAAAGG |
|  |  | Reverse | TGAAGCGGATGTCGTGGTAG |
| TRIM25 | Human | Forward | CTGCGGGAATTTTTCTGCCC |
|  |  | Reverse | GTTTGCAGTCATCCGCACAT |
| NCCRP1 | Human | Forward | CCCTGGAAACTCTGGGCAAT |
|  |  | Reverse | CATGCAGCTCATAGACGCAC |
| OBI1 | Human | Forward | GCTCAGACCGTGCAGAATGT |
|  |  | Reverse | GACAGGCTGACGTACCTTCC |
| KCTD5 | Human | Forward | GAGAATCACTGCGAGCTCCT |
|  |  | Reverse | AGGCGCCTGTTTCATCCTTG |
| PBK | Human | Forward | AGCCAATGATGGCAGTCTGT |
|  |  | Reverse | CTCAGGGTCAGTCACAGTCAT |
| CUL1 | Human | Forward | CTACTGATTCAGGCGGCCAT |
|  |  | Reverse | GACCTCGCCAAGTAACTGCT |
| TRIP4 | Human | Forward | GGAGATCATTCAGTACGTTTTGTCA |
|  |  | Reverse | GCTGTTCTCTTGTGCCTTGG |
| TRIM21 | Human | Forward | TGTCCTGGAAAGGAGTGAGT |
|  |  | Reverse | TGGACTGCACATGTCCTCAG |
| NCOR1 | Human | Forward | CCATAATGCAGGGGACACCA |
|  |  | Reverse | TCAATTATCGGCCGTGTGCT |

Table S4. Multivariable Cox Regression Models for Assessing the Proliferation-Independent Prognostic Value of SKA3 in ccRCC

|  | **Model 1: Interaction Analysis** | | | **Model 2: Proliferation-Depleted Residual Analysis** | | |
| --- | --- | --- | --- | --- | --- | --- |
| **Characteristic** | **HR** | **95% CI** | **p-value** | **HR** | **95% CI** | **p-value** |
| Age (*N* = 492) | 1.03 | 1.02, 1.05 | **<0.001** | 1.03 | 1.02, 1.05 | **<0.001** |
| Sex |  |  |  |  |  |  |
| male (*N* = 324) | — | — |  | — | — |  |
| female (*N* = 168) | 0.93 | 0.67, 1.29 | 0.665 | 0.94 | 0.68, 1.30 | 0.719 |
| AJCC Stage |  |  |  |  |  |  |
| I and II (*N* = 291) | — | — |  | — | — |  |
| III and IV (*N* = 201) | 3.26 | 2.33, 4.58 | **<0.001** | 3.32 | 2.37, 4.64 | **<0.001** |
| *SKA3 (N* = 492*)* | 1.07 | 0.48, 2.38 | 0.867 |  |  |  |
| *MKI67 (N* = 492*)* | 0.97 | 0.64, 1.49 | 0.905 | 1.55 | 1.26, 1.91 | **<0.001** |
| *SKA3* × *MKI67* | 1.19 | 0.96, 1.49 | 0.119 |  |  |  |
| *SKA3*_residual |  |  |  | 1.71 | 1.03, 2.83 | **0.037** |
| Abbreviations: CI = Confidence Interval, HR = Hazard Ratio | | | | | | |

Movie S1 - S3. (separate file)

Movie S1. Time-lapse living imaging of RCC cells with EV.

Movie S2. Time-lapse living imaging of RCC cells with SKA3-GFP showing micronuclei and lagging chromosome.

Movie S3. Time-lapse living imaging of RCC cells with SKA3-GFP showing micronuclei and lagging chromosome.

Data S1. (separate file)

Source data.
